# Comprehensive *O*-Glycan Analysis by Porous Graphitized Carbon Nanoliquid Chromatography–Mass Spectrometry

**DOI:** 10.1021/acs.analchem.3c05826

**Published:** 2024-05-17

**Authors:** Tao Zhang, Wenjun Wang, Manfred Wuhrer, Noortje de Haan

**Affiliations:** Center for Proteomics and Metabolomics, Leiden University Medical Center, P.O. Box 9600, Leiden 2300 RC, The Netherlands

## Abstract

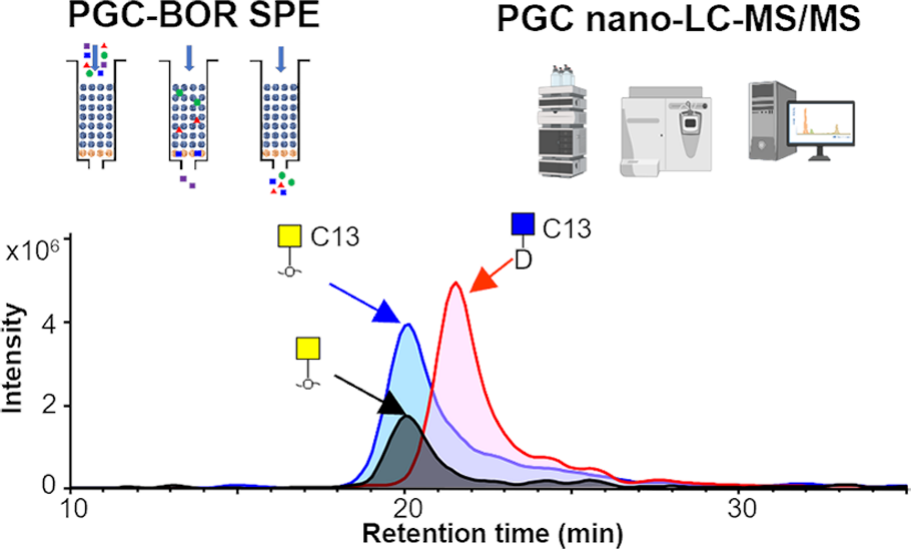

The diverse and unpredictable structures of *O*-GalNAc-type protein glycosylation present a challenge for its structural and functional characterization in a biological system. Porous graphitized carbon (PGC) liquid chromatography (LC) coupled to mass spectrometry (MS) has become one of the most powerful methods for the global analysis of glycans in complex biological samples, mainly due to the extensive chromatographic separation of (isomeric) glycan structures and the information delivered by collision induced fragmentation in negative mode MS for structural elucidation. However, current PGC-based methodologies fail to detect the smaller glycan species consisting of one or two monosaccharides, such as the Tn (single GalNAc) antigen, which is broadly implicated in cancer biology. This limitation is caused by the loss of small saccharides during sample preparation and LC. Here, we improved the conventional PGC nano-LC-MS/MS-based strategy for *O*-glycan analysis, enabling the detection of truncated *O*-glycan species and improving isomer separation. This was achieved by the implementation of 2.7 μm PGC particles in both the trap and analytical LC columns, which provided an enhanced binding capacity and isomer separation for *O*-glycans. Furthermore, a novel mixed-mode PGC-boronic acid-solid phase extraction during sample preparation was established to purify a broad range of glycans in an unbiased manner, including the previously missed mono- and disaccharides. Taken together, the optimized PGC nano-LC-MS/MS platform presents a powerful component of the toolbox for comprehensive *O*-glycan characterization.

## Introduction

Protein glycosylation is involved in many biological processes such as cellular signaling, immune responses, and cancer progression.^1−3^ In particular, *O*-GalNAc-glycosylation (hereafter *O*-glycosylation), characterized by an initiating *N*-acetylgalactosamine (GalNAc) attached to serine, threonine, or tyrosine residues, plays key roles in regulation of protein function, modulation of cell signaling and cell–cell interactions.^4,5^ Yet, little is known about exact structure–function relationships of *O*-glycosylation as *O*-glycan structures are extremely diverse, varying vastly in size, monosaccharide composition, branching, and functional decoration via, e.g., sialic acids and fucoses.

Motivated by the urge to understand *O*-glycan regulation and function at the cellular level, many analytical strategies targeting these molecules have been developed in recent years.^6−8^ In these efforts, mass-spectrometry-based glycomics has become one of the most powerful methods for the global analysis of glycans in complex biological samples. To obtain the deepest structural insights in protein *O*-glycans, the oligosaccharide are usually released from their protein carrier, taking advantages of 96-well plate sample preparation and structural characterization provided by tandem MS.^9−12^ A proven approach for in-depth *O*-glycan analysis is based on porous graphitized carbon nanoliquid chromatography coupled to negative mode tandem mass spectrometry (PGC nano-LC-MS/MS) as initially established by Packer and co-workers.^13−15^ This analytical platform for glycan alditols features extensive glycan isomer separation as well as negative mode collision induced dissociation (CID) resulting in cross-ring fragments to help to elucidate glycan structures. The method was adapted by many laboratories,^16−18^ including ours, and previously we developed a 96-well plate sample preparation for integrated *N*- and *O*-glycomics based on PGC nano-LC-MS/MS, already applied for hundreds of samples.^12,19,20^

Despite the clear advantages, current PGC-based methodologies fail to detect the smaller glycan species consisting of one or two monosaccharides, such as the Tn (GalNAc) antigen, which are broadly implicated in cancer biology and the development of immunotherapy.^21−24^ The reason for this is that unmodified mono- or disaccharides are minimally trapped on PGC material, both during the sample preparation as well with the online PGC LC.^25^ Previously, these challenges were addressed by using alternative analytical platforms. Matrix-assisted laser desorption/ionization (MALDI)-MS has been successfully applied to analyze permethylated *O*-glycans, covering HexNAc monomers up to *O*-glycan decamers.^26^ However, MALDI does not allow the online coupling of a separation module, hence providing limited information on glycan structures. For example, GalNAc monomers could not be discriminated from the isomeric *N*-acetylglucosamine (GlcNAc) monomers derived from an alternative glycosylation pathway. To overcome the latter, we recently established a methodology using reducing-end labeling to efficiently trap and separate glycan isomer (including HexNAc monomers) on C18 nano-LC coupled to positive mode MS/MS.^27^ While exhibiting significant synergy with established PGC-based approaches in terms of stability and throughput, this method does necessitate glycan labeling, a process that introduces potential sample loss. Furthermore, this method is presently limited to positive mode MS. We here aimed to strengthen the PGC glycomics platform and widen its scope to enable negative mode MS characterization of a diverse spectrum of *O*-glycans, including the truncated ones, without the need of any labeling.

To achieve this, we focused on both the sample preparation and nano-LC. First, a novel mixed-mode PGC-boronic acid solid phase extraction (PGC-BOR SPE) was established to achieve close to 100% recovery for all *O*-glycan species, including GalNAc and GlcNAc alditols. Next, the implementation of newly available 2.7 μm PGC particles with a narrow particle size distribution (PSD) in both trap column and analytical column resulted in an improved binding capacity for truncated *O*-glycans as well as enhanced isomer separation across the entire range of glycan structures. The full workflow was validated on well characterized glycan standards as well as on lysates of the pancreatic cancer cell line PaTu-S. The achieved improvement of the PGC nano-LC-MS/MS platform are crucial for its future application on pressing questions in cancer glycobiology and beyond.

## Materials and Methods

### Cells, Chemicals, and Released Glycans

PaTu-8988S (PaTu-S) cells were cultured, O-glycans were released, and standards were prepared as described previously^12^ and detailed in the Supporting Experimental Section.

### Combined PGC-BOR SPE for *O*-Glycan Purification

*O*-Glycan purification using combined PGC-BOR SPE was performed on a 96-well filter plate. For the optimized protocol, 10 μL of the immobilized BOR resin slurry (50%, 20244, Thermo Fisher Scientific) was added to each well in the filter plate and packed by centrifuging at 200*g* for 1 min. Next, 90 μL of bulk sorbent carbograph slurry in 50% (v/v) methanol was packed to the same well by centrifugation under the same conditions. The columns were preconditioned by 1 × 100 μL of 100 mM formic acid (FA), 2 × 100 μL of 80% acetonitrile (ACN), and 1 × 100 μL of 200 mM ammonium bicarbonate (ABC, pH8.8), each time followed by centrifuging at 500*g* for 1 min. The samples were mixed with 10 μL of 400 mM ABC (pH8.8), loaded onto the columns and washed 2× with 100 μL of 200 mM ABC (pH8.8) and 1× with 100 μL of water, each step followed by centrifugation at 500*g* for 1 min. Next, the *O*-glycan alditols were sequentially eluted by 1 × 100 μL of 100 mM FA, 1 × 100 μL of 60% ACN with 0.1% trifluoroacetic acid (TFA), and 1 × 100 μL of 25 mM hydrochloric acid (HCl), centrifuging for 2 min at 800*g*. The eluants were combined and dried in a SpeedVac concentrator at 35 °C. Prior to PGC nano-LC-MS/MS analysis, the samples were resuspended in 10 μL of water. The optimization conditions of the PGC-BOR SPE can be found in the Supporting Experimental Section.

### PGC Nano-LC-MS/MS of *O*-Glycan Alditols Using 2.7 μm PGC Particles

Analysis was performed using a PGC nano-LC Ultimate 3000 UHPLC system (Thermo Fisher Scientific, Sunnyvale, CA) coupled to an amaZon ETD speed ion trap (Bruker Daltonics, Bremen, Germany). Trap columns with 320 μm inner diameter and different length (4, 6, and 8 cm) and a separation column (75 μm × 15 cm) were home-packed with 2.7 μm PGC particles derived from the Supel Carbon analytical column (5 cm × 2.1 mm; Merck). The LC system was coupled to an amaZon ETD speed ESI ion trap MS using the CaptiveSpray source (Bruker Daltonics), which was used in negative ionization mode. In the optimized method, mobile phase A consisted of 10 mM ABC, while mobile phase B was 60% (v/v) acetonitrile/10 mM ABC. To analyze *O*-glycans, 1 μL injections were performed, and trapping was achieved on the trap column using a 6 μL/min loading flow in 5% buffer B for 3 min. Separation was achieved with a multistep gradient of B: 5–5% in 20 min and 5–69% over 80 min followed by a 10 min wash step using 95% of B at a flow rate of 0.6 μL/min. The column was held at a constant temperature of 30 °C. The optimization conditions for the nano-LC can be found in the Supporting Experimental Section.

### Data Analysis

Glycan structures were assigned on the basis of the known MS/MS fragmentation patterns in negative-ion mode,^28,29^ presence of diagnostic ions for structural features, elution order, and general glycobiological knowledge,^1^ with help of the Glycoworkbench^30^ and Glycomod software.^31^ Annotated MS2 spectra are provided in Figure S1. Relative quantification of individual glycans was performed by dividing the absolute peak area of each glycan by the sum of the peak areas of all glycans in the respective sample and multiplying it by 100%. This results in a percent representation of the individual glycans per sample. Standard deviations and CVs were calculated for the replicates. Raw data is available online at Glycopost: https://glycopost.glycosmos.org/entry/GPST000389.

## Results and Discussion

**Figure 1:**
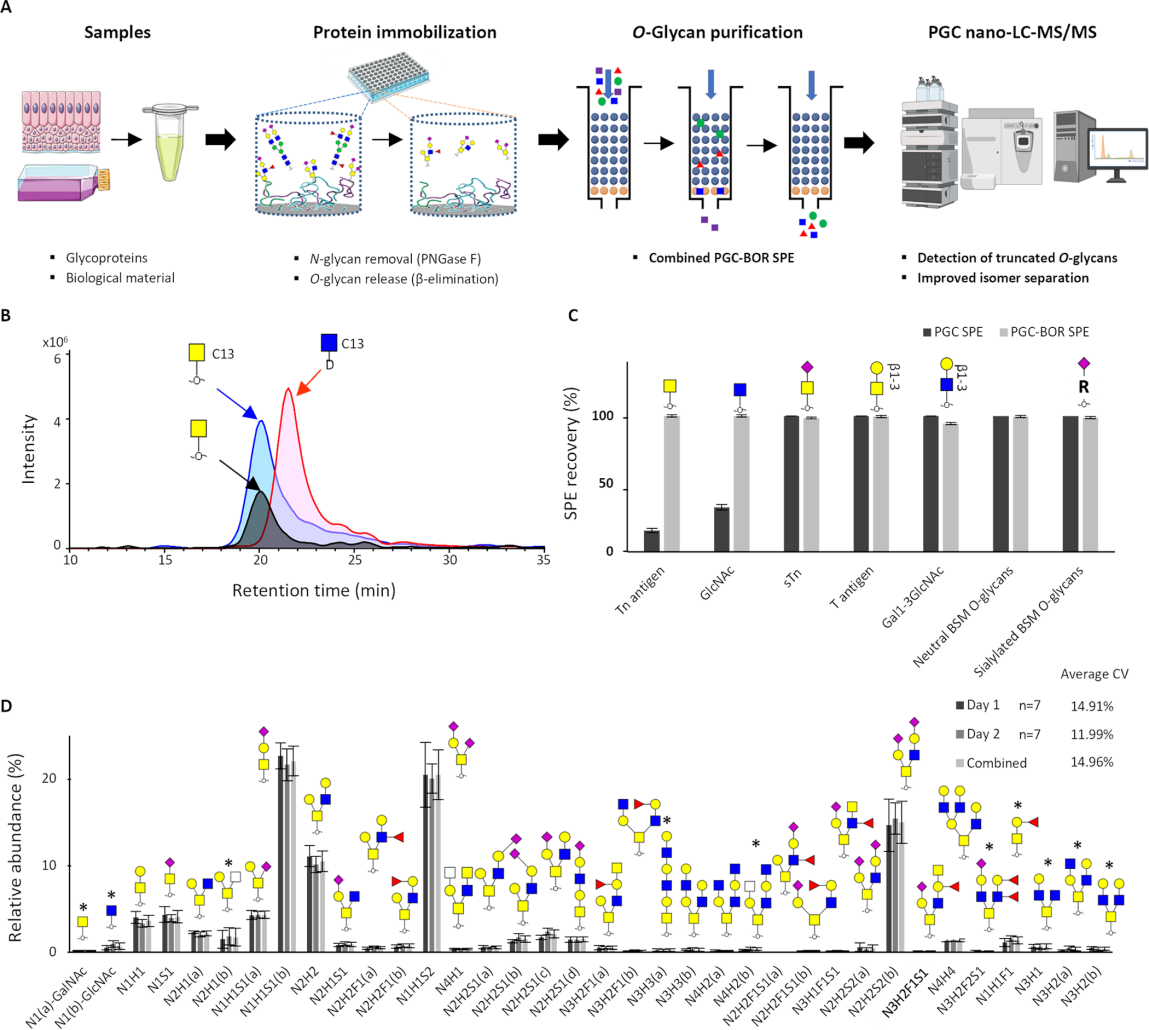
Workflow and performance of the PGC nano-LC-MS/MS platform. (A) Workflow of the optimized method, including protein immobilization, *O*-glycan release, novel mixed-mode PGC-BOR SPE, and improved PGC nano-LC-MS analysis. (B) Separation of GalNAc and GlcNAc alditols using PGC nano-LC-MS with 2.7 μm PGC particles. C13 indicates the isotope ^13^C_6_ labeling of GalNAc and GlcNAc. D indicates a deutero-reduced reducing end. (C) Recovery of PGC-BOR SPE compared to conventional PGC SPE (*n* = 3 per condition). (D) Intra- and interday repeatability of the *O*-glycan analysis from 5 × 10^4^ PaTu-S cells in seven technical replicates on two successive days. Displayed are average relative intensities for the glycans with a relative abundance above 1%, with error bars representing the standard deviations. Average intra- and interday CVs of the top 10 most abundant *O*-glycans are displayed. H, hexose; N, *N*-acetylhexosamine; F, fucose; S, *N*-acetylneuraminic acid. *Newly detected *O*-glycans for PaTu-S cells in this study.

Here, we established a PGC nano-LC-MS/MS-based workflow for *O*-glycan analysis, enabling the detection of truncated *O*-glycan species and showing an improved isomer separation compared to conventional PGC nano-LC-MS/MS approaches (Figure 1A). The sample preparation is performed in 96-well plate format for efficient sample throughput and the method is applicable to μg-levels of purified glycoproteins as well as to biological samples such as cells and tissues.

### Small, 2.7 μm PGC Particles Retain Truncated *O*-Glycans and Allow Extensive Isomer Separation

In this study, newly available 2.7 μm PGC particles with a narrow PSD and high mechanical stability were employed for both the trap and the analytical column of the nano-LC system to achieve the binding of truncated *O*-glycans and to improve isomer separation. Conventional trap columns, packed with larger (e.g., 5 μm) PGC particles, often show limited to no binding of monomeric saccharides such as Tn antigen or *O*-GlcNAc, and low affinity for dimeric structures such as T antigen (Galβ1–3GalNAc-),^17,32,33^ resulting in them eluting in broad and hard to quantify peaks (Figure S2A). Our new trap column retained small glycan structures well, resulting in successful detection of Tn antigen and improved peak shape of T antigen (Figure S2A). Yet, trapping time and temperature are still critical for the successful analysis of truncated *O*-glycans, showing optimal trapping at 30 °C for 4 min (Figure S2B and C).

Next to an improved detection of truncated *O*-glycans, the 2.7 μm particles also resulted in a better separation of isomers on the analytical column after optimization of the starting level of solvent B (Figure S3A), temperature (Figure S3B), trap column length (Figure S3C), and flow rate (Figure S3D). The final conditions, using an 8 cm trap column, starting the gradient at 5% solvent B and having a 0.6 μL/min flow rate at 30 °C, resulted in the partial yet quantifiable separation of GalNAc and GlcNAc alditols. The assignment of these monosaccharide isomers was further substantiated by spiking in reduced ^13^C_6_ isotope labeled GalNAc (*m*/*z* 228.10) and deutero-reduced ^13^C_6_ isotope labeled GlcNAc (*m*/*z* 229.10), showing them to elute 1.5 min apart (Figure 1B). Because of the partial separation of GlcNAC-ol and GalNAc-ol, the generated internal standards are generally required for the annotation of these monomeric species. While the MS2 spectra are highly similar between the two species, consistent abundancy differences were observed for the fragments at *m*/*z* 172.04 and 190.06 (both higher for GlcNAc-ol; Figure S1, origin unknown). Under the same conditions, the full separation of the T antigen alditol from its isomer Galβ1–3GlcNAc-ol was achieved (Figure S4). The MS/MS spectra of the two isomers were shown to be distinct, with a higher abundance of B and C fragments for Galβ1–3GalNAc compared to Galβ1–3GlcNAc (Figure S4C). While peak width and symmetry are not optimal under the current conditions and could be improved using a shorter trap column, higher column temperature, and/or a higher starting concentration of solvent B (Figure S3), the chromatographic resolution between GalNAc and GlcNAc alditols is highest using the optimized parameters.

Previously, ion mobility (IM)-MS has been shown to be able to separate HexNAc epimers based on drift time differences^34^ and 2-aminobenzamide-labeled GalNAc and GlcNAc were separated using C18 nano-LC.^27^ Here, we developed an LC-MS-based platform to separate underivatized *O*-glycan monosaccharide alditols. Importantly, these epimers fulfill significant yet distinct roles in biology. They are incorporated into proteins during their secretion by GalNAc-transferases 1 to 20 (*O*-GalNAc) or EGF domain-specific *O*-GlcNAc transferase (EOGT; *O*-GlcNAc), and alternatively, in the nucleoplasm through *O*-GlcNAc transferase (OGT; *O*-GlcNAc). This urges their discrimination in glycomics experiments.

### Unbiased *O*-Glycan Purification Using Combined PGC-BOR SPE

To address the loss of truncated *O*-glycan species using conventional PGC SPE approaches, we developed a combined PGC-BOR SPE to achieve highly efficient purification for all of the *O*-glycan species. Conventional PGC SPE has been widely used in glycan purification and desalting in different SPE formats, especially for complex biological samples, due to its strong binding to oligosaccharide, following the procedure described by Packer et al.^13^ However, as shown in Figure S5A,C, the conventional PGC approach has limited binding capacity for truncated *O*-glycans, resulting in the loss of more than 85% of Tn antigen and about 5% of T antigen. BOR is an alternative stationary phase for glycan enrichment, covalently binding to *cis-diol* groups to form five- or six-membered cyclic esters under basic conditions, a reaction that is reversible at acidic pH.^35−38^ Though a low binding to sialic acid-containing glycoproteins has been reported,^39,40^ BOR affinity chromatography has been successfully applied in both glycoprotein and glycopeptide enrichment. Previously, the interaction between boronic acid and saccharides in aqueous solution was investigated,^41^ yet the use of BOR affinity SPE for glycan alditol purification has not been studied well.

In this work, we investigated the performance of the BOR SPE for *O*-glycan purification using glycan standards. In contrast to the conventional PGC SPE, BOR SPE demonstrated a close to 100% recovery for the Tn and T antigen alditols (Figure S5C). Of note, BOR SPE also displayed a high recovery for GlcNAc alditols, probably via binding to the 1,2- or 1,3-diols.^41^ However, BOR SPE showed only a 52% recovery of the sialylated Tn antigen (sTn) alditol, indicating a possible negative effect of sialic acids on BOR retention. The lower BOR SPE affinity for sialylated species was further confirmed for larger structures in a BSM-derived *O*-glycan mixture, containing both NeuAc and NeuGc sialylated *O*-glycans (Figures S5B,D and S6). These findings are in line with a previous study showing boronate affinity materials to preferentially bind to nonsialylated glycoproteins.^39,40^

To profit from both specificities, we combined the PGC and BOR SPE modes. Using 10% BOR and 90% PGC material in one SPE column (pipet tip or filter plate format) resulted in a close to 100% recovery for all *O*-glycans in the glycan standards and BSM test sample (Figure 1C and Figure S7), independent of glycan size and sialylation state. Notably, different packing formats of PGC-BOR SPE, especially using a higher ratio of BOR material, led to decreased *O*-glycan recovery (Figure S7B), indicating the importance of the PGC material for SPE of larger *O*-glycans.

### The Optimized PGC Nano-LC-MS/MS Platform Features In-Depth Glycan Characterization in Complex Biological Samples

**Figure 2:**
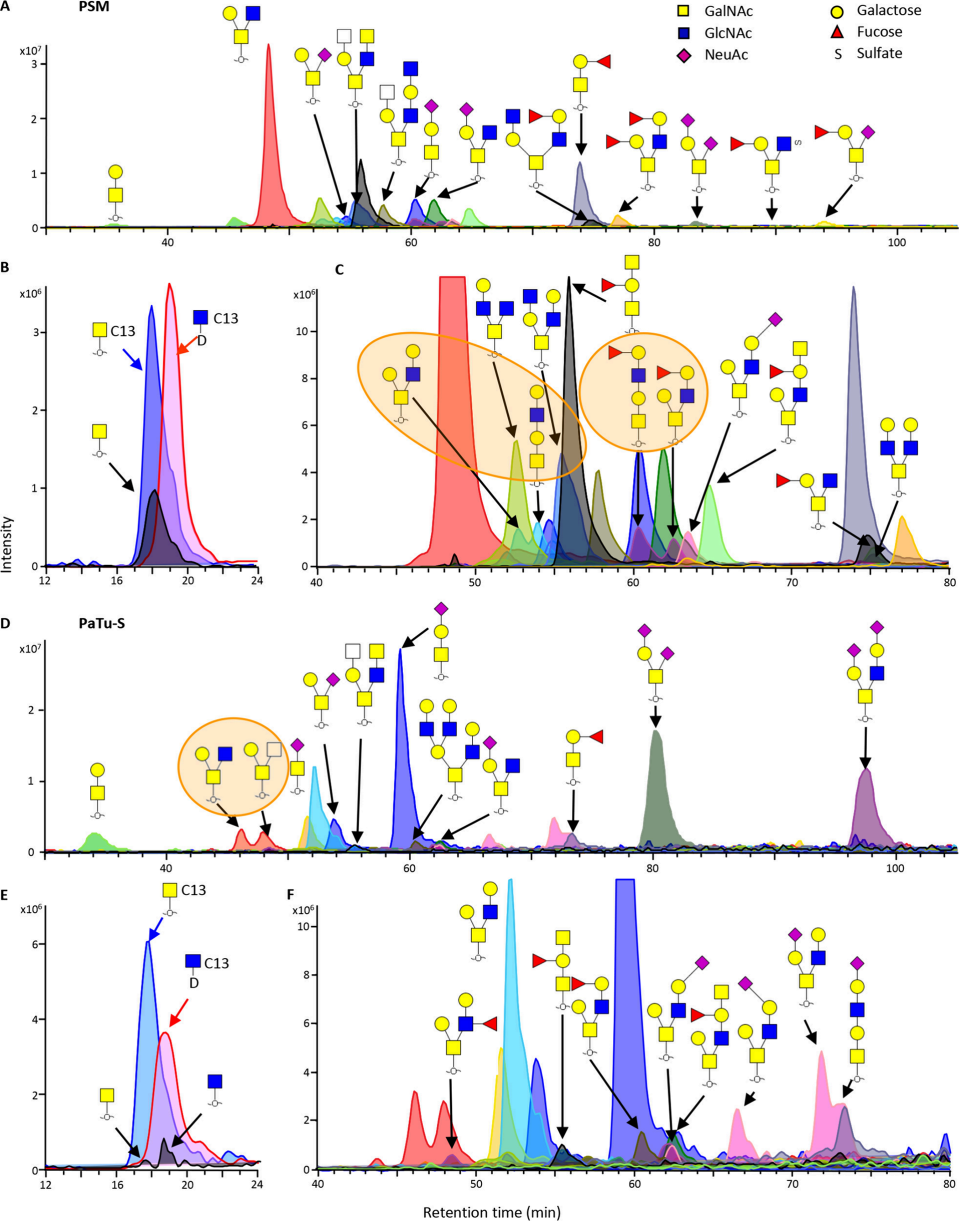
Representative examples of the *O*-glycan analysis of PSM glycoprotein and PaTu-S cell lysate following the established analytical approach. (A) Combined EICs of *O*-glycans released from 1 μg of PSM glycoprotein. (B) Combined EICs of GalNAC and GlcNAc released from PSM glycoprotein, retention time: 12 to 24 min. (C) Combined EICs of low abundant *O*-glycans released from PSM glycoprotein in zoomed-in area, retention time: 40 to 80 min. (D) Combined EICs of *O*-glycans released from 5 × 10^5^ PaTu-S cells. (E) Combined EICs of GalNAC and GlcNAc released from PaTu-S cells, retention time: 12 to 24 min. (F) Combined EICs of low abundant *O*-glycans released from PaTu-S cells in zoomed-in area, retention time: 40 to 80 min. Examples of improved isomer separation are shaded -orange. More details are displayed in Figures S8–S10. C13 indicated the isotope ^13^C_6_ labeling of GalNAc and GlcNAc. D indicates deutero-reduced *O*-glycan. The extracted ion chromatogram for GalNAc and GlcNAc in panel (E) was smoothed using the Gauss smoothing algorithm with an 8.25 smoothing width for 1 cycle.

**Figure 3:**
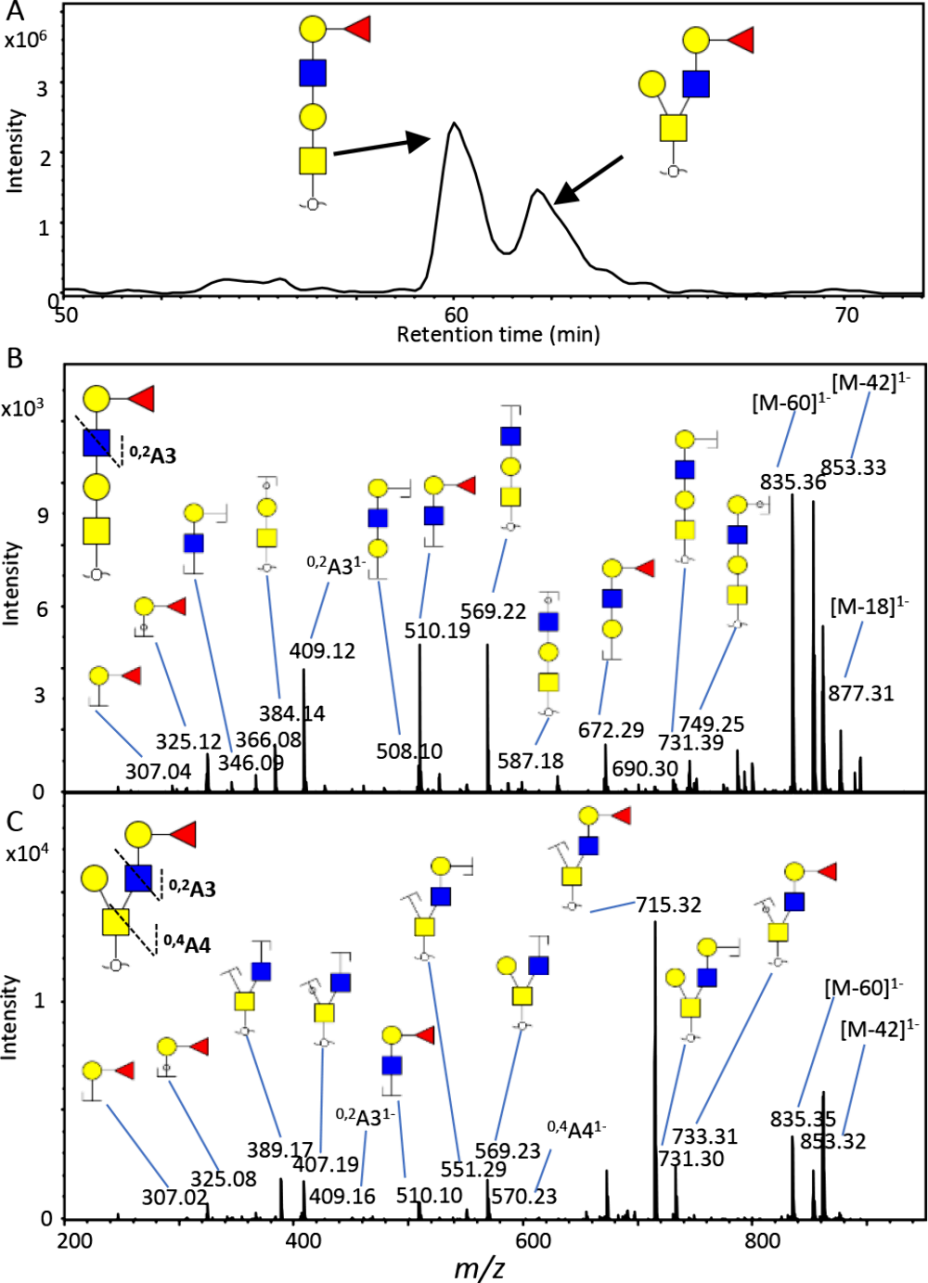
PGC nano-LC separation of two N2H2F1 isomers released from PSM. (A) Improved isomer separation of N2H2F1 isomers was achieved on the PGC nano-LC-MS/MS platform using a trap column (320 μm × 8 cm) and an analytical column (75 μm × 15 cm) packed with 2.7 μm PGC particles. Fragmentation spectra of the early eluting N2H2F1 isomer at 60.2 min (B) and the late-eluting N2H2F1 isomer at 62.3 min (C) indicate a core 1 structure for the early eluting N2H2F_1_ and a core 2 structure for the late-eluting N2H2F1.

Our new developments allow the detection of truncated *O*-glycans and extensive isomer separation without any labeling or derivatization required. Furthermore, the *O*-glycan alditols were analyzed in negative ionization mode, resulting in cross-ring fragmentation during MS/MS and providing diagnostic ions for the characterization of glycan linkages. The capabilities of the optimized method were further explored using porcine submaxillary mucin (PSM) glycoprotein and the pancreatic cancer cell line PaTu-S. PSM has been used as an model *O*-glycan source in method development research before and contains mainly neutral core 2 *O*-glycans.^18,29,42^ Using 1 μg of PSM, 32 *O*-glycans were detected (above 0.5% relative abundance) and quantified, including truncated *O*-glycans, as well as neutral and sialylated *O*-glycans, carrying different levels of fucosylation and sulfation (Figure 2, Table S1). The identity of the HexNAc alditol eluting at 17.4 min was confirmed to be GalNAc-derived using reduced ^13^C_6_ isotope-labeled GalNAc and deutero-reduced ^13^C_6_ isotope-labeled GlcNAc as internal standards (Figure 2B). Also for the larger glycans, an improved isomer separation was observed as compared to that of conventional PGC nano-LC-MS/MS, exemplified by the glycans with compositions N2H2 and N2H2F1 (Figure 2C). The first eluting isomer of N2H2 at 35.2 min was characterized as the core 2 structure based on the presence of a ^0,4^A_3_ cross-ring fragment at *m*/*z* 424.22, while the one at 37.9 min was found to have a core 1 structure (diagnostic B3 ion at *m*/*z* 526.21, Figure S8). Moreover, fucosylated *O*-glycan N2H2F1 isomers featured a linear core 1 structure with type II blood H antigen at 60.2 min, while the glycan at 62.4 min was assigned as a core 2 structure with type II blood H antigen (Figure 3 and Figure S9). These isomers were also reported in other studies where PSM *O*-glycans were analyzed, using high-temperature GC-MS for permethylated *O*-glycans^43^ and using a combination of high-flow PGC LC-MS and IM for *O*-glycans alditols.^18^

The intra- and interday repeatability of the new method was assessed using PaTu-S cells. Analysis of 5 × 10^4^ cells per sample resulted in the identification (above 0.2% relative abundance) and quantification of 23 *O*-glycan compositions and a total of 44 *O*-glycan structures including isomers (Figure 1D, Table S2, Figure S10), with an average CV of the top ten most abundant peaks of 14.9% over 2 days. Compared with the previous characterization of *O*-glycans of the PaTu-S cell line, 10 additional species were detected, including the previously “missing” monomeric *O*-GalNAc and *O*-GlcNAc glycans (Figure 2E). While the GalNAc monomer is likely derived from α-GalNAcylation (catalyzed by the GALNACT enzymes), the sources of the GlcNAc monomer in cells might be diverse. β-GlcNAcylation occurs both intra and extracellularly (catalyzed by the OGT and EOGT enzymes, respectively) and likely contributes to the largest part of the GlcNAc signal.^27^ Interestingly, α-GlcNAcylation has been reported in protozoa, where catalyzed by GALNACT-like enzymes, and its occurrence in human cells should be further investigated.^44^

Other methods capable of detecting and differentiating HexNAc epimers as well as other isomeric structures in complex biological materials are IM-MS and our recently developed C18 nanoLC-MS approach following 2-aminobenzamide labeling of *O*-glycans. The current methodology complements these strategies by omitting the need for derivatization, providing a partly orthogonal separation, and featuring negative ion mode fragmentation to obtain deeper structural insights in (isomeric) glycan structures. It is worth mentioning that synergetic glycoproteomic methodologies using (*O*-glyco)proteases such as IMPa and StcE have successfully identified Tn-containing glycopeptides, providing information on glycosylation sites and occupancy.^45,46^ However, detailed characterization of the *O*-glycan repertoire is not yet feasible when studying glycopeptides.

## Conclusions

In this work, we demonstrated that the use of 2.7 μm PGC particles in both trap- and analytical column provides an enhanced binding capacity, and isomer separation for *O*-glycans using PGC nano-LC. In combination with our novel mixed mode PGC-BOR SPE during the sample preparation, we enabled the previously unseen analysis of underivatized mono- and dimeric *O*-glycans in complex biological samples using PGC nano-LC-MS. Being now able to identify, and relatively quantify, *O*-glycans ranging from monomers to extensively branched and modified (sialylated, fucosylated, sulfated) structures in one analysis as well as providing extensive separation of structural glycan isomers, the optimized PGC nano-LC-MS/MS platform positions as a crucial and complementary component of the toolbox for comprehensive *O*-glycan characterization.

## Supporting Information Available

Details on chemicals, materials, cell culture, glycan preparation, the optimization of PGC-BOR SPE, and the optimization of nano-LC-MS analysis; extended figures of the glycan structure elucidation, chromatographic separation of *O*-glycan standards, comparison of PGC SPE and BOR SPE, design of the mixed-mode PGC-BOR SPE, and improved isomer separation (PDF)
Table S1: Overview of *O*-glycan released from PSM; Table S2: Overview of *O*-glycan released from the PaTu-S cell line (XLSX)
