# Supplementary material for: Comprehensive O-Glycan Analysis by Porous Graphitized Carbon Nanoliquid Chromatography–Mass Spectrometry
Source: Anal Chem. 2024 May 17;96(22):8942–8. doi: 10.1021/acs.analchem.3c05826 (PMC11154684; doi:10.1021/acs.analchem.3c05826)
Supplement: Supplementary file 1 — ac3c05826_si_001.pdf [file ac3c05826_si_001.pdf]

Comprehensive *O*-Glycan Analysis by Porous Graphitized Carbon Nano-Liquid Chromatography-Mass Spectrometry

Tao Zhang[1], Wenjun Wang[1], Manfred Wuhrer[1], Noortje de Haan[1,*]

1Center for Proteomics and Metabolomics, Leiden University Medical Center, P.O. Box 9600, 2300 RC Leiden, The Netherlands.

* Corresponding author: Noortje de Haan; E-mail: n.de_haan@lumc.nl

Content

Supporting Experimental Section

Supporting Figure S1: Annotated MS/MS for *O*-glycans released from PSM and PaTu-S cell line (Glycan 1-43). Glycan schemes were derived from GlycoWorkbench. Annotation was based on the presence of structural feature ions and common knowledge of known glycan synthetic pathways.

Supporting Figure S2: Chromatographic separation of *O*-glycan alditol standards Tn antigen, T antigen and sTn antigen.

Supporting Figure S3: Chromatographic separation of reduced $^{13}C_6$ isotope labeled GalNAc and deutero-reduced $^{13}C_6$ isotope labeled GlcNAc.

Supporting Figure S4: PGC nano-LC separation of two H1N1 isomers: Galβ1-3GalNAc-ol and Galβ1-3GlcNAc-ol.

Supporting Figure S5: PGC and BOR SPE of glycan standards, and an *O*-glycan mixture derived from BSM.

Supporting Figure S6: Presence of sialic acids decreases the BOR SPE recovery of *O*-glycans.

Supporting Figure S7: Design of the mixed-mode PGC-BOR SPE conditions and their recovery, using the *O*-glycan mixture derived from BSM as test sample.

Supporting Figure S8: PGC nano-LC separation of two H2N2 isomers released from PSM.

Supporting Figure S9: PGC nano-LC separation of two H2N2F1 isomers released from PSM.

Supporting Figure S10: PGC nano-LC separation of two H1N2 isomers released from the PaTu-S cell line.

Supporting Experimental Section

Materials and methods

Chemicals and materials

Ammonium hydroxide solution, glacial acetic acid, formic acid (FA), 2-propanol and potassium hydroxide (KOH) were purchased from Honeywell Fluka. Mucin from porcine stomach (PSM, Type III), mucin from bovine submaxillary glands (BSM, type I-S), Supel Carbon, 2.7 µm particle PGC HPLC column (5 cm x 2.1 mm; Supel Carbon analytical column), glycan standard T antigen (Galβ1-3GalNAc), Galβ1-4GlcNAc, Galβ1-3GlcNAc and ethanol were purchased from Merck (Darmstadt, Germany). Tn antigen GalNAc, GlcNAc, ammonium bicarbonate (ABC), sodium borohydride (NaBH4), sodium borodeuteride (NaBD4), cation-exchange resin Dowex (50W-X8), fetuin from fetal bovine serum (FBS), DL-dithiothreitol (DTT) and hydrochloric acid (HCl) were obtained from Sigma-Aldrich (Steinheim, Germany). sTn MUC1 was kindly supplied by Dr. Yoshiki Narimatsu (University of Copenhagen).[1] Isotope $^{13}C_6$ labeled GalNAc, isotope $^{13}C_6$ labeled GlcNAc were purchased from Cambridge Isotope Laboraories, Inc. (MA, USA). Peptide N-glycosidase F (PNGase F) was obtained from Roche Diagnostics (Mannheim, Germany). 8 M guanidine hydrochloride (GuHCl) was obtained from Thermo Fisher Scientific (Waltham, MA). MultiScreen HTS 96-well plates (hydrophobic Immobilon-P PVDF membrane) were obtained from Millipore (Amsterdam, the Netherlands) and 96-well PP filter plate from Orochem Technologies (Naperville, IL). Bulk sorbent Carbograph was obtained from Grace Discovery sciences (Columbia, SC). Immobilized boronic acid resin was obtained from Pierce (Product number: 20244, Thermo Fisher Scientific). Acetonitrile and methanol were purchased from Actu-All Chemicals (Oss, the Netherlands). Ultrapure water was generated from an ELGA Labwater system (Ede, the Netherlands).

Cell culture and pellet preparation

The PaTu-8988S (PaTu-S) cell line was obtained from DSMZ culture bank (Braunschweig, Germany). PaTu-S cells were cultured in Dulbecco's modified Eagle medium (DMEM) with 10% fetal bovine serum (FBS) and 100U/mL penicillin-streptomycin. This cell line was frequently tested for the absence of mycoplasma contamination and authenticated by short tandem repeat (STR) profiling. PaTu-S cells were harvested by trypsin followed by 3 x washing using PBS.

Glycan standard reduction and desalting

Reduction and desalting of glycan standards GalNAc, GlcNAc, T antigen Galβ1-3GalNAc, Galβ1-3GlcNAc, Galβ1-4GlcNAc, and $^{13}C_6$ isotope labeled GalNAc were performed as described previously.[2] In addition, $^{13}C_6$ isotope labeled GlcNAc was deutero-reduced using a 200 µL sodium borodeuteride (3 mg/mL) solution in 2M aqueous ammonium hydroxide at room temperature for 24 h, and the reaction was terminated by addition of 2 µL glacial acetic acid. The sTn alditol standard was released from 10 µg sTn MUC1 using a 96-well plate sample preparation method performed as previously described.[2]

O-glycan release and desalting

O-glycan alditols released from BSM, PSM and PaTu-S cells were prepared using a 96-well plate sample preparation method performed as previously described.[2] In brief, 20 µg glycoprotein and lysates from 5 x 10^5 cells were applied to the hydrophobic Immobilon-P PVDF membrane in a 96-well plate format. Protein denaturation was achieved by applying 75 µL denaturation mix (72.5 µL 8 M GuHCl and 2.5 µL 200 mM DTT) in each well, followed by removal of the unbound material by centrifugation. After removal of *N*-glycans using PNGase F, the *O*-glycans were released from the same PVDF membrane-immobilized sample via reductive β-elimination at 50 °C for 16 h. The *O*-glycan alditols were collected by centrifuge and concentrated to approximately 30 µL under vacuum in a SpeedVac concentrator at 35 °C. Subsequently, 3 µL of glacial acetic acid was added to quench the reaction, followed by cation exchange desalting in a high-throughput manner. The desalted *O*-glycan alditols were re-suspended in 10 µL of water prior to PGC-BOR SPE.

Optimization of PGC-BOR SPE for O-glycan purification

O-glycan purification using combined PGC-BOR SPE was performed in a 96-well filter plate. For the optimized protocol, 10 µL of the immobilized boronic acid resin slurry (50%) was added to each well in the filter plate and packed by centrifuging at 200 x g for 1 min. Next, 90 µL of bulk sorbent Carbograph slurry in 50% (v/v) methanol

was packed to the same well by centrifugation under the same conditions. The columns were preconditioned by 1 x 100 µL of 100 mM FA, 2 x 100 µL of 80% ACN, and 1 x 100 µL of 200 mM ABC (pH8.8), each time followed by centrifuging at 500 x g for 1 min. The samples were mixed with 10 µL of 400 mM ABC (pH 8.8), loaded onto the columns and washed 2 x with 100 µL of 200 mM ABC (pH 8.8) and 1 x with 100 µL of water, each step followed by centrifugation at 500 x g for 1min. Next, the *O*-glycan alditols were sequentially eluted by 1 x 100 µL of 100 mM FA, 1 x 100 µL of 60% ACN with 0.1% TFA, and 1 x 100 µL of 25 mM HCl, centrifuging 2 min at 800 x g . The eluants were combined and dried in a SpeedVac concentrator at 35 °C for 3 h. Prior to PGC nano-LC-MS/MS analysis, the samples were re-suspended in 10 µL of water.

For different packing format of PGC-BOR SPE, 50 µL of the immobilized boronic acid resin slurry (50%) and 50 µL of bulk sorbent Carbograph slurry in 50% (v/v) methanol were packed to each well in the filter plate with opposite order. The column equilibration, sample loading, washing and elution steps were performed following the procedure above.

PGC SPE purification of O-glycan samples

Purification of *O*-glycan alditols using PGC-SPE was performed as described previously. [2] In brief, PGC-SPE columns were prepared by packing 100 µL of bulk Carbograph slurry (50%) in methanol to a 96-well plate. The columns were preconditioned by 3 x 100 µL of 80% ACN in water with 0.1% TFA by centrifugation at 500 x g for 1min. *O*-glycan samples were re-suspended in 40 µL water with 0.1% TFA. After loading the sample, the columns were washed 3 x with 100 µL of water with 0.1% TFA, followed by *O*-glycan elution by 3 x 40 µL of 60% ACN in water with 0.1% TFA by centrifugation at 500 x g for 1 min. The eluents were collected and dried in a SpeedVac concentrator at 35 °C for 3 h and re-suspended in 10 µL of water prior to PGC nano-LC-MS/MS analysis.

BOR SPE purification of O-glycan samples

Purification of *O*-glycan alditols using BOR-SPE was performed as described previously, [3] adapting the procedure to a 96-well filter plate format. In brief, 50 µL of the immobilized boronic acid resin slurry was added to each well in the filter plate and packed by centrifuging at 1000 rpm. The columns were preconditioned by 1 x 100 µL of 100 mM FA, followed by 2 x 100 µL of 200 mM ABC (pH8.8) each time removed by centrifuging at 500 x g. The samples were mixed with 10 µL of 400 mM ABC (pH8.8) and loaded onto the columns and washed 2 x with 100 µL of 200 mM ABC (pH8.8) by centrifugation at 500 x g. Next, the *O*-glycan alditols were eluted by 2 x 100 µL of 100 mM

FA and 1 x 100 µL of 25 mM HCl each time centrifuged at 800 x g. The eluents were collected and dried in a SpeedVac concentrator at 35 °C for 3 h and re-suspended in 10 µL of water prior to PGC nano-LC-MS/MS analysis.

The optimization conditions for the nanoLC

Analysis was performed using a PGC nano-LC Ultimate 3000 UHPLC system (Thermo Fisher Scientific) coupled to an amaZon ETD speed ion trap (Bruker Daltonics, Bremen, Germany). Trap columns with 320 µm inner diameter and different length (4, 6 and 8 cm) and a separation column (75 µm x 15 cm) were home-packed with 2.7 µm PGC particles derived from the Supel Carbon analytical column. The LC system was coupled to an amaZon ETD speed ESI ion trap MS using the CaptiveSpray™ source (Bruker Daltonics), and used in negative-ionization mode. In the optimized method, mobile phase A consisted of 10 mM ABC, while mobile phase B was 60% (v/v) acetonitrile/10 mM ABC. To analyze *O*-glycans, 1 µL injections were performed, and trapping was achieved on the trap column using a 6 µL/min loading flow in 5% buffer B for 3 min. Separation was achieved with a multi-step gradient of B: 5-5% in 20 min and 5-69% over 80 min followed by a 10 min wash step using 95% of B at a flow of rate of 0.6 µL/min. The column was held at a constant temperature of 30 °C.

Measurements were performed on an Ultimate 3000 UHPLC system (Thermo Fisher Scientific) equipped with a home-packed PGC trap column (2.7 µm, 320 µm x 80 mm) and a home-packed PGC nano-column (2.7 µm, 75 µm x 150 mm) coupled to an amaZon ETD speed ion trap (Bruker Daltonics). Mobile phase A consisted of 10 mM ABC, while mobile phase B was 60% (v/v) acetonitrile/10 mM ABC. To analyze *O*-glycans, 1 µL injections were performed, and trapping was achieved on the trap column using a 6 µL/min loading flow in 5% buffer B for 3 min. Separation was achieved with a multi-step gradient of B: 5-5% in 20 min and 5-69% over 80 min followed by a 10 min wash step using 95% of B at a flow of rate of 0.6 µL/min. The column was held at a constant temperature of 30 °C.

Ionization was achieved using the nanoBooster source (Bruker Daltonics) with a capillary voltage of 1000 V applied and a dry gas temperature of 280 °C at 5 L/min and isopropanol enriched nitrogen at 3 psi. MS spectra were acquired within an *m/z* range of 220–1800 in enhanced mode using negative ion mode, smart parameter setting was set to *m/z* 800. Ion charge control (ICC) was set to 40,000 and maximum acquisition time was kept at 200 ms. MS/MS spectra were generated by collision-induced dissociation on the top 3 highest intensity peaks in each MS1 spectrum, applying an isolation width of *m/z* 3. The fragmentation cut-off was set to 27% with 100% fragmentation amplitude using the Enhanced SmartFrag option (30-120% in 32 ms) and ICC was set to 150,000.

Structures of detected glycans were studied by MS/MS in negative mode. Glycan structures were assigned on the basis of the known MS/MS fragmentation patterns in negative-ion mode,[4] elution order, and general glycobiological knowledge, with the help of Glycoworkbench[5] and Glycomod[6] software. Relative quantification of individual glycans was performed by normalizing the total peak area of all glycans within one sample to 100%.

References

(1) Nason, R.; Büll, C.; Konstantinidi, A.; Sun, L. B.; Ye, Z. L.; Halim, A.; Du, W. J.; Sorensen, D. M.; Durbesson, F.; Furukawa, S.; et al. Display of the human mucinome with defined O-glycans by gene engineered cells. *Nat Commun* **2021**, *12* (1).
(2) Zhang, T.; Madunic, K.; Holst, S.; Zhang, J.; Jin, C. S.; ten Dijke, P.; Karlsson, N. G.; Stavenhagen, K.; Wuhrer, M. Development of a 96-well plate sample preparation method for integrated N- and O-glycomics using porous graphitized carbon liquid chromatography-mass spectrometry. *Mol Omics* **2020**, *16* (4), 355-363.
(3) Zhang, Q.; Schepmoes, A. A.; Brock, J. W. C.; Wu, S.; Moore, R. J.; Purvine, S. O.; Baynes, J. W.; Smith, R. D.; Metz, T. O. Improved Methods for the Enrichment and Analysis of Glycated Peptides. *Anal Chem* **2008**, *80* (24), 9822-9829.
(4) Karlsson, N. G.; Wilson, N. L.; Wirth, H. J.; Dawes, P.; Joshi, H.; Packer, N. H. Negative ion graphitised carbon nano-liquid chromatography/mass spectrometry increases sensitivity for glycoprotein oligosaccharide analysis. *Rapid Commun Mass Sp* **2004**, *18* (19), 2282-2292.
(5) Ceroni, A.; Maass, K.; Geyer, H.; Geyer, R.; Dell, A.; Haslam, S. M. GlycoWorkbench: A tool for the computer-assisted annotation of mass spectra of Glycans. *J Proteome Res* **2008**, *7* (4), 1650-1659.
(6) Cooper, C. A.; Gasteiger, E.; Packer, N. H. GlycoMod - A software tool for determining glycosylation compositions from mass spectrometric data. *Proteomics* **2001**, *1* (2), 340-349.

Supporting Figure S1-1: Annotated MS/MS for *O*-glycans released from PSM and PaTu-S cell line (Glycan 1).

Glycan 1

Tn antigen
N1a

Charge observed: 1-
Theoretical ion: *m/z* 222.10
Observed ion: *m/z* 222.05
Mass deviation: *m/z* 0.05
Structure confirmed by standard

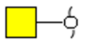

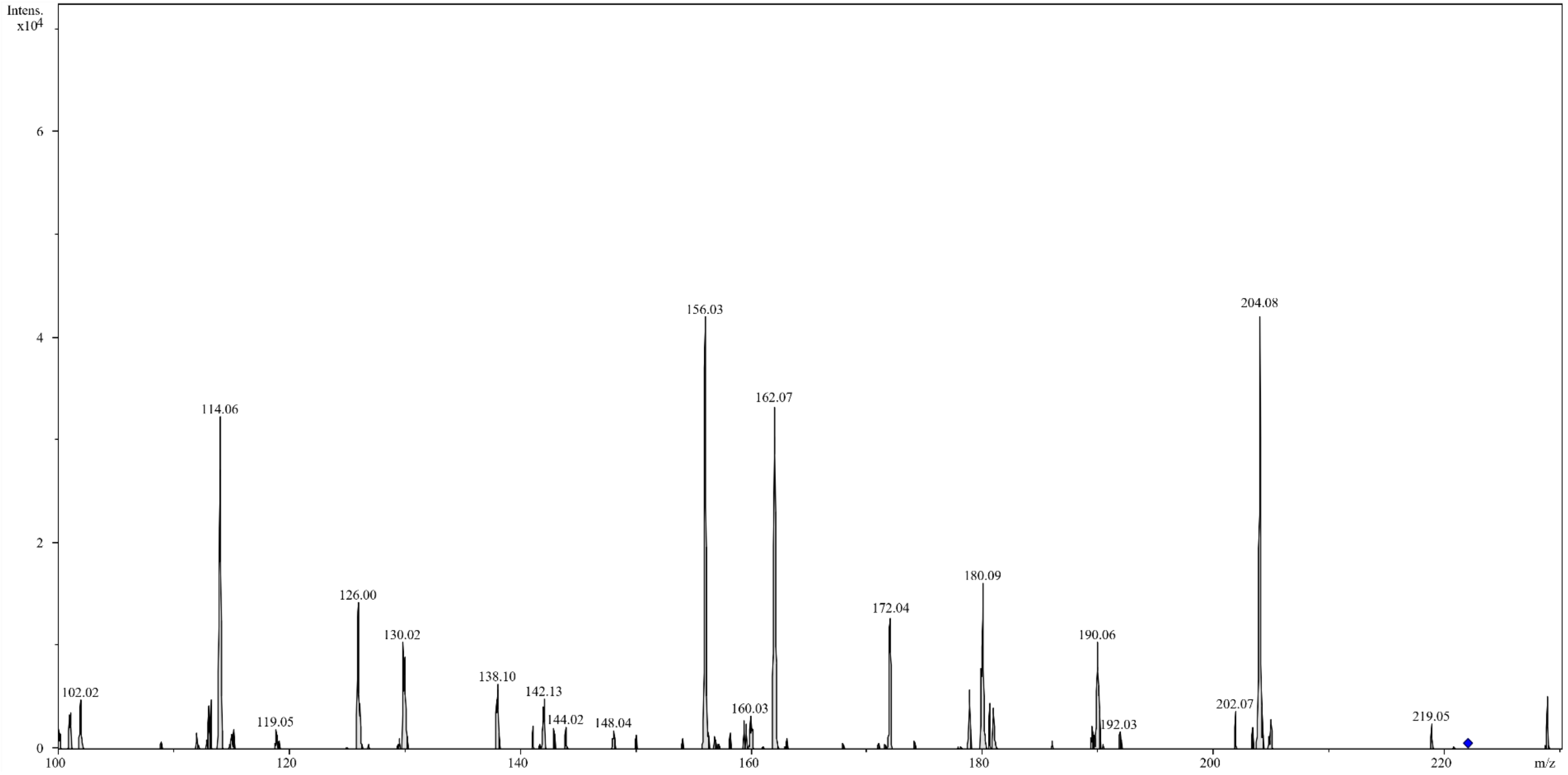

Supporting Figure S1-2: Annotated MS/MS for *O*-glycans released from PSM and PaTu-S cell line (Glycan 2).

Glycan 2

GlcNAc
N1b

Charge observed: 1-
Theoretical ion: *m/z* 222.10
Observed ion: *m/z* 222.04
Mass deviation: *m/z* 0.06
Structure confirmed by standard

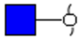

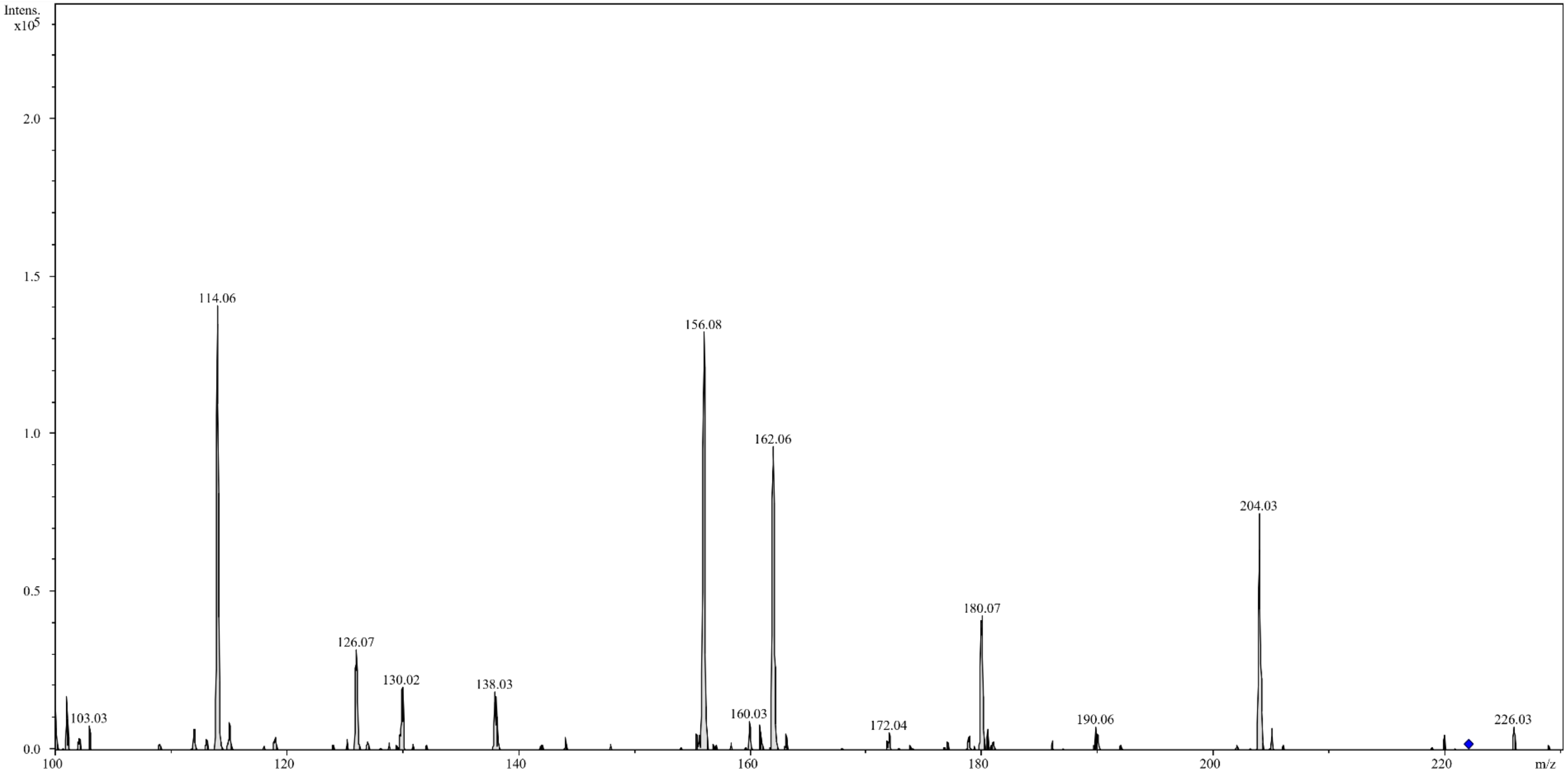

Supporting Figure S1-3: Annotated MS/MS for *O*-glycans released from PSM and PaTu-S cell line (Glycan 3).

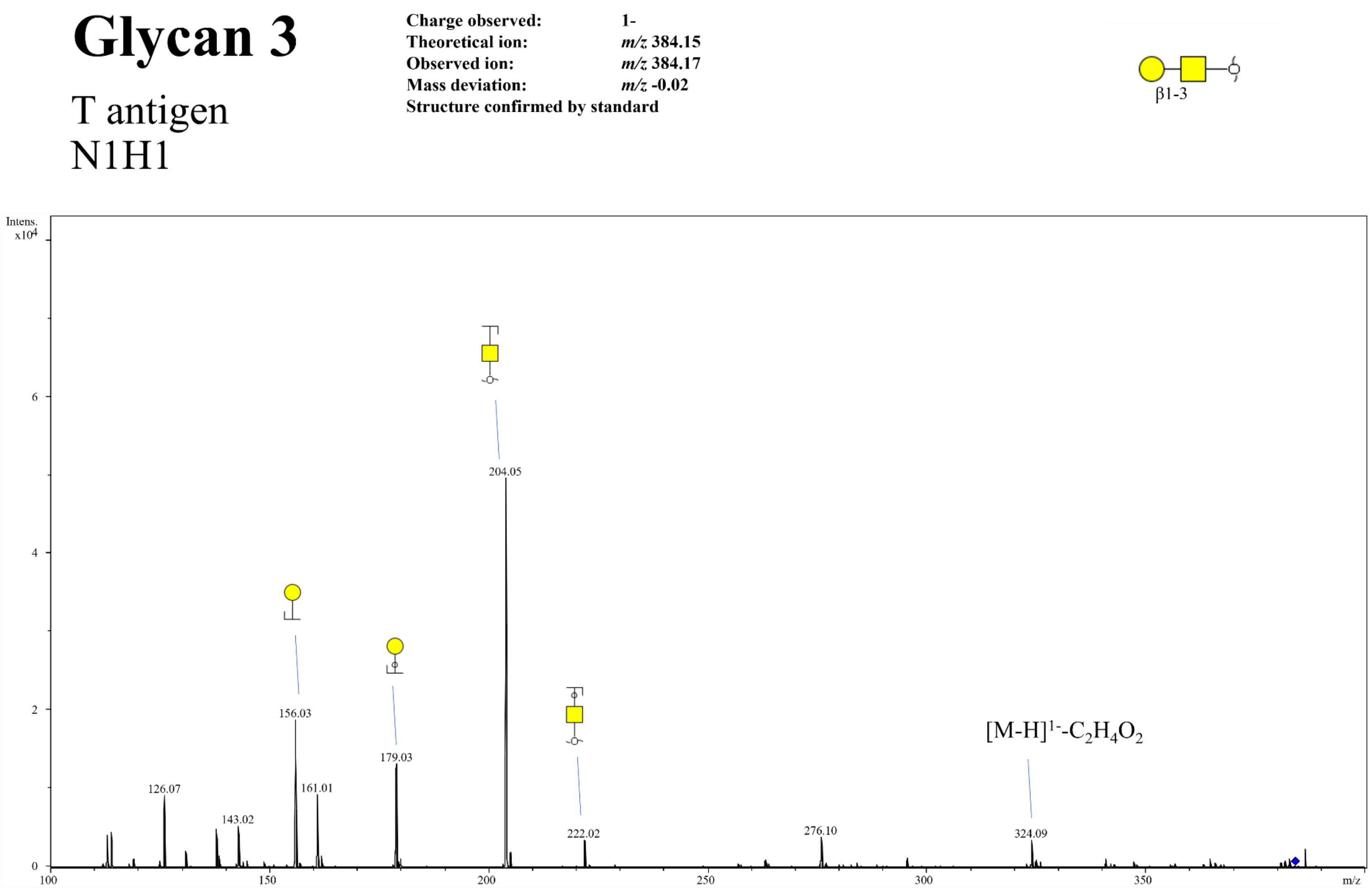

Supporting Figure S1-4: Annotated MS/MS for *O*-glycans released from PSM and PaTu-S cell line (Glycan 4).

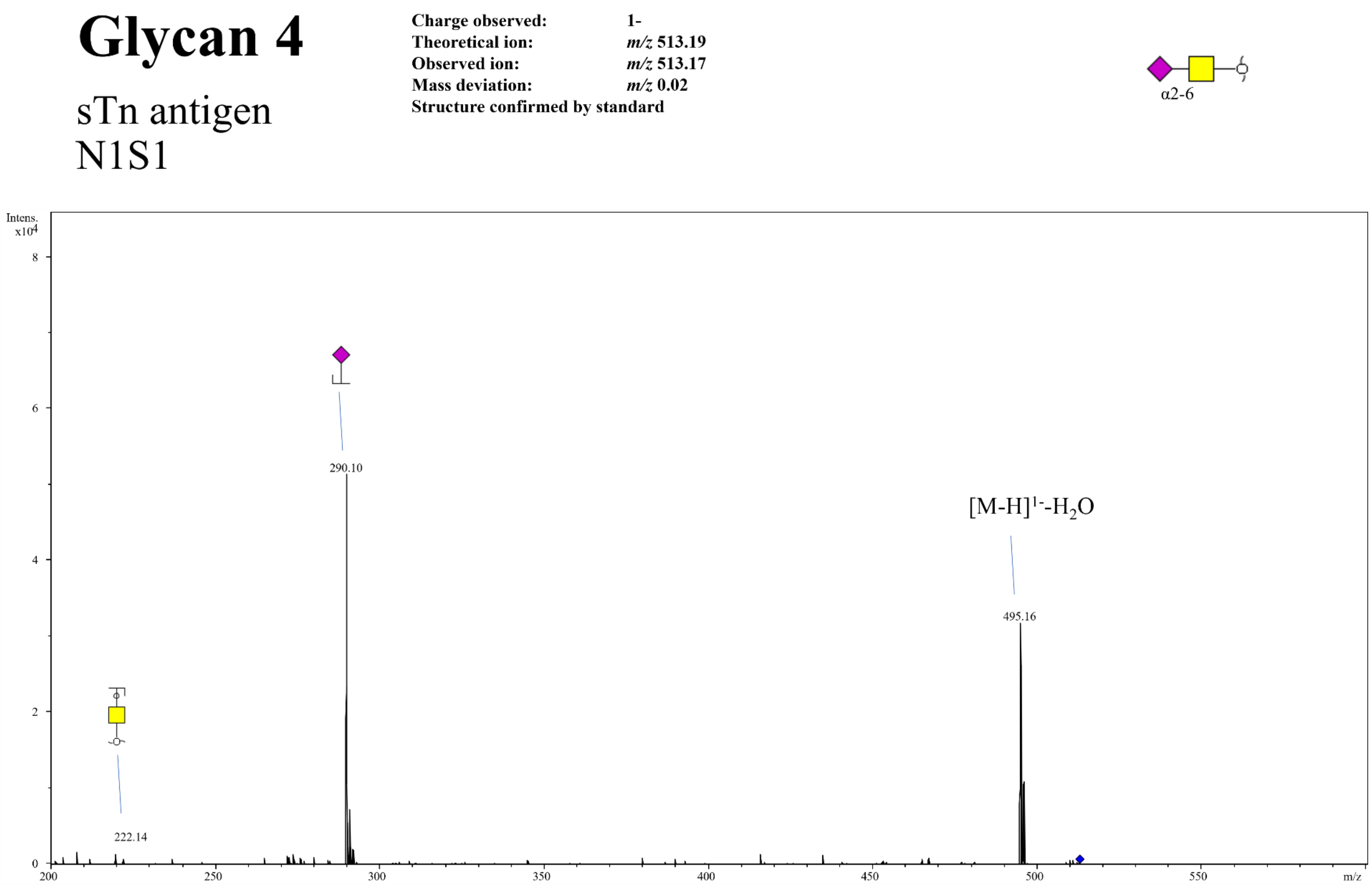

Supporting Figure S1-5: Annotated MS/MS for *O*-glycans released from PSM and PaTu-S cell line (Glycan 5).

Glycan 5

**Charge observed:**	**1-**
**Theoretical ion:**	***m/z*** **587.23**
**Observed ion:**	***m/z*** **587.19**
**Mass deviation:**	***m/z*** **0.04**

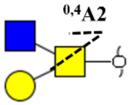

N2H1a

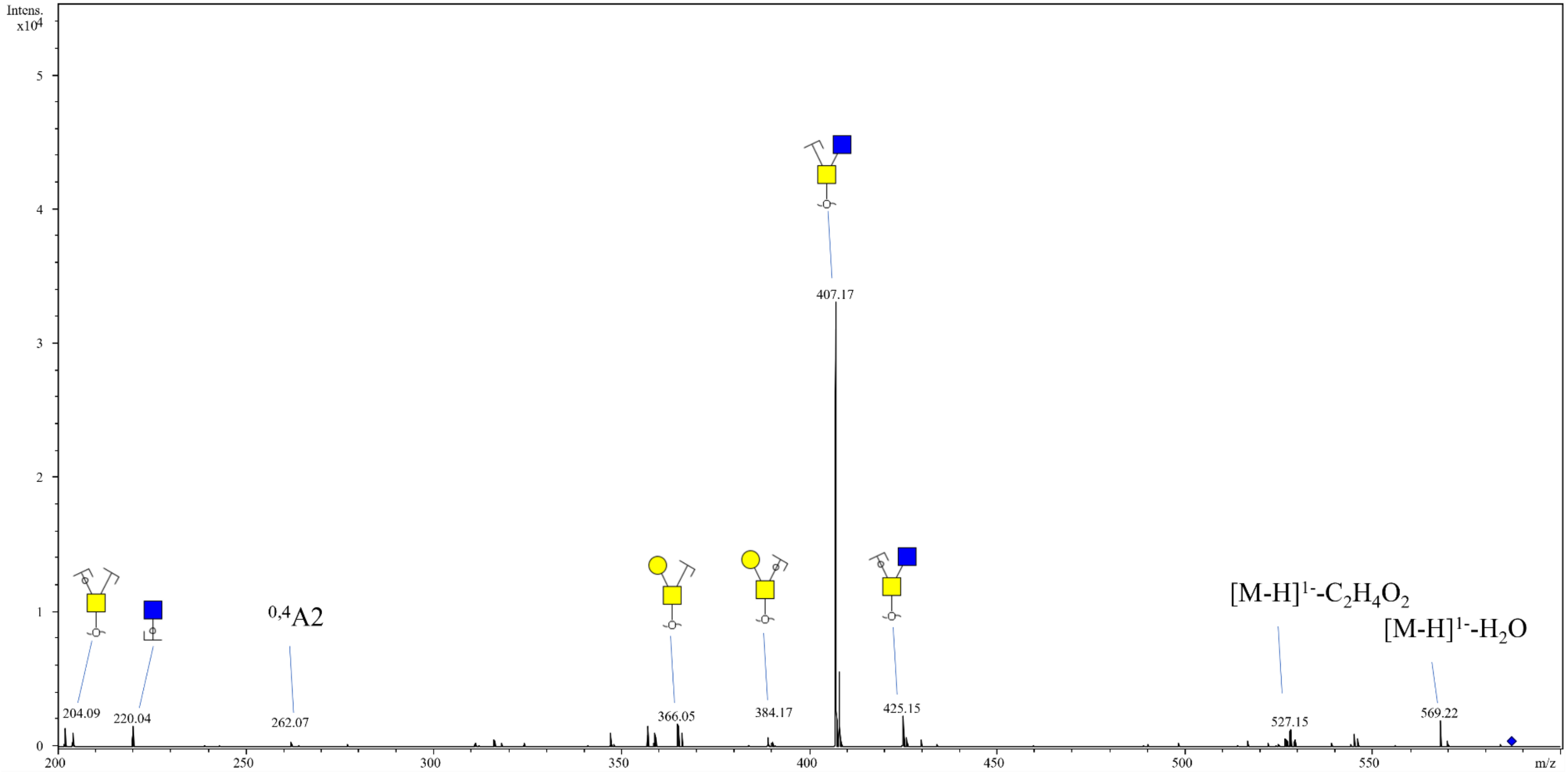

Supporting Figure S1-6: Annotated MS/MS for *O*-glycans released from PSM and PaTu-S cell line (Glycan 6).

Glycan 6

Charge observed: 1-
Theoretical ion: *m/z* 587.23
Observed ion: *m/z* 587.22
Mass deviation: *m/z* 0.01

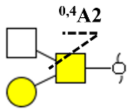

N2H1b

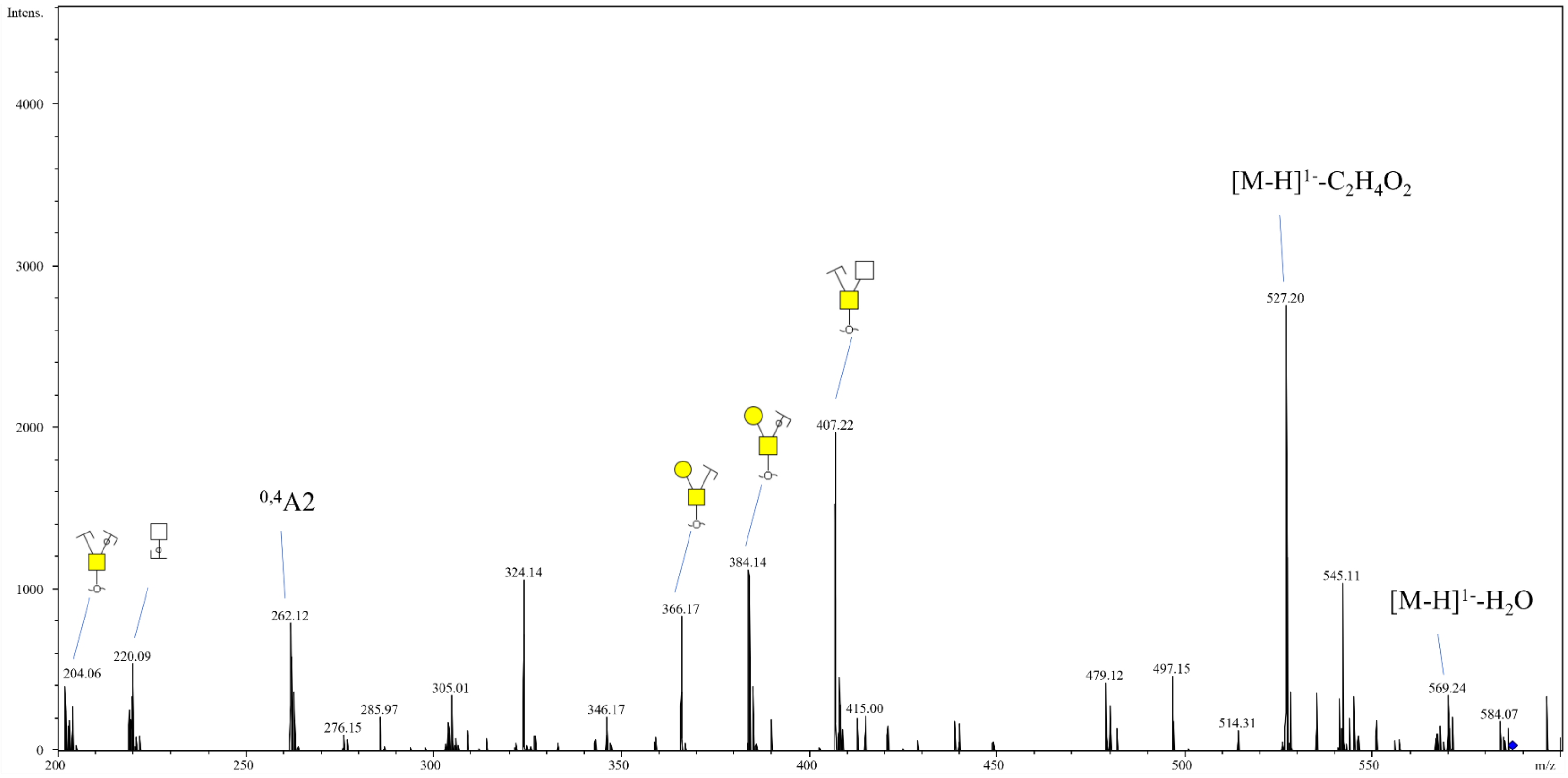

Supporting Figure S1-7: Annotated MS/MS for *O*-glycans released from PSM and PaTu-S cell line (Glycan 7).

Glycan 7

Charge observed: 1-
Theoretical ion: *m/z* 675.25
Observed ion: *m/z* 675.23
Mass deviation: *m/z* 0.02

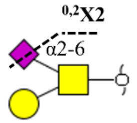

N1H1S1a

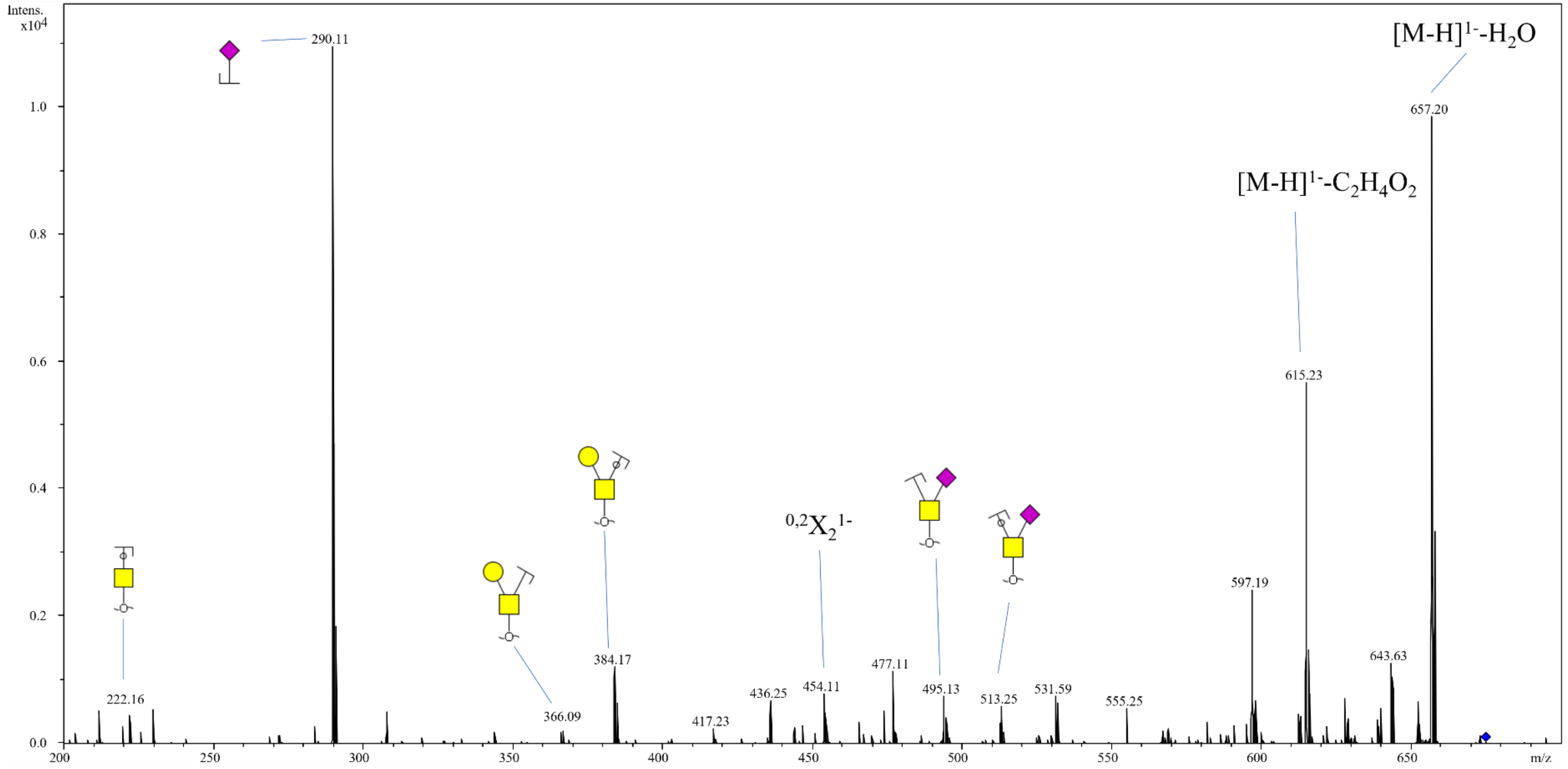

Supporting Figure S1-8: Annotated MS/MS for *O*-glycans released from PSM and PaTu-S cell line (Glycan 8).

Glycan 8

Charge observed: 1-
Theoretical ion: *m/z* 675.25
Observed ion: *m/z* 675.22
Mass deviation: *m/z* 0.03

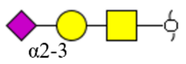

N1H1S1b

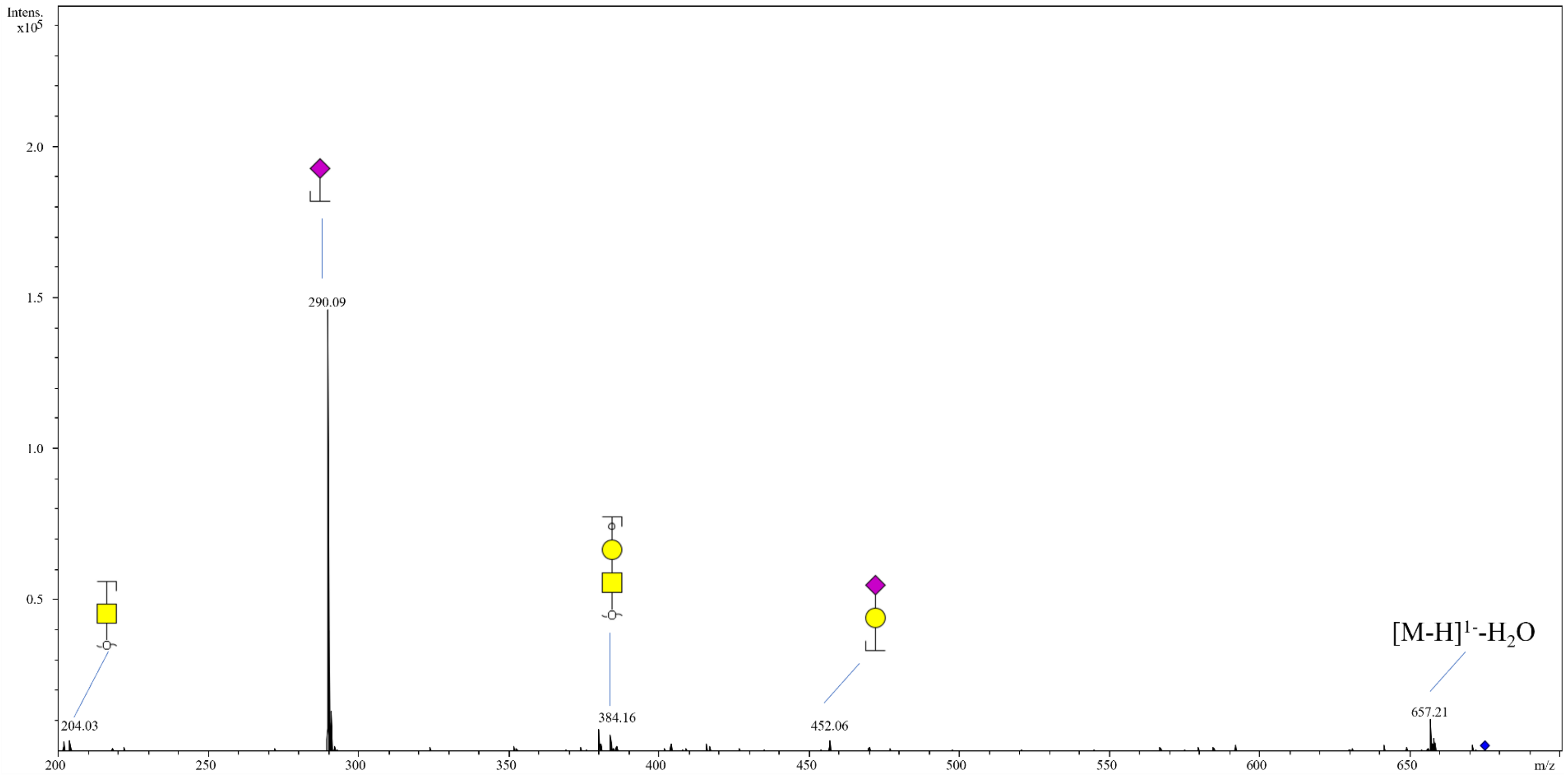

Supporting Figure S1-9: Annotated MS/MS for *O*-glycans released from PSM and PaTu-S cell line (Glycan 9).

Glycan 9

Charge observed: 1-
Theoretical ion: ***m/z*** **749.28**
Observed ion: ***m/z*** **749.28**
Mass deviation: ***m/z*** **0.00**

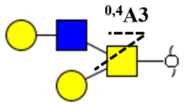

N2H2a

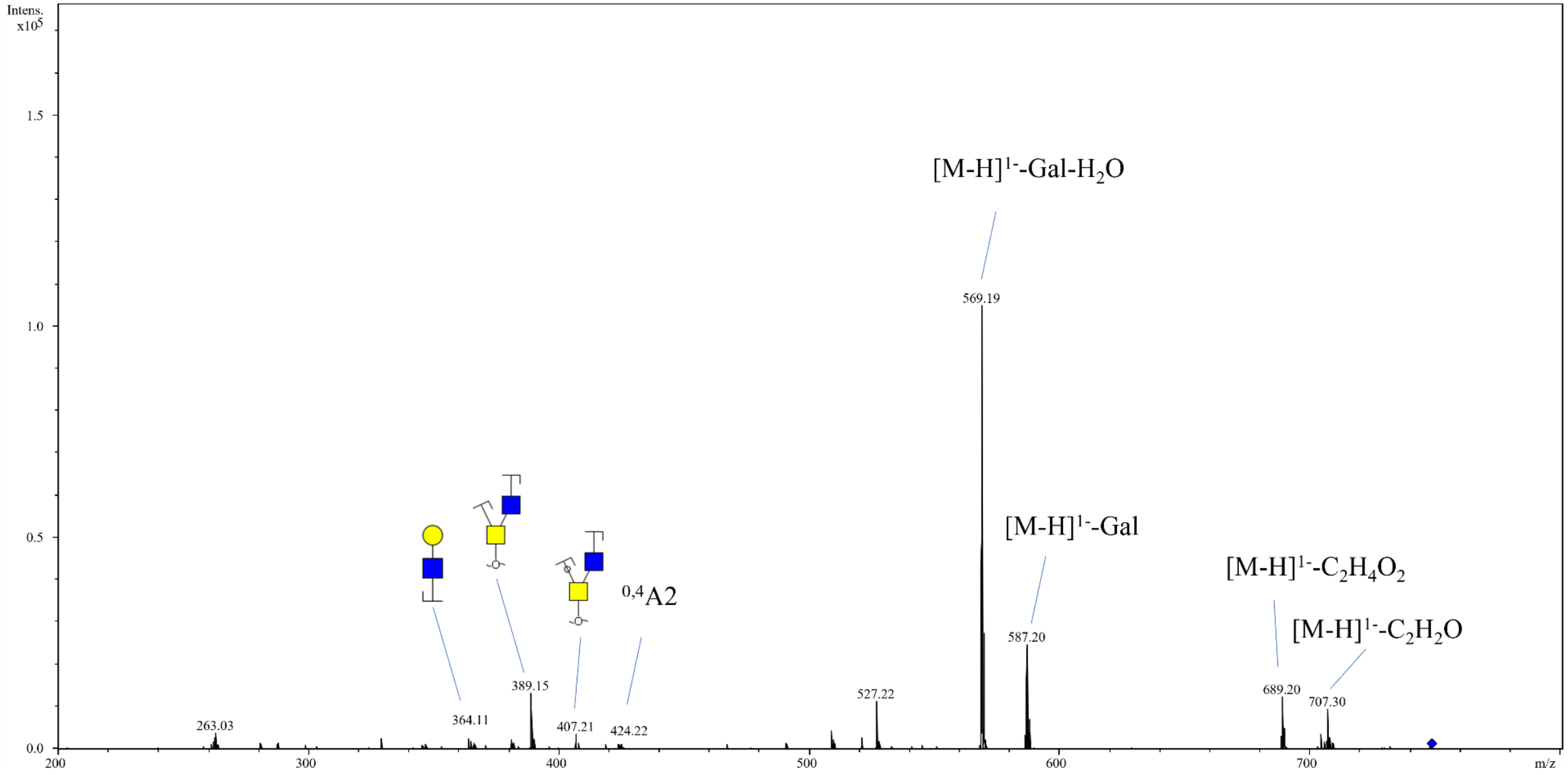

Supporting Figure S1-10: Annotated MS/MS for *O*-glycans released from PSM and PaTu-S cell line (Glycan 10).

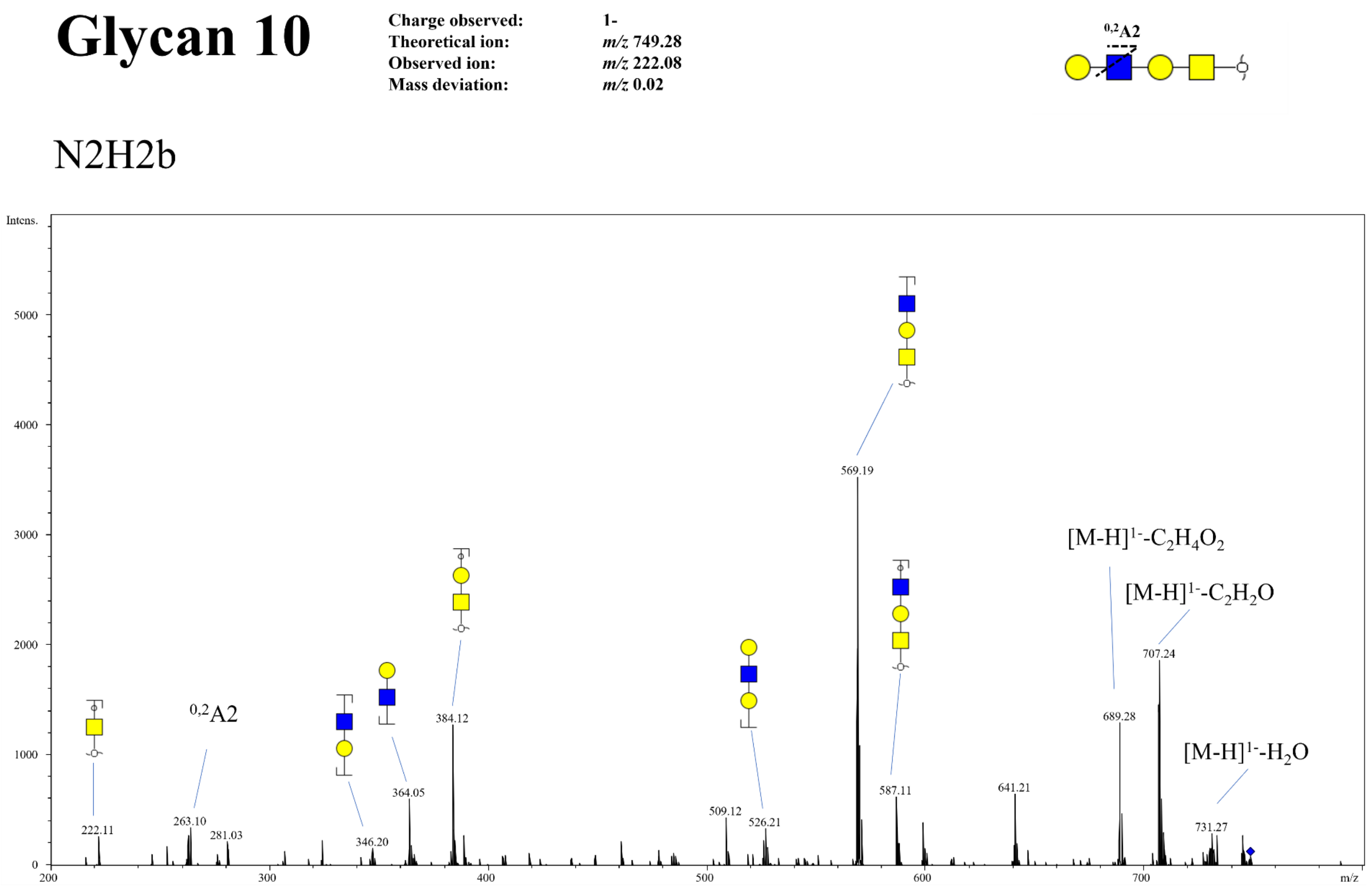

Supporting Figure S1-11: Annotated MS/MS for *O*-glycans released from PSM and PaTu-S cell line (Glycan 11).

Glycan 11

**Charge observed:**	**1-**
**Theoretical ion:**	***m/z* 878.33**
**Observed ion:**	***m/z* 878.31**
**Mass deviation:**	***m/z* 0.02**

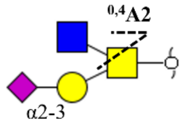

N2H1S1

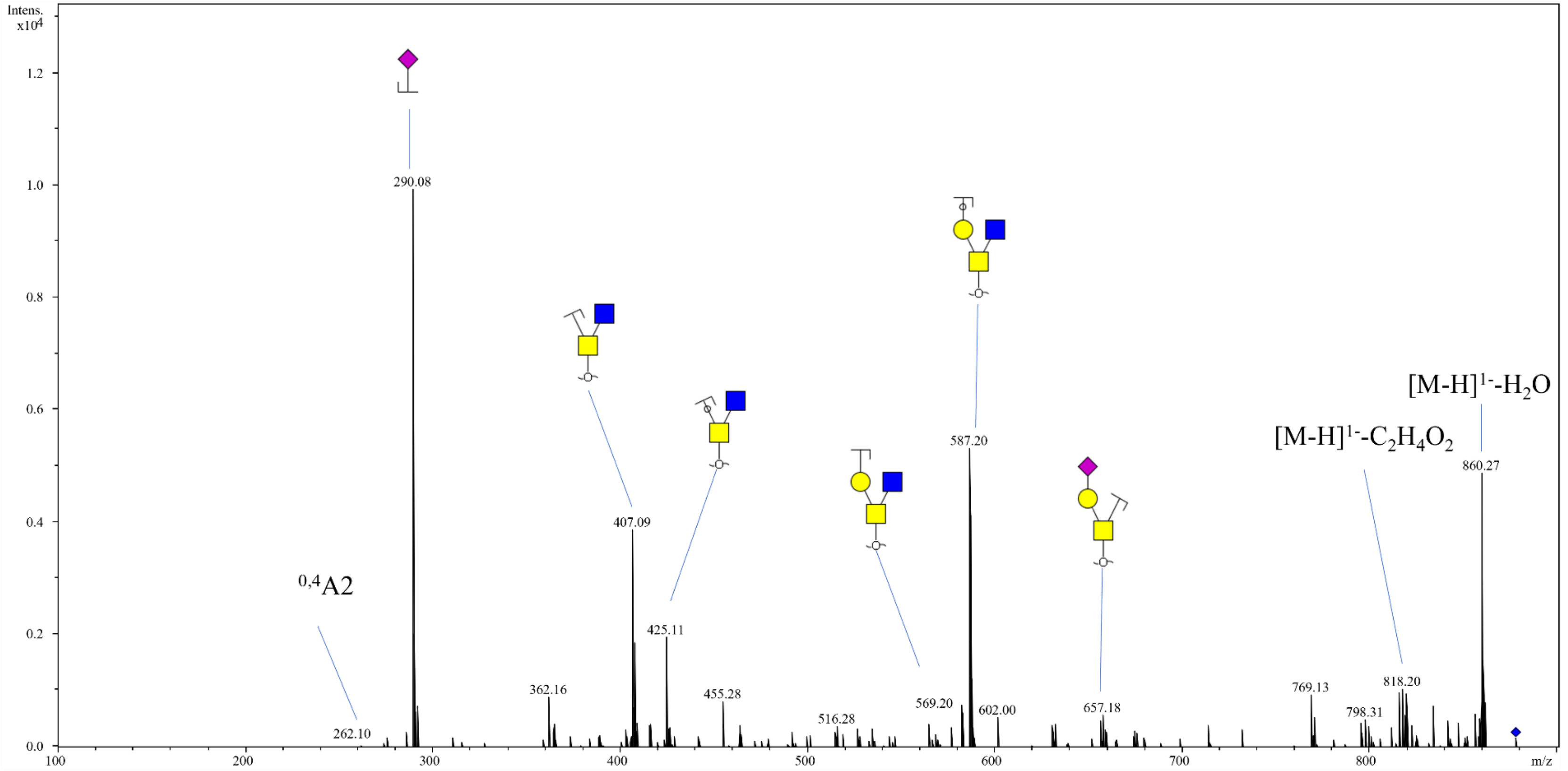

Supporting Figure S1-12: Annotated MS/MS for *O*-glycans released from PSM and PaTu-S cell line (Glycan 12).

Glycan 12

Charge observed: 1-
Theoretical ion: *m/z* 895.34
Observed ion: *m/z* 895.33
Mass deviation: *m/z* 0.01

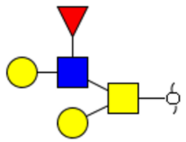

N2H2F1a

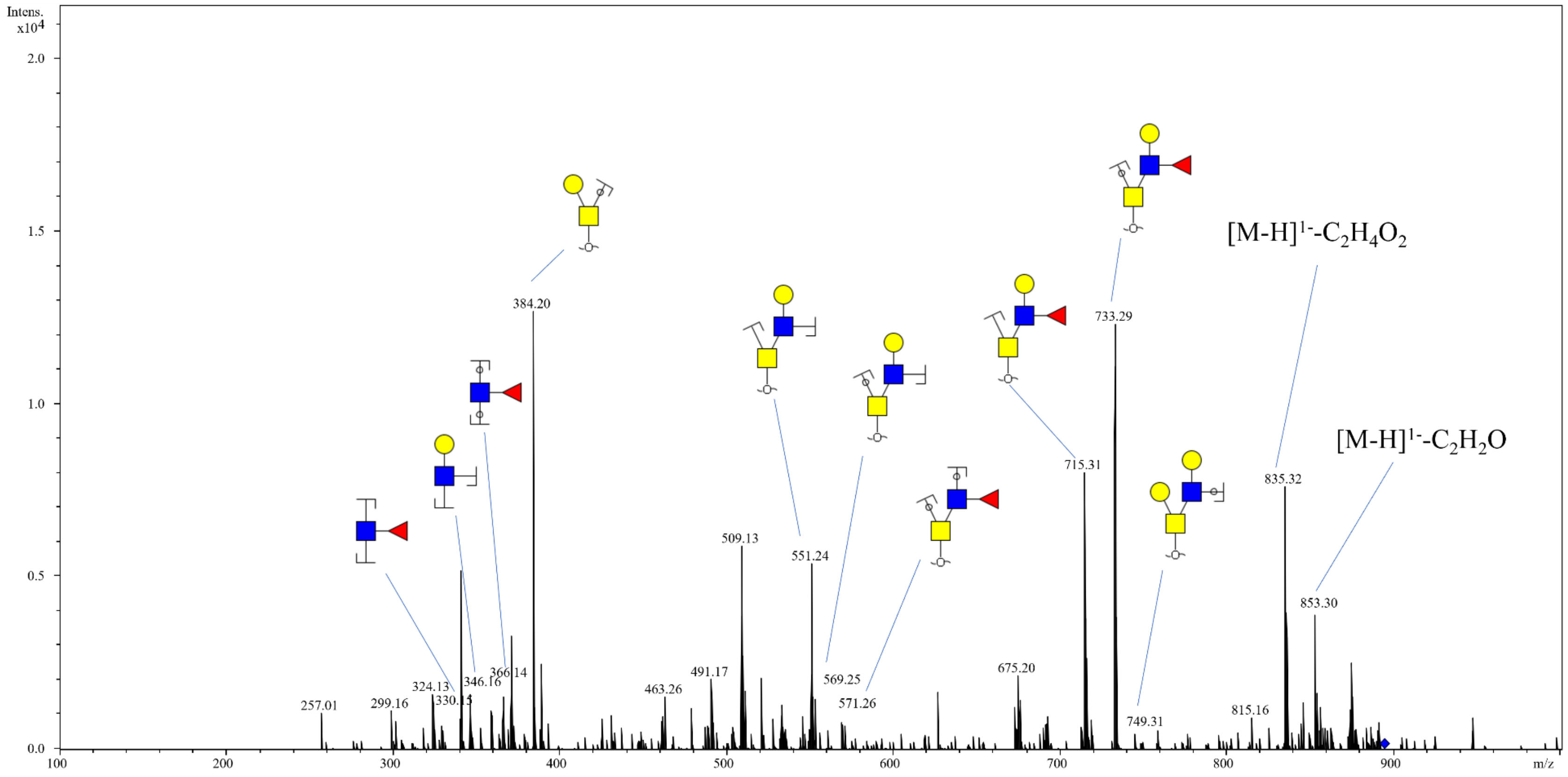

Supporting Figure S1-13: Annotated MS/MS for *O*-glycans released from PSM and PaTu-S cell line (Glycan 13).

Glycan 13

**Charge observed:**	**1-**
**Theoretical ion:**	***m/z* 895.34**
**Observed ion:**	***m/z* 895.30**
**Mass deviation:**	***m/z* 0.04**

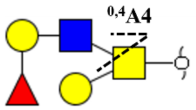

N2H2F1b

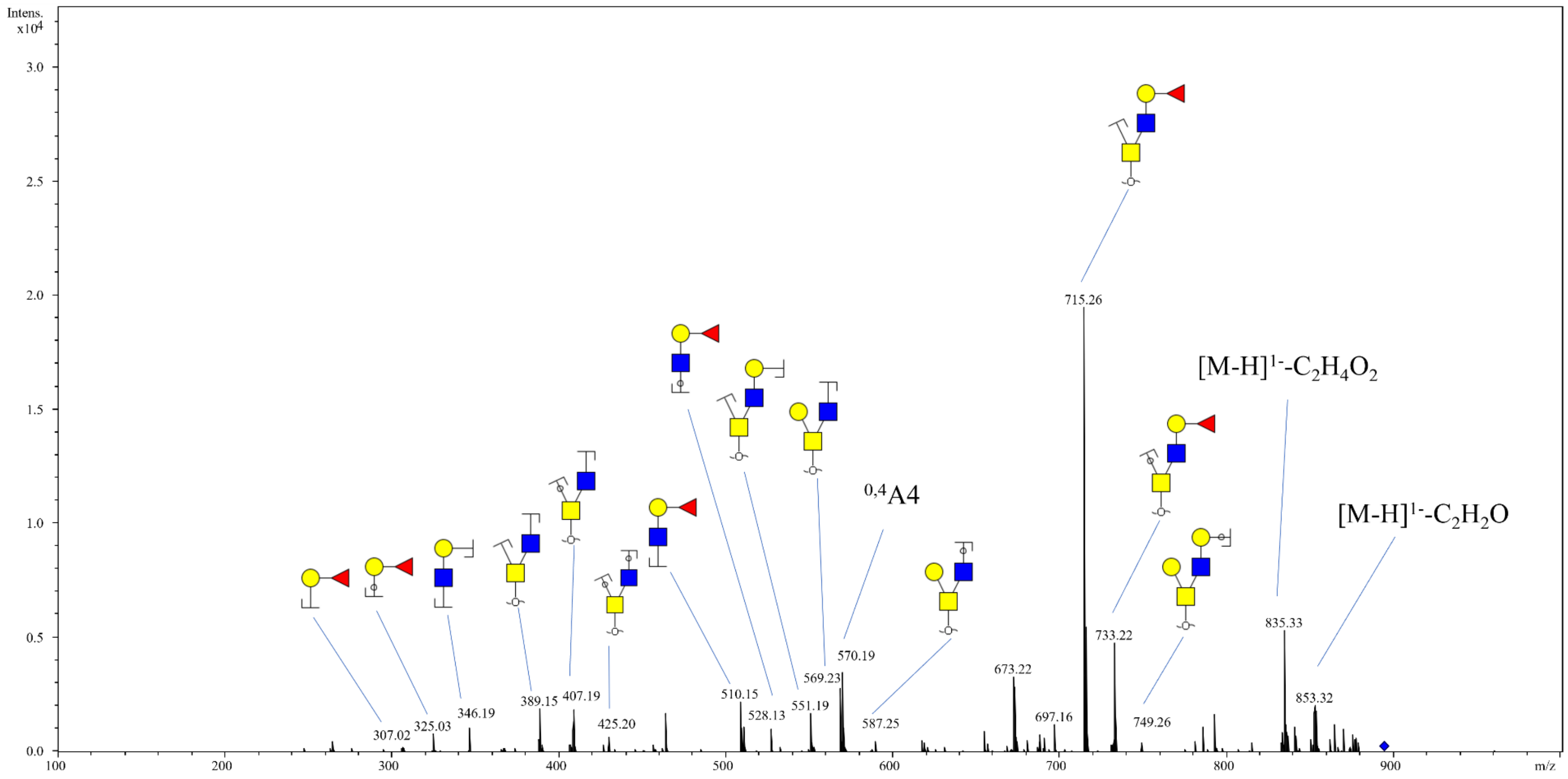

Supporting Figure S1-14: Annotated MS/MS for *O*-glycans released from PSM and PaTu-S cell line (Glycan 14).

Glycan 14

Charge observed: 1-
Theoretical ion: *m/z* 895.34
Observed ion: *m/z* 895.33
Mass deviation: *m/z* 0.01

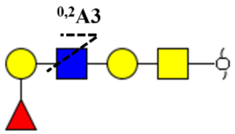

N2H2F1c

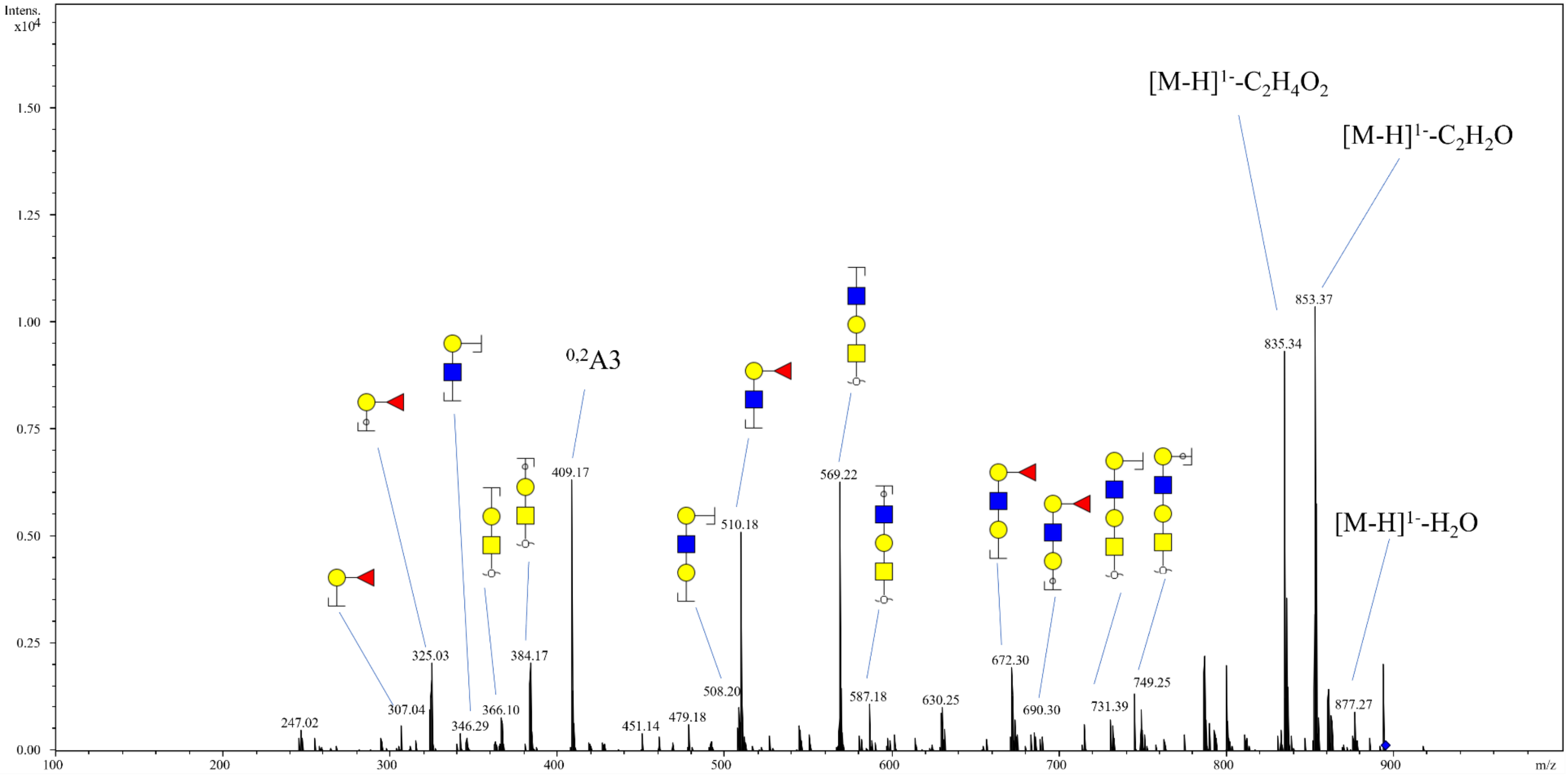

Supporting Figure S1-15: Annotated MS/MS for *O*-glycans released from PSM and PaTu-S cell line (Glycan 15).

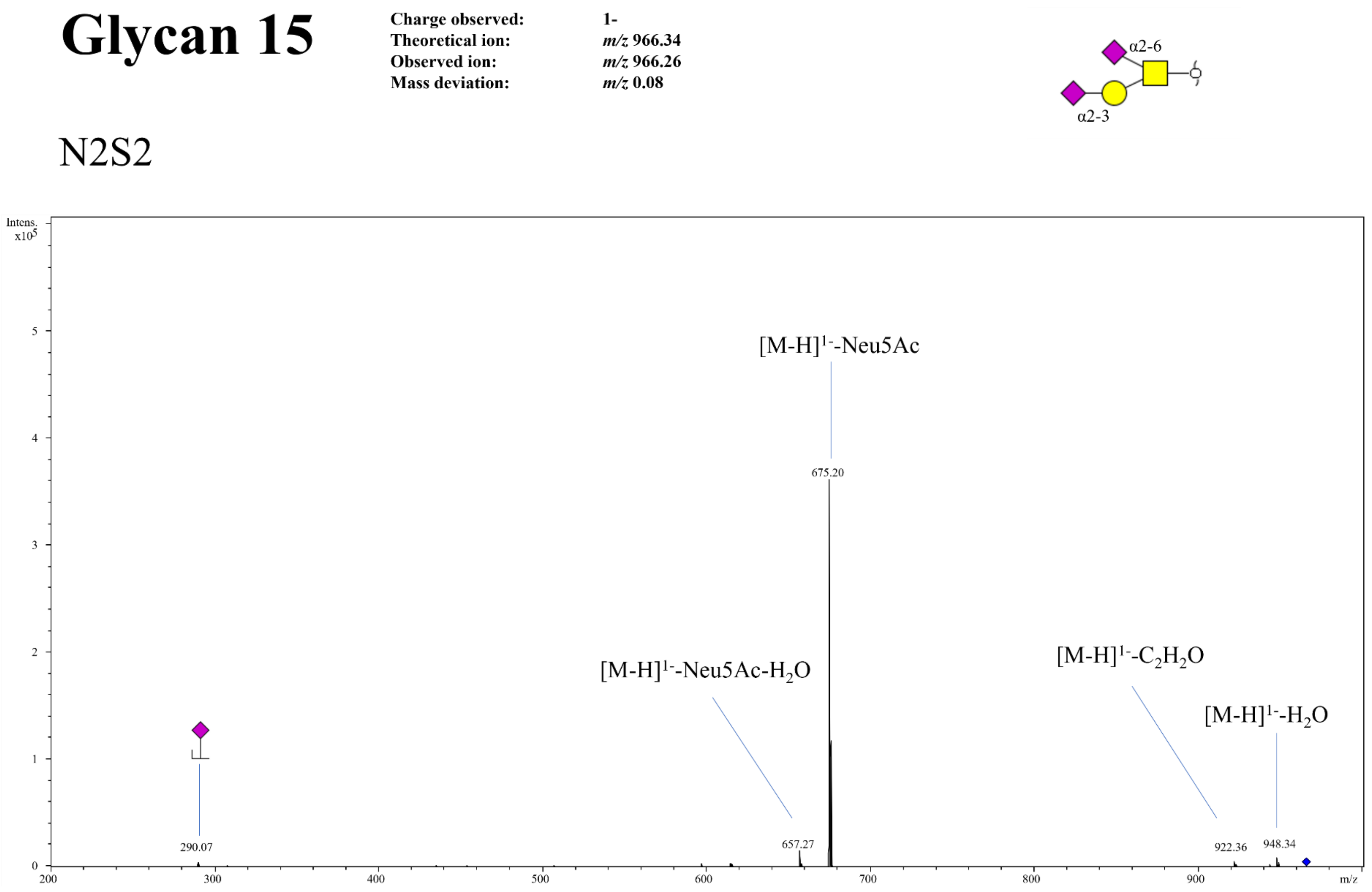

Supporting Figure S1-16: Annotated MS/MS for *O*-glycans released from PSM and PaTu-S cell line (Glycan 16).

Glycan 16

Charge observed: 1-
Theoretical ion: *m/z* 993.39
Observed ion: *m/z* 993.36
Mass deviation: *m/z* 0.03

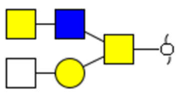

N4H1

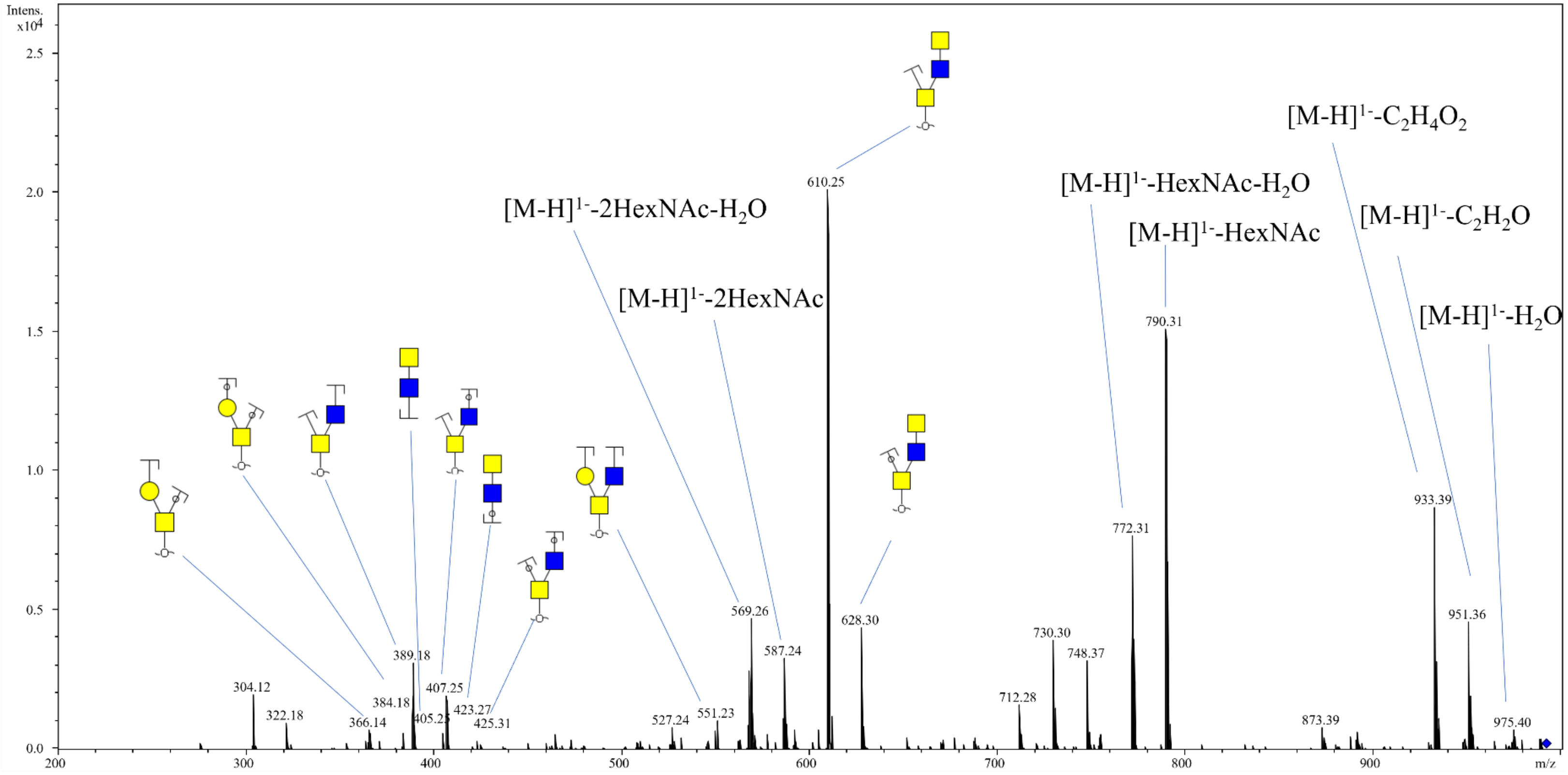

Supporting Figure S1-17: Annotated MS/MS for *O*-glycans released from PSM and PaTu-S cell line (Glycan 17).

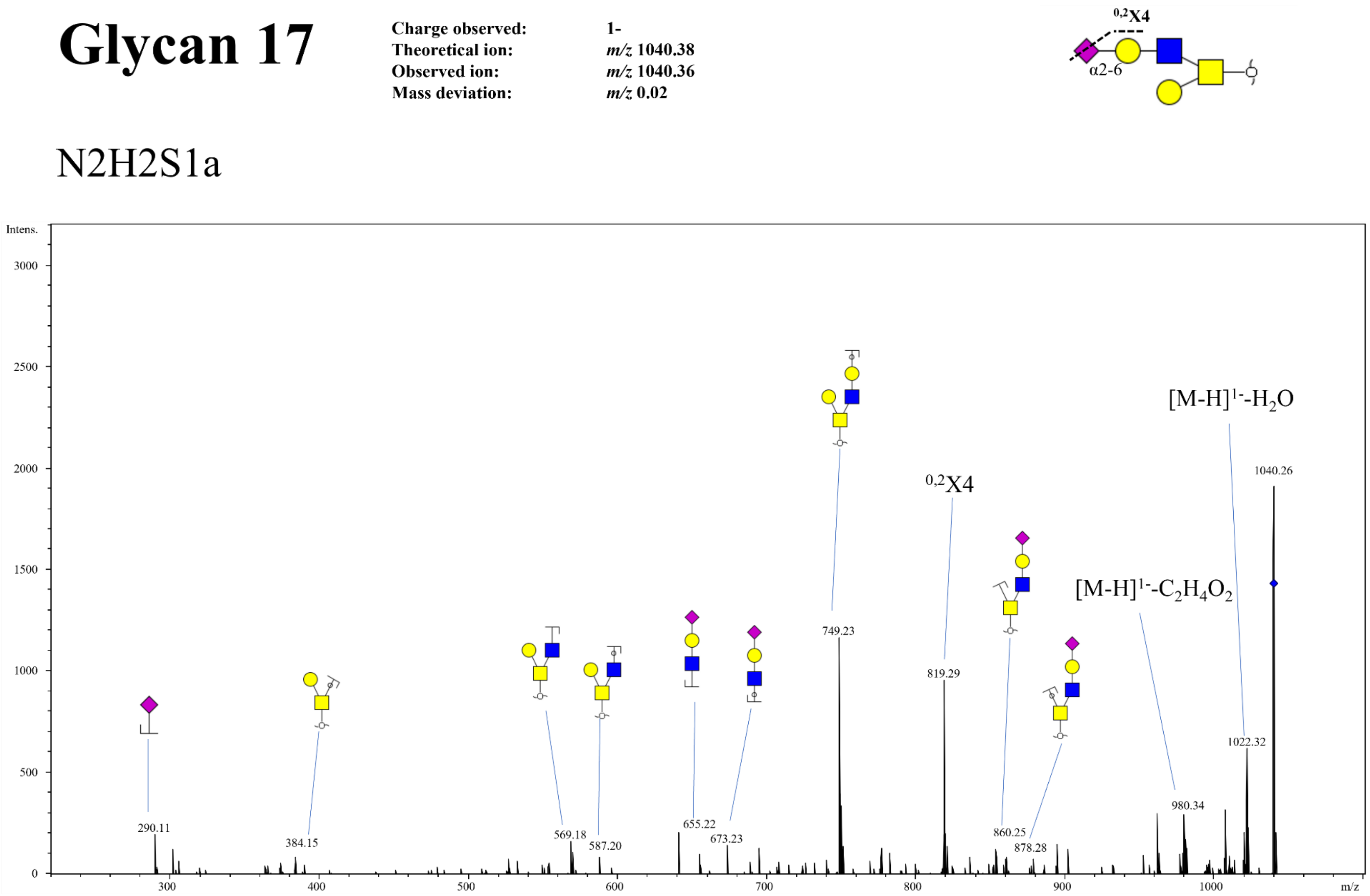

Supporting Figure S1-18: Annotated MS/MS for *O*-glycans released from PSM and PaTu-S cell line (Glycan 18).

Glycan 18

Charge observed: 1-
Theoretical ion: *m/z* 1040.38
Observed ion: *m/z* 1040.36
Mass deviation: *m/z* 0.02

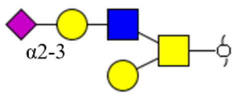

N2H2S1b

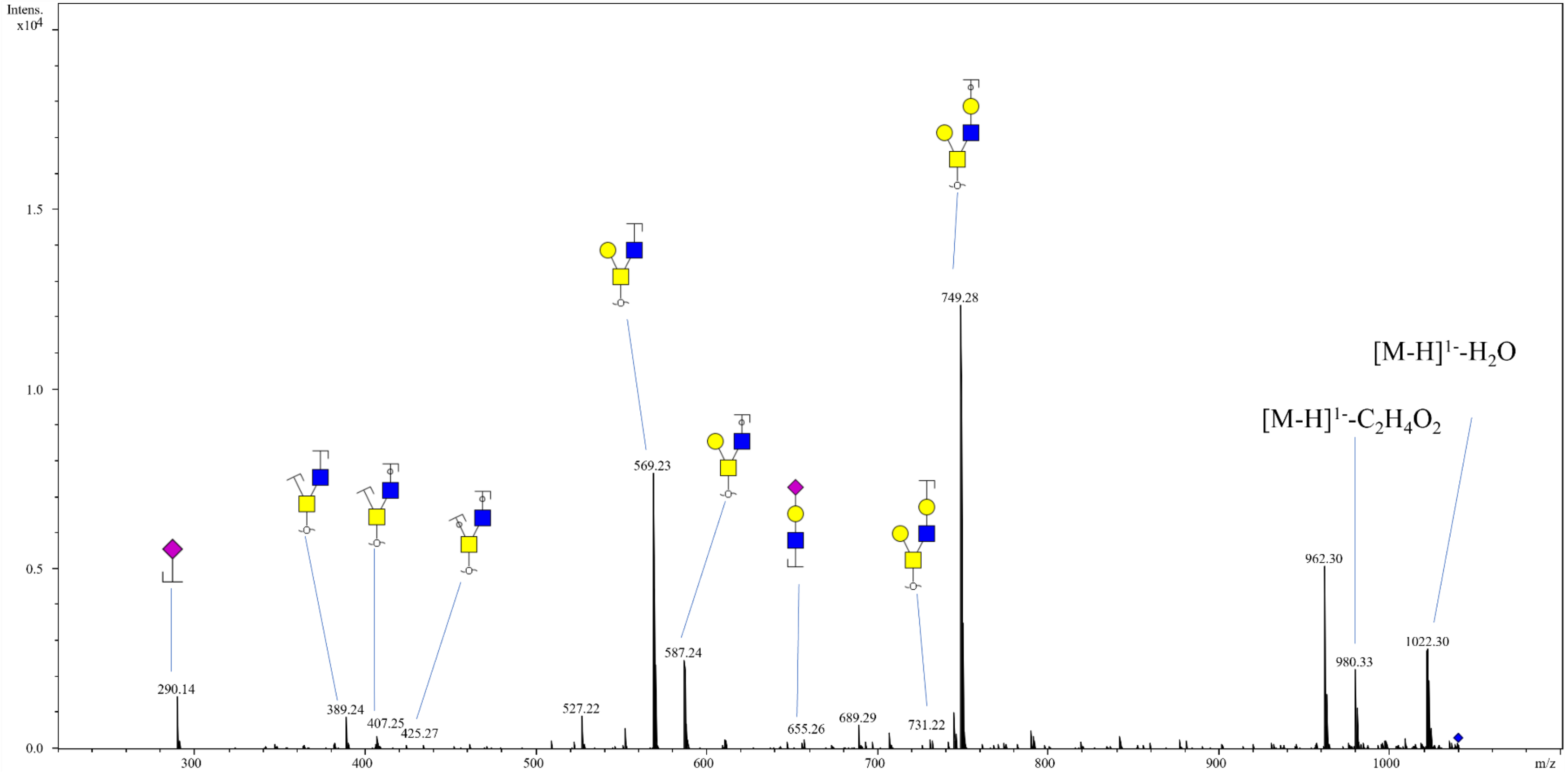

Supporting Figure S1-19: Annotated MS/MS for *O*-glycans released from PSM and PaTu-S cell line (Glycan 19).

Glycan 19

Charge observed: 1-
Theoretical ion: ***m/z*** **1040.38**
Observed ion: ***m/z*** **1040.31**
Mass deviation: ***m/z*** **0.07**

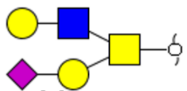

N2H2S1c

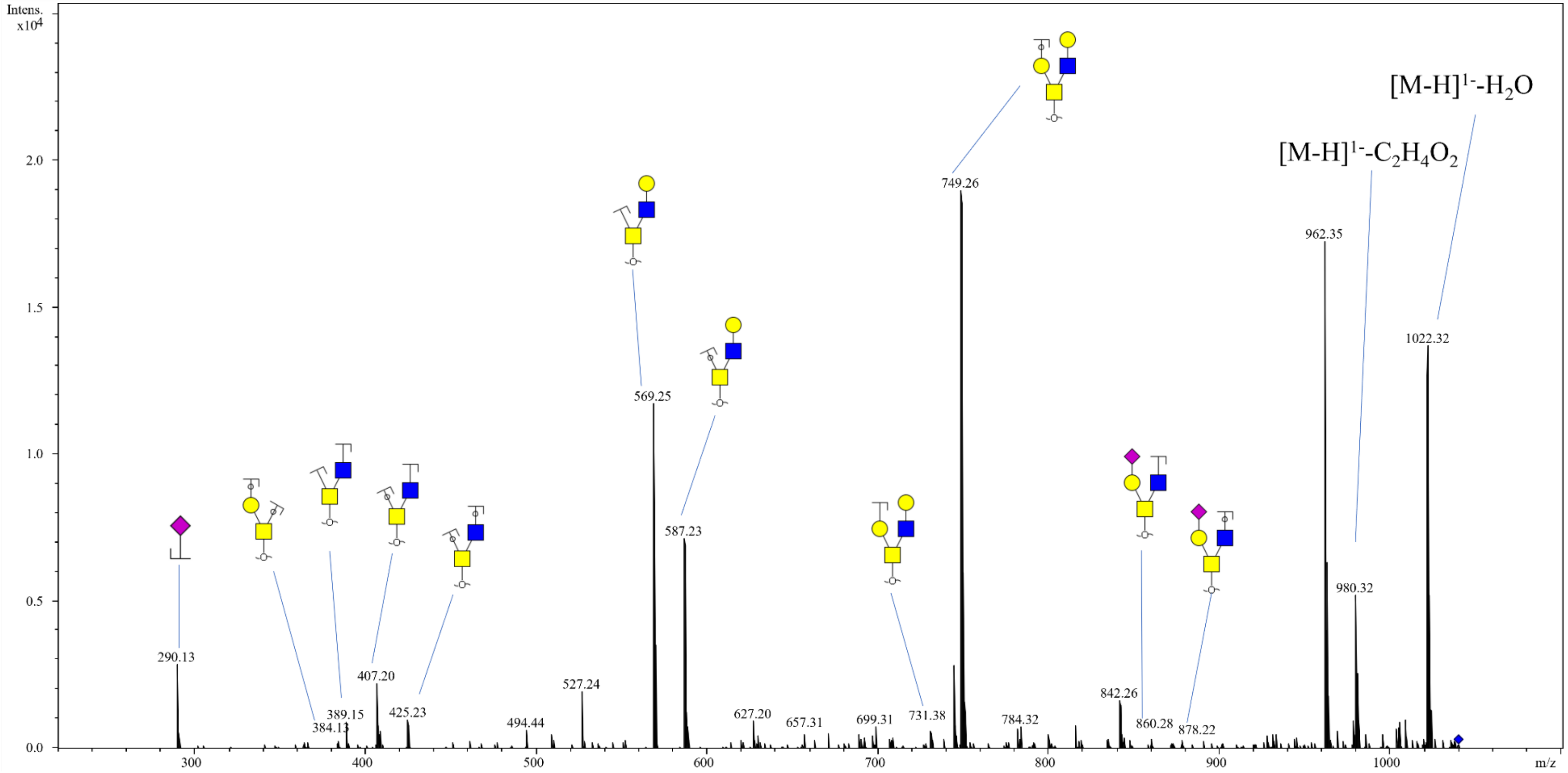

Supporting Figure S1-20: Annotated MS/MS for *O*-glycans released from PSM and PaTu-S cell line (Glycan 20).

Glycan 20

Charge observed: 1-
Theoretical ion: *m/z* 1040.38
Observed ion: *m/z* 1040.31
Mass deviation: *m/z* 0.07

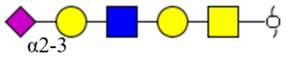

N2H2S1d

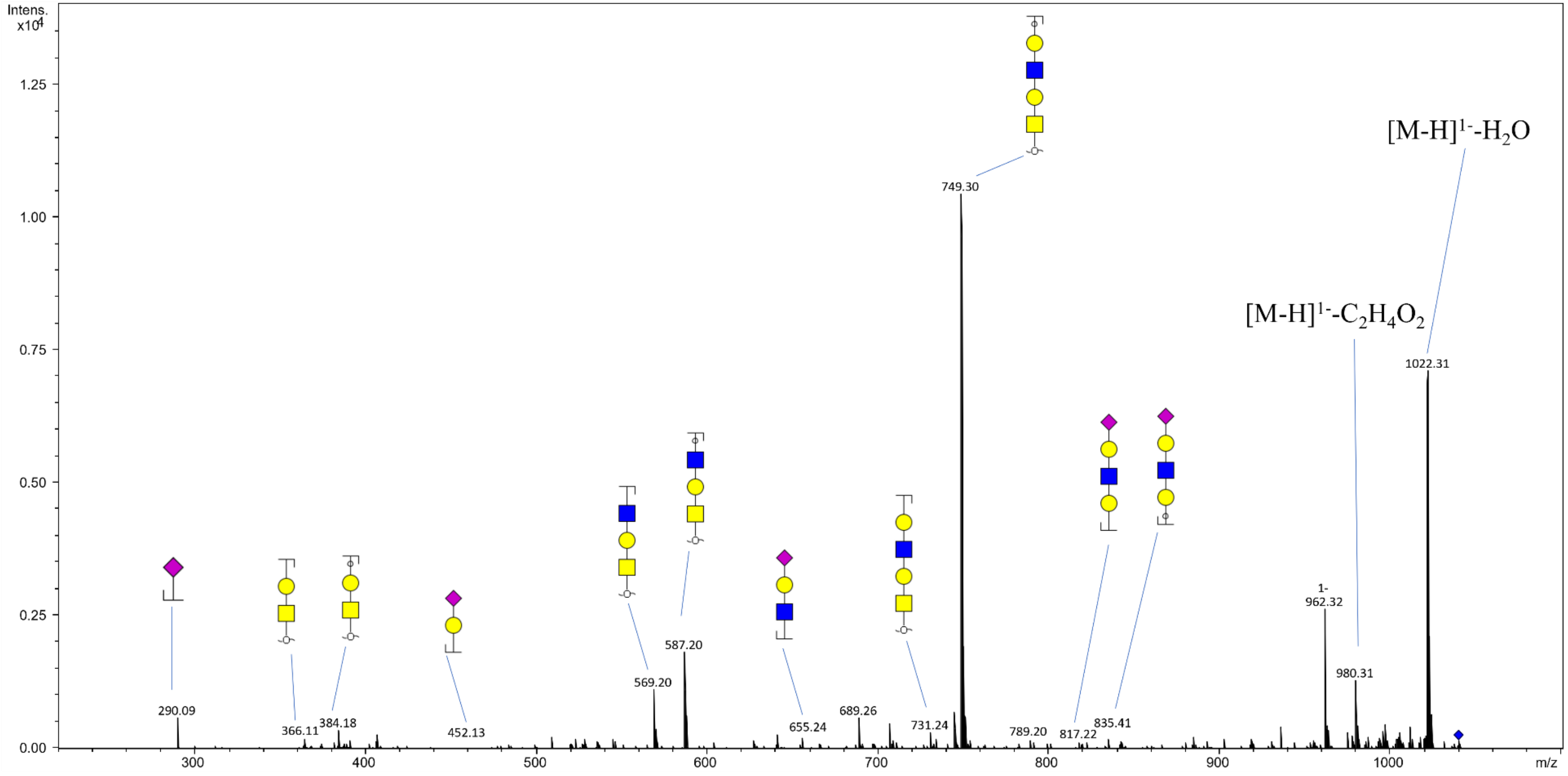

Supporting Figure S1-21: Annotated MS/MS for *O*-glycans released from PSM and PaTu-S cell line (Glycan 21).

Glycan 21

Charge observed: 1-
Theoretical ion: *m/z* 1098.42
Observed ion: *m/z* 1098.41
Mass deviation: *m/z* 0.01

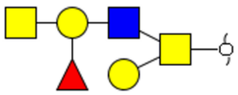

N3H2F1a

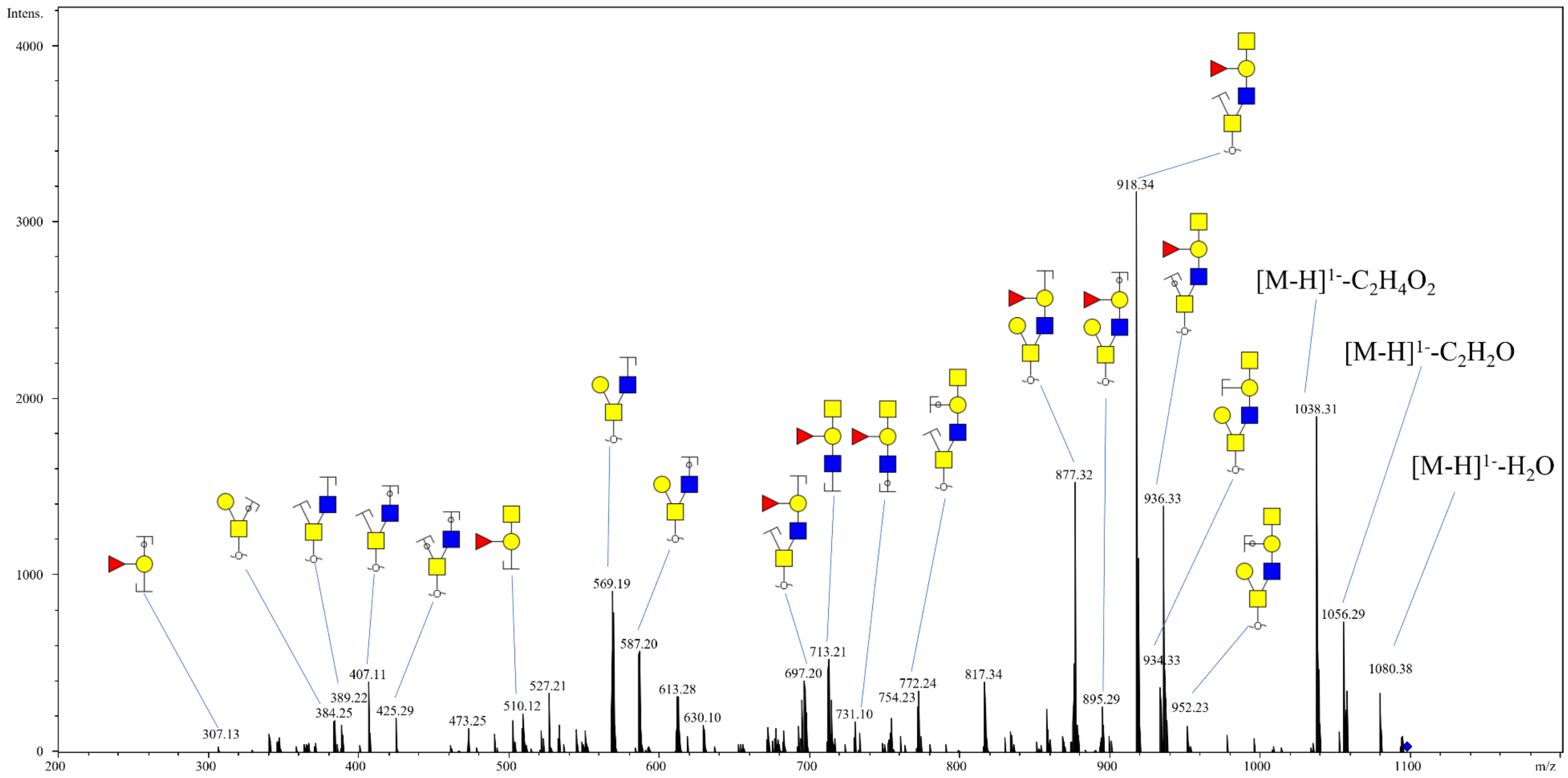

Supporting Figure S1-22: Annotated MS/MS for *O*-glycans released from PSM and PaTu-S cell line (Glycan 22).

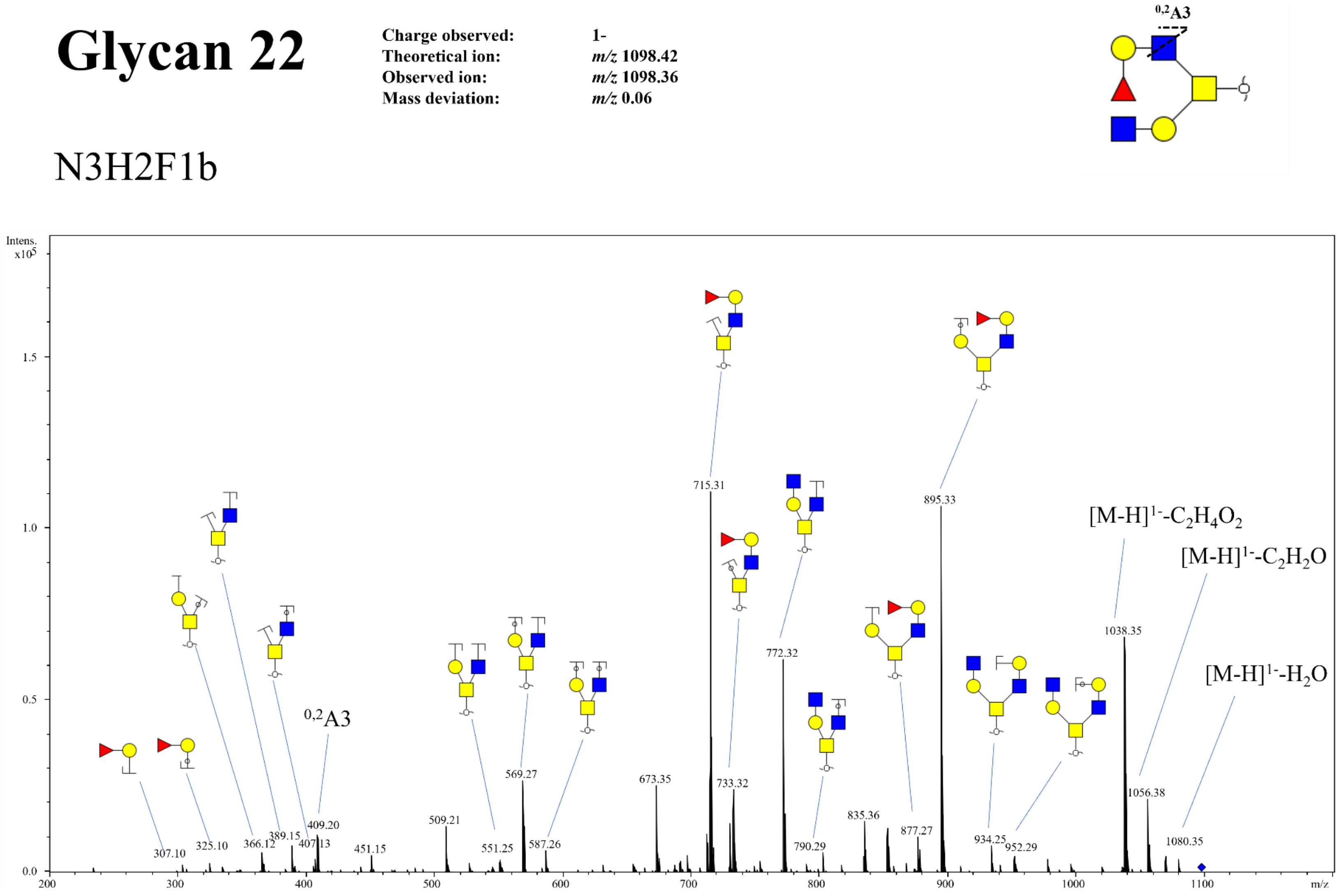

Supporting Figure S1-23: Annotated MS/MS for *O*-glycans released from PSM and PaTu-S cell line (Glycan 23).

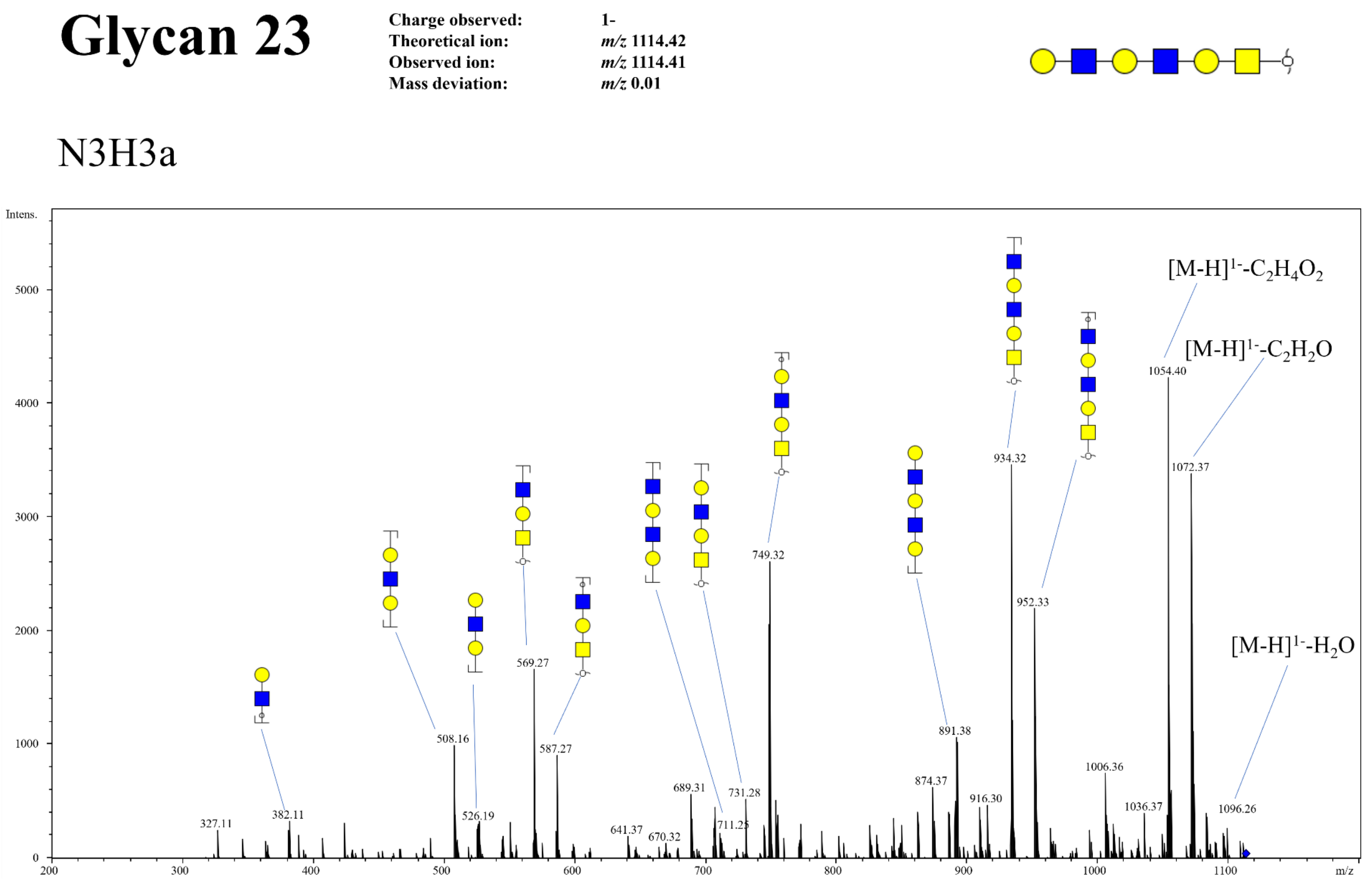

Supporting Figure S1-24: Annotated MS/MS for *O*-glycans released from PSM and PaTu-S cell line (Glycan 24).

Glycan 24

Charge observed: **1-**
Theoretical ion: ***m/z*** **1114.42**
Observed ion: ***m/z*** **1114.43**
Mass deviation: ***m/z*** **0.01**

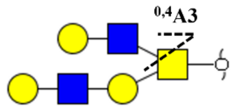

N3H3b

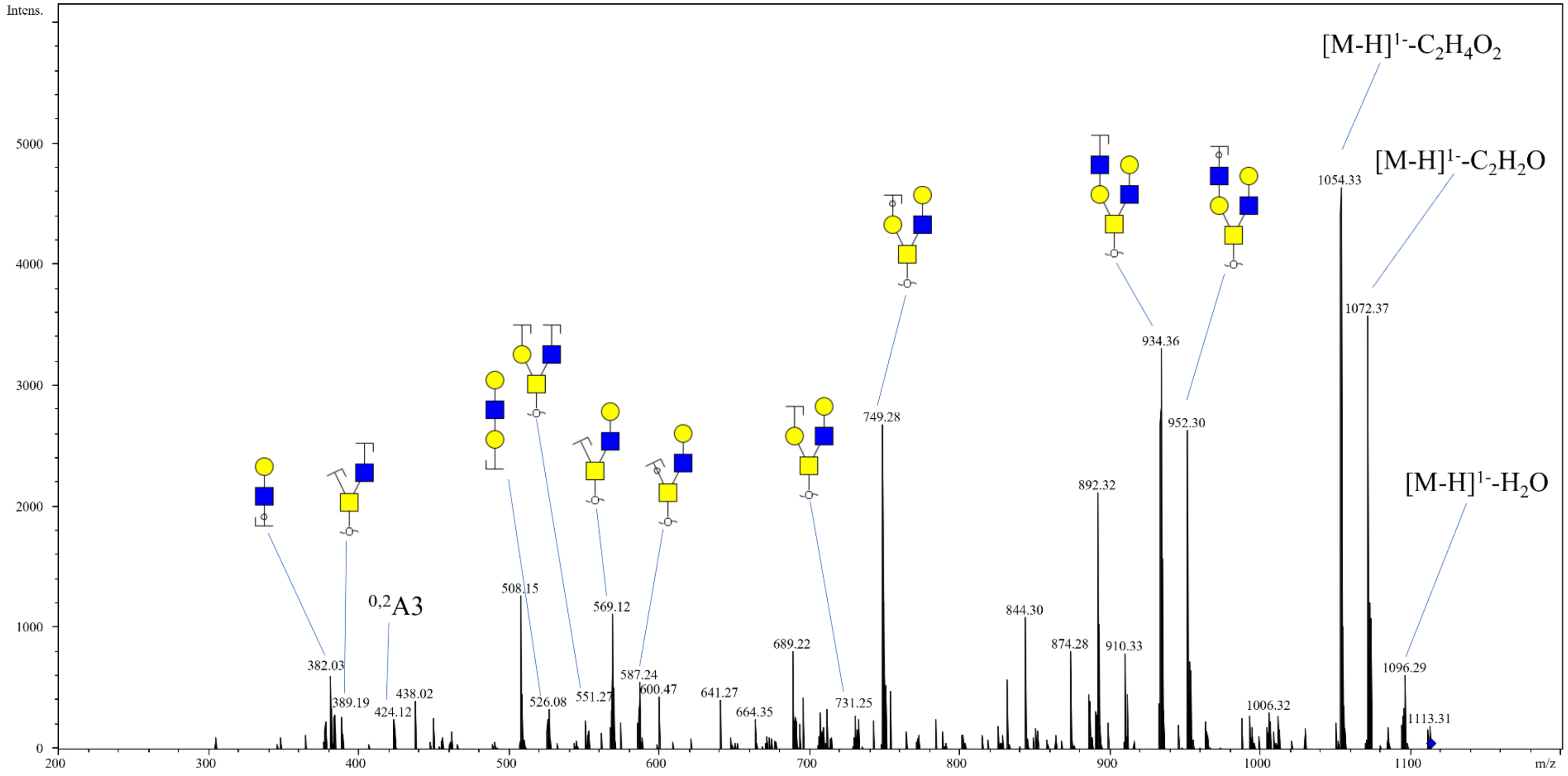

Supporting Figure S1-25: Annotated MS/MS for *O*-glycans released from PSM and PaTu-S cell line (Glycan 25).

Glycan 25

Charge observed: 1-
Theoretical ion: *m/z* 1155.44
Observed ion: *m/z* 1155.42
Mass deviation: *m/z* 0.02

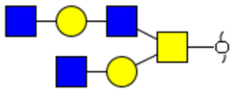

N4H2a

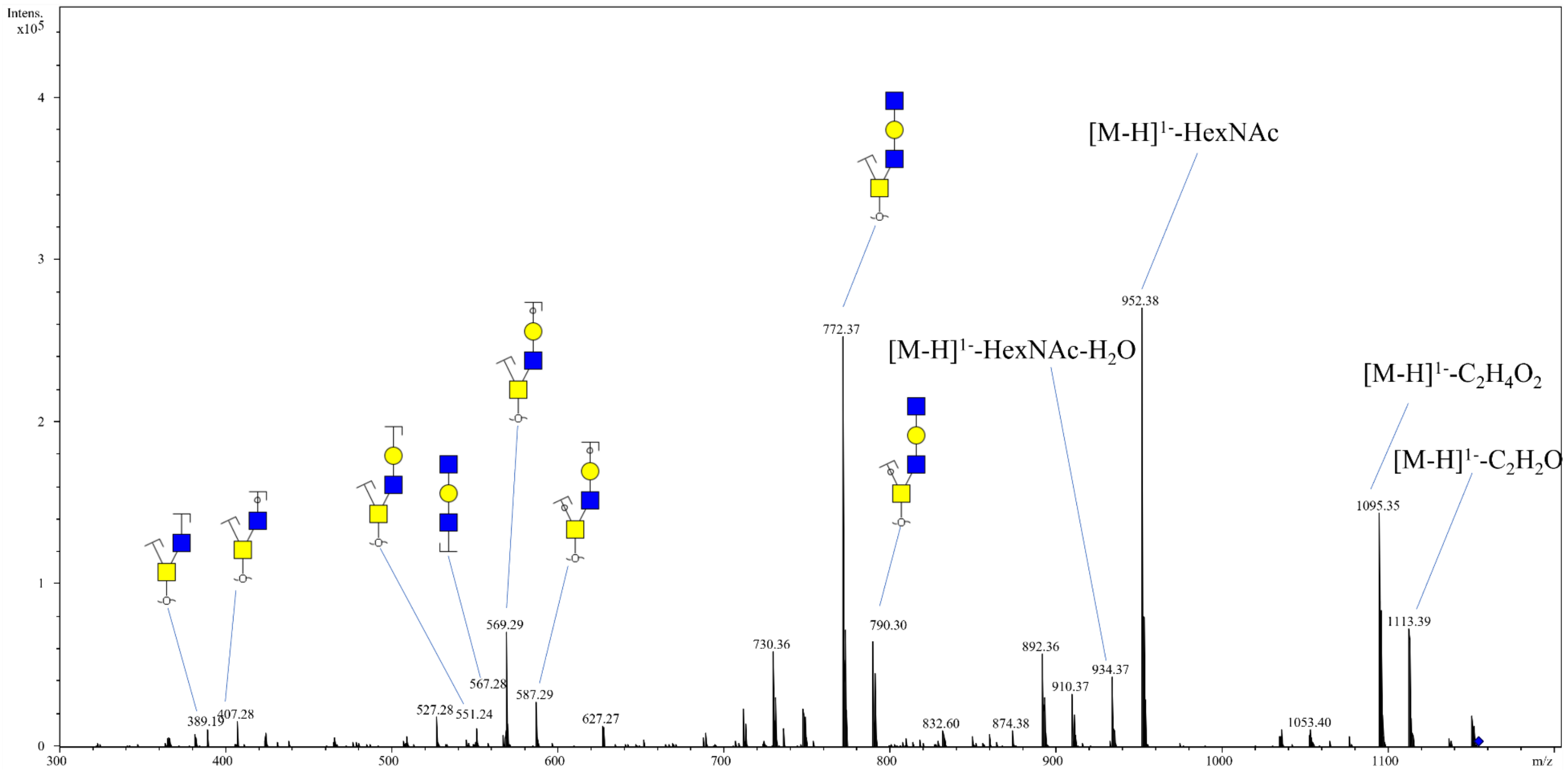

Supporting Figure S1-26: Annotated MS/MS for *O*-glycans released from PSM and PaTu-S cell line (Glycan 26).

Glycan 26

Charge observed: 1-
Theoretical ion: *m/z* 1155.44
Observed ion: *m/z* 1155.39
Mass deviation: *m/z* 0.05

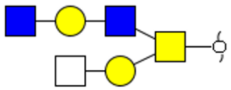

N4H2b

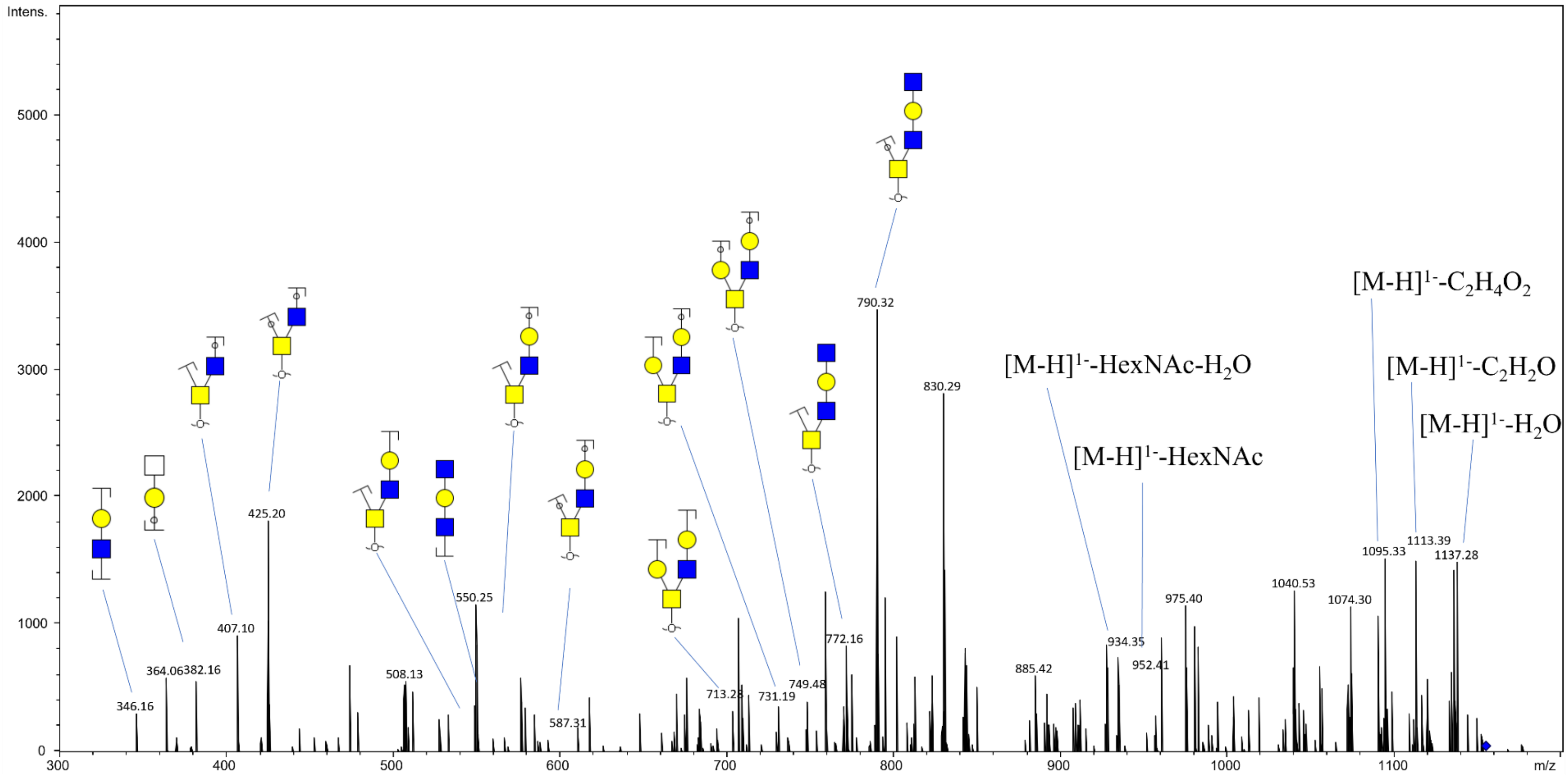

Supporting Figure S1-27: Annotated MS/MS for *O*-glycans released from PSM and PaTu-S cell line (Glycan 27).

Glycan 27

**Charge observed:**	**1-**
**Theoretical ion:**	***m/z* 1186.44**
**Observed ion:**	***m/z* 1186.48**
**Mass deviation:**	***m/z* -0.04**

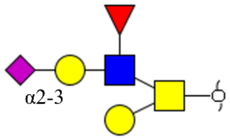

N2H2F1S1a

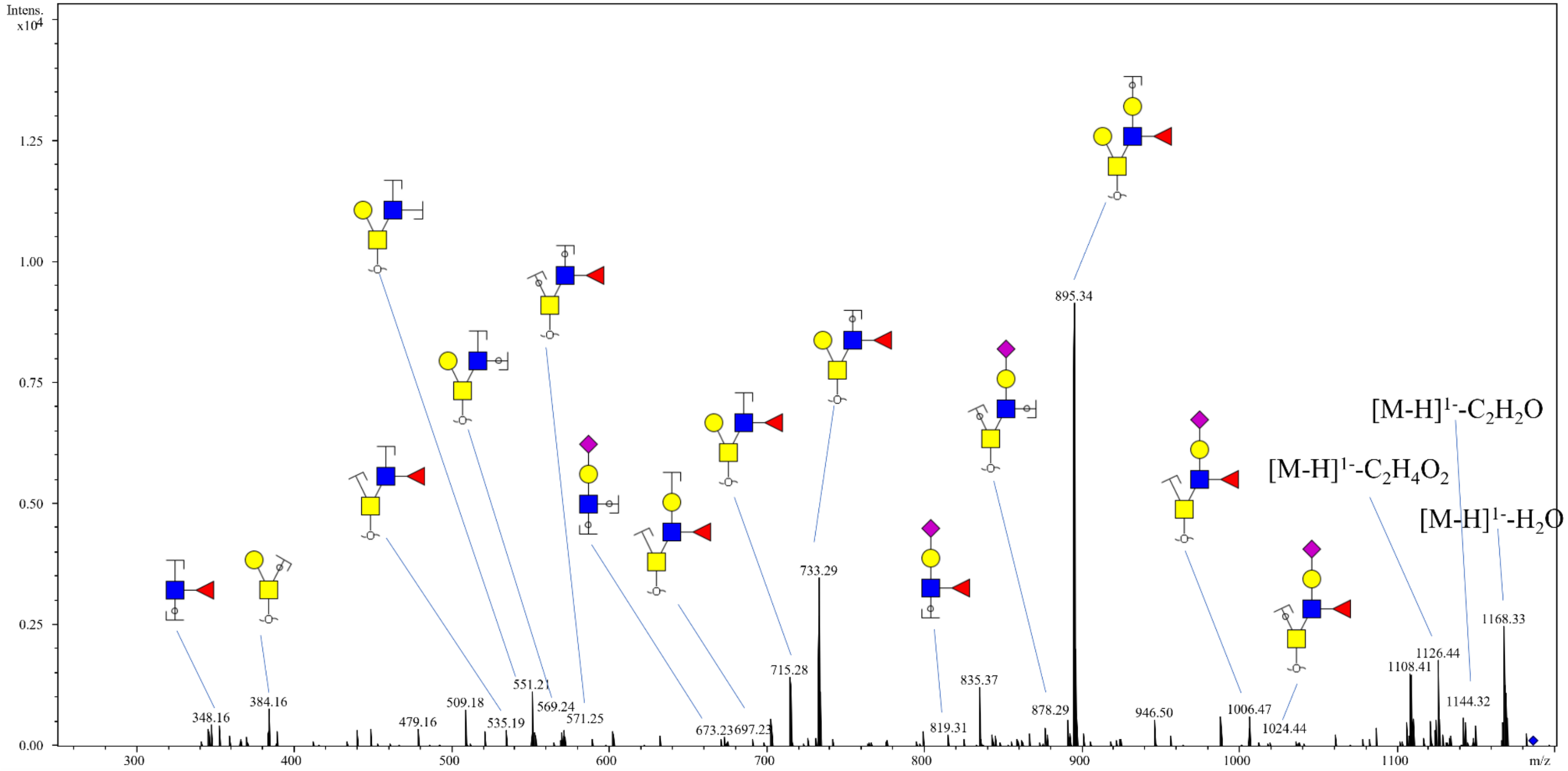

Supporting Figure S1-28: Annotated MS/MS for *O*-glycans released from PSM and PaTu-S cell line (Glycan 28).

Glycan 28

Charge observed: 1-
Theoretical ion: *m/z* 1186.44
Observed ion: *m/z* 1186.45
Mass deviation: *m/z* -0.01

N2H2F1S1b

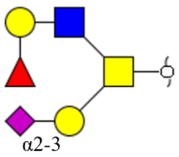

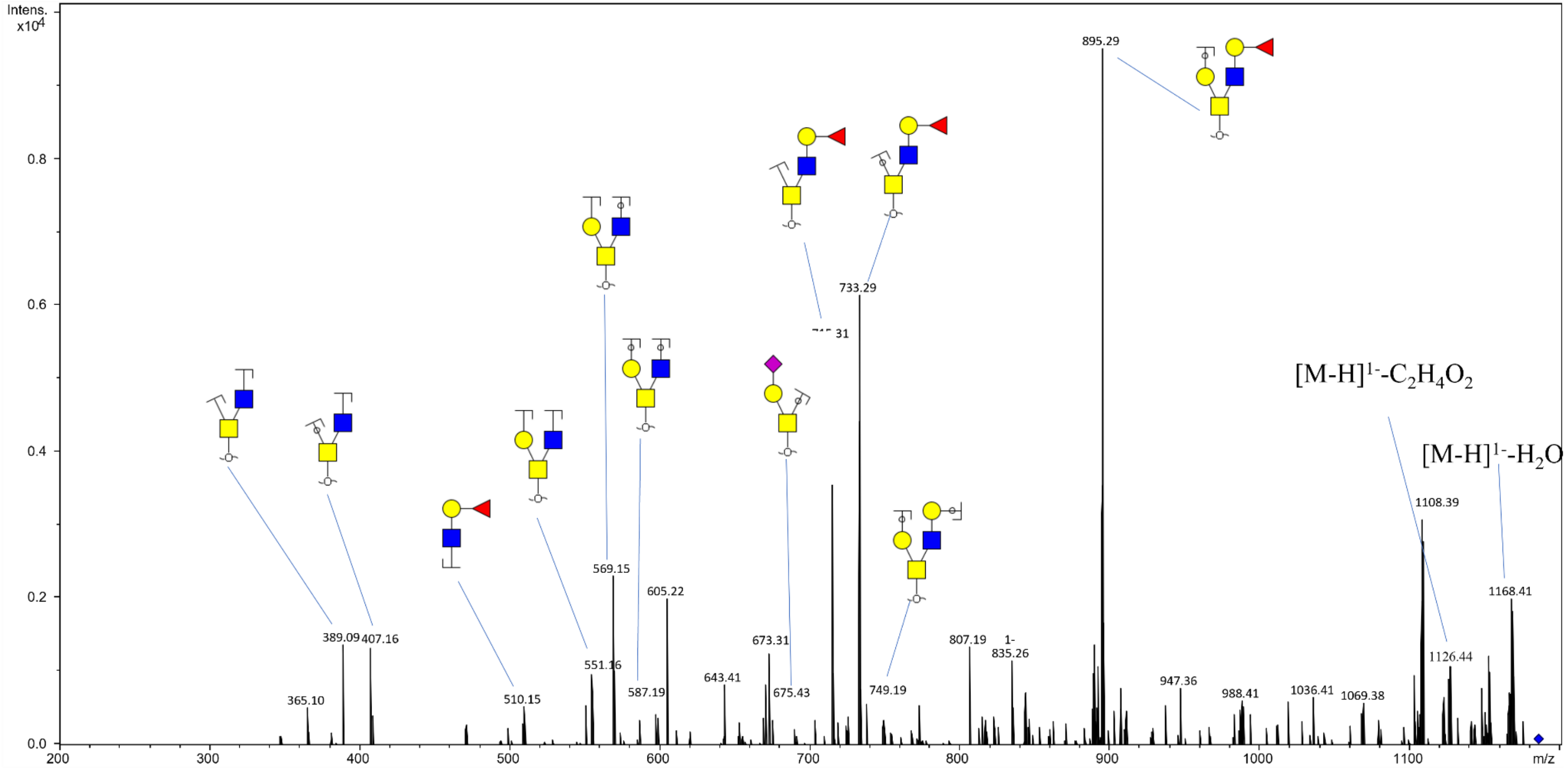

Supporting Figure S1-29: Annotated MS/MS for *O*-glycans released from PSM and PaTu-S cell line (Glycan 29).

Glycan 29

Charge observed: 1-
Theoretical ion: *m/z* 1227.46
Observed ion: *m/z* 1227.38
Mass deviation: *m/z* 0.08

N3H1F1S1

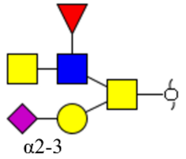

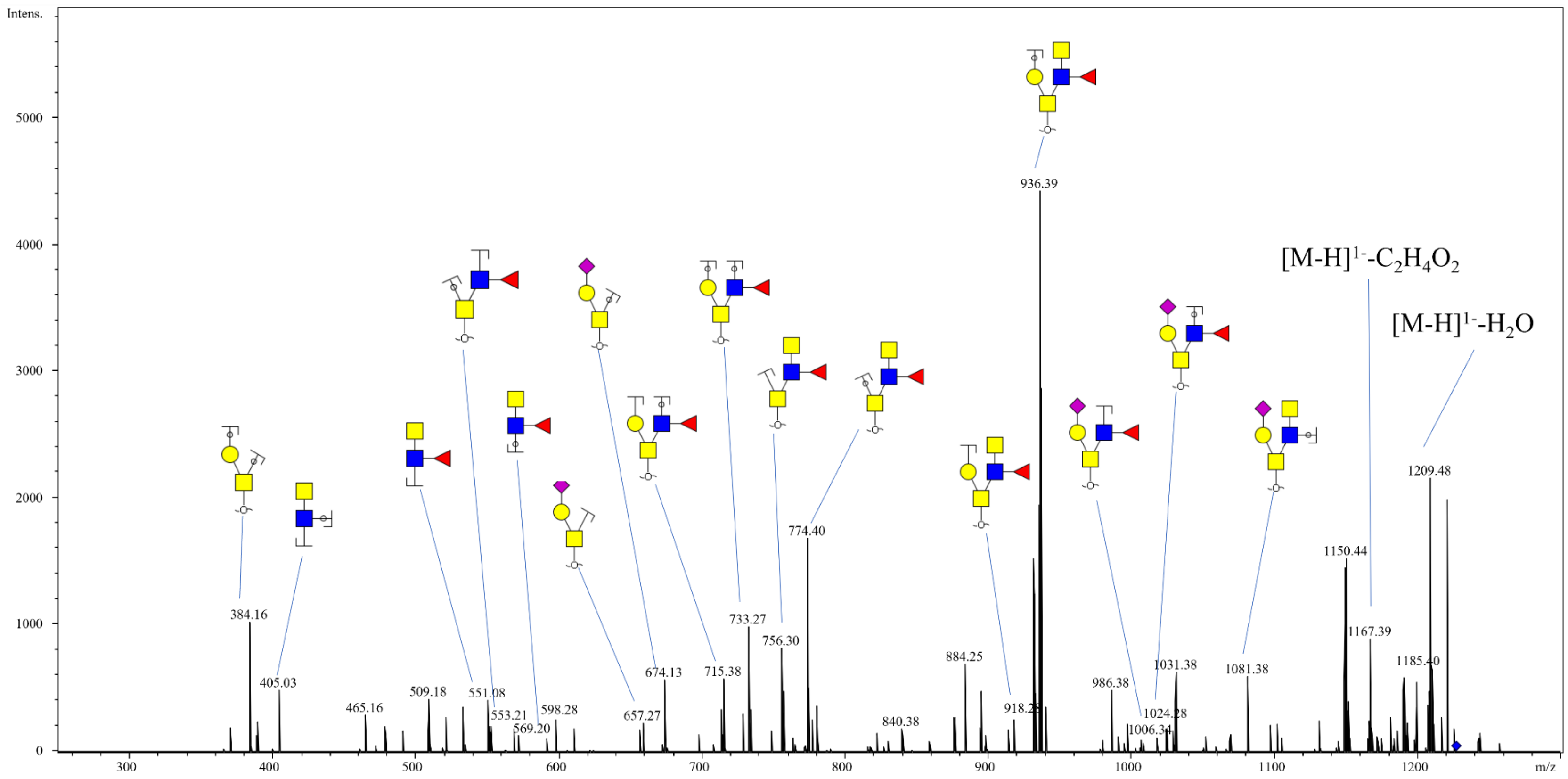

Supporting Figure S1-30: Annotated MS/MS for *O*-glycans released from PSM and PaTu-S cell line (Glycan 30).

Glycan 30

Charge observed: 1-
Theoretical ion: ***m/z*** **1331.47**
Observed ion: ***m/z*** **1331.41**
Mass deviation: ***m/z*** **0.06**

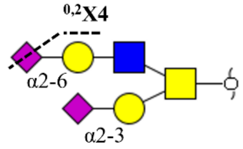

N3H2S2a

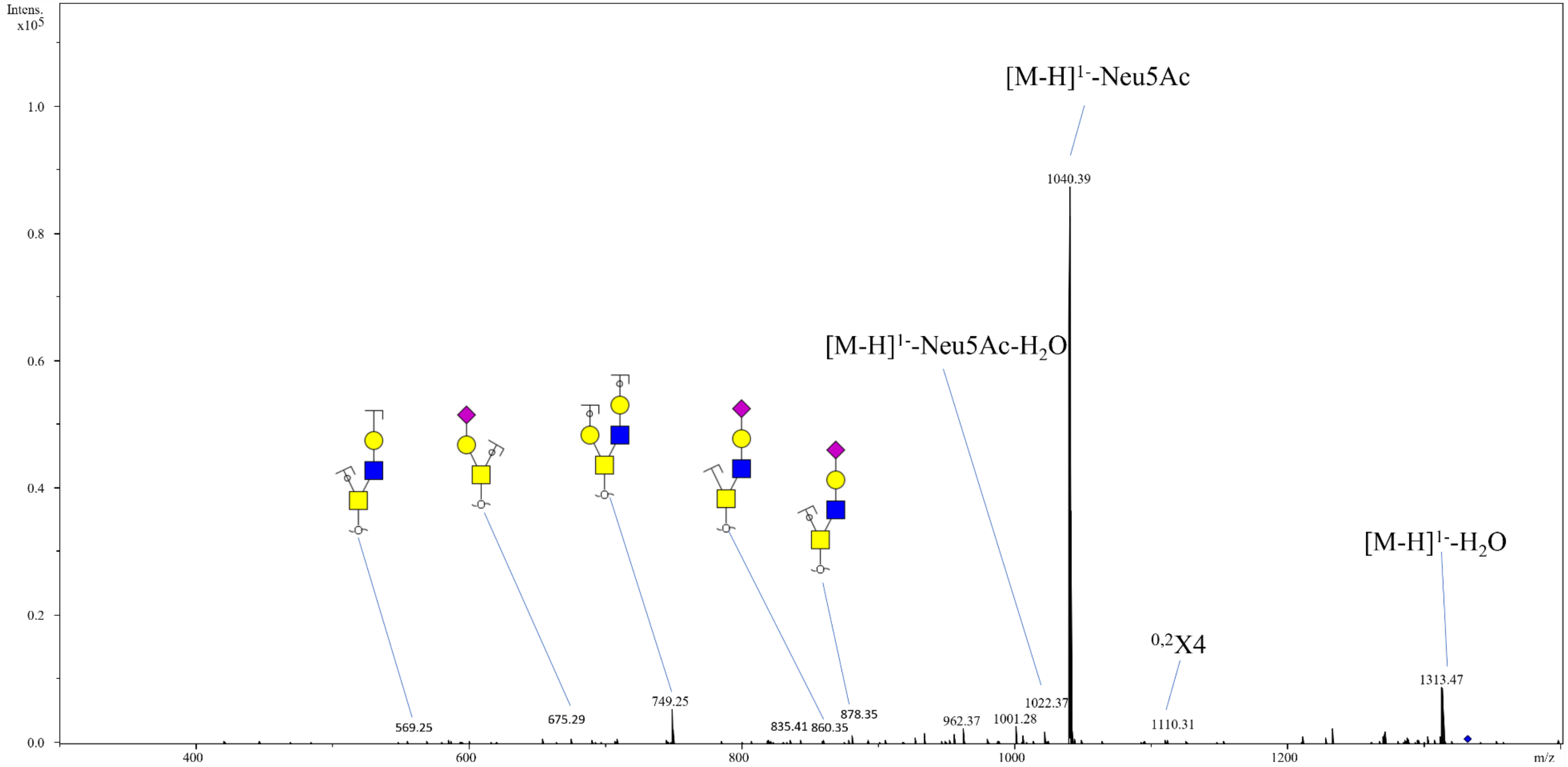

Supporting Figure S1-31: Annotated MS/MS for *O*-glycans released from PSM and PaTu-S cell line (Glycan 31).

Glycan 31

Charge observed: 1-
Theoretical ion: ***m/z*** **1331.47**
Observed ion: ***m/z*** **1331.48**
Mass deviation: ***m/z*** **-0.01**

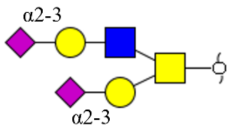

N3H2S2b

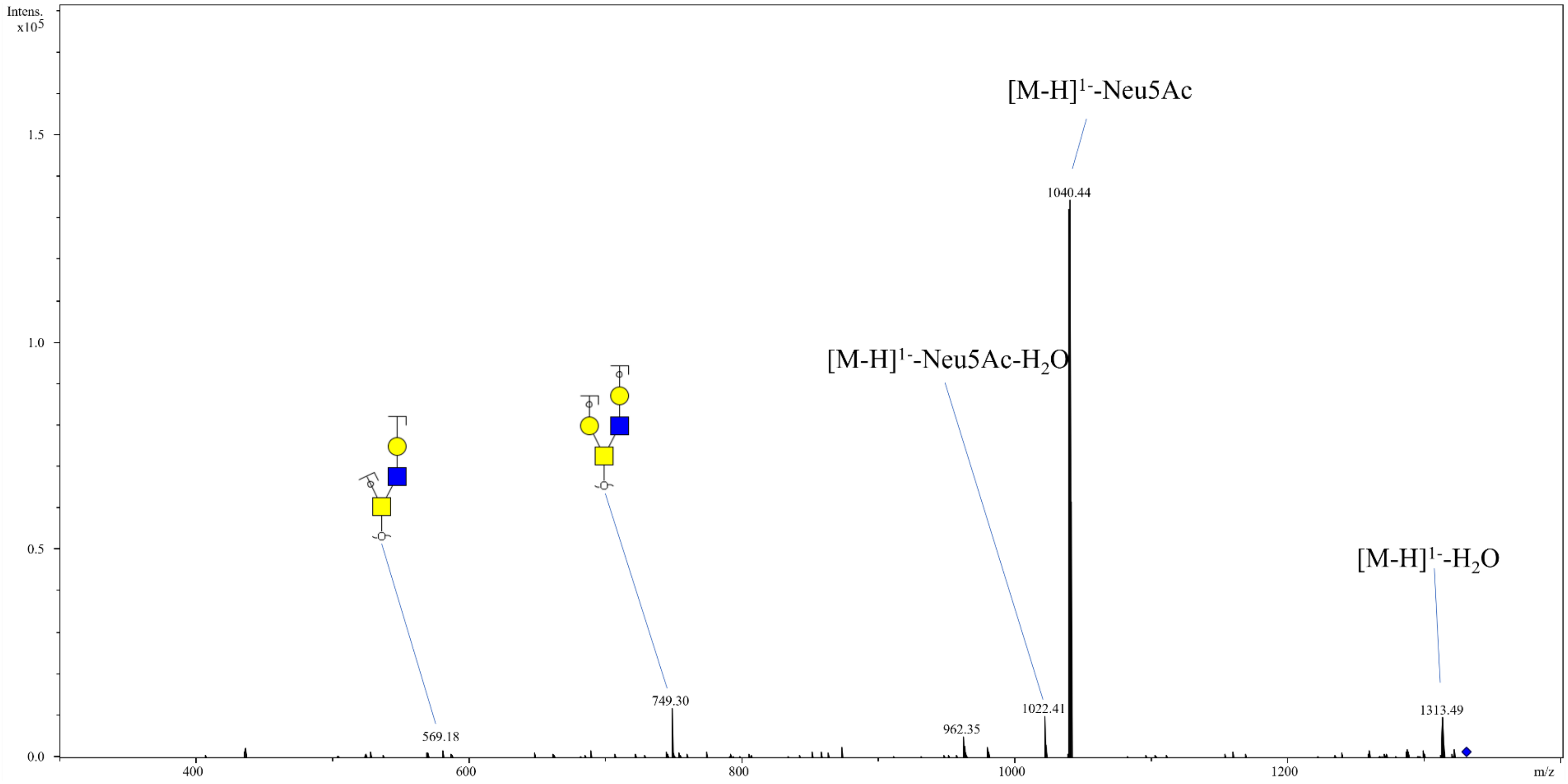

Supporting Figure S1-32: Annotated MS/MS for *O*-glycans released from PSM and PaTu-S cell line (Glycan 32).

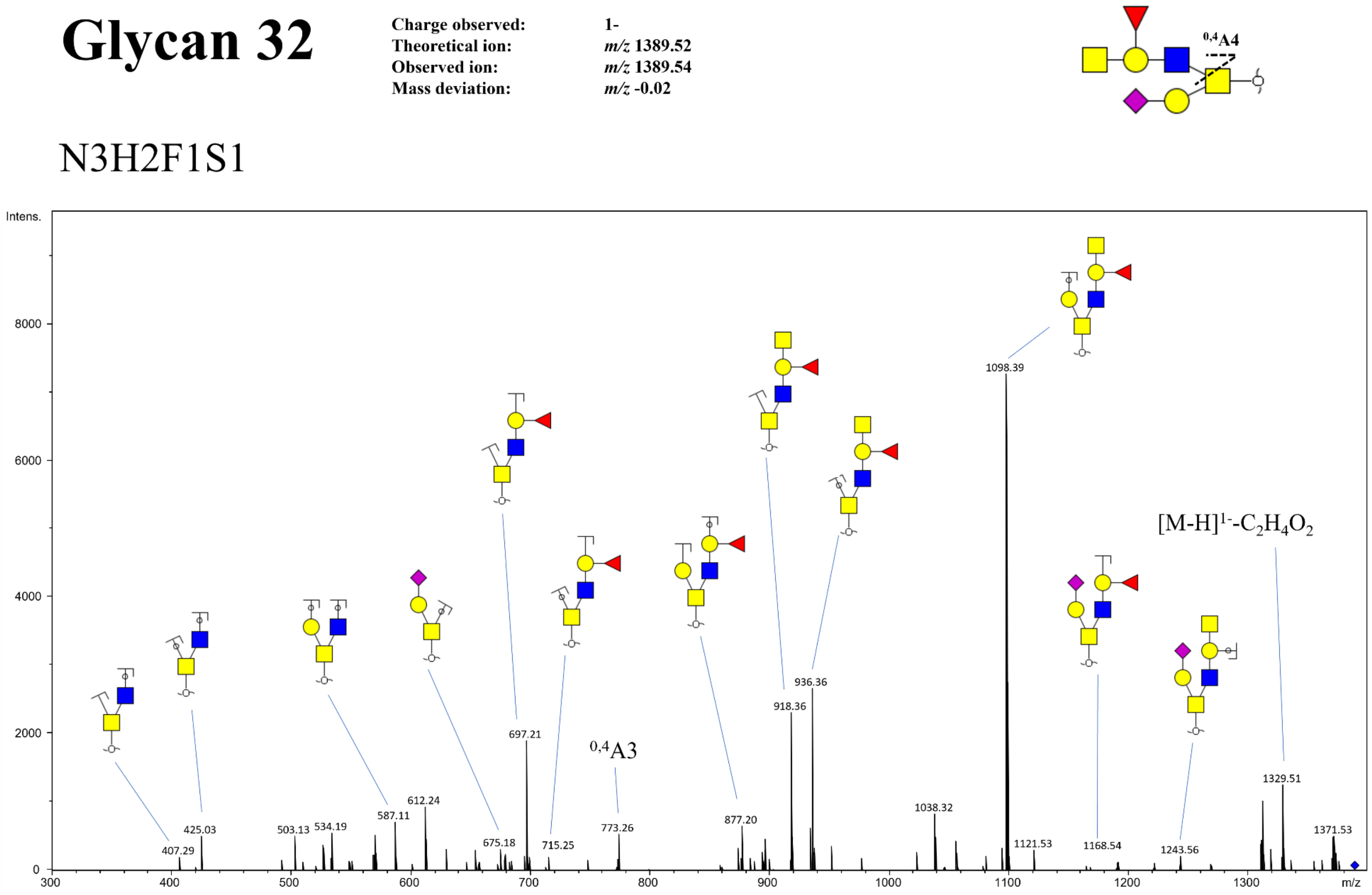

Supporting Figure S1-33: Annotated MS/MS for *O*-glycans released from PSM and PaTu-S cell line (Glycan 33).

Glycan 33

**Charge observed:**	**2-**
**Theoretical ion:**	***m/z* 739.27**
**Observed ion:**	***m/z* 739.23**
**Mass deviation:**	***m/z* 0.04**

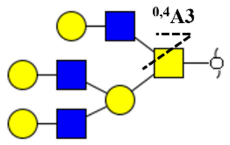

N4H4

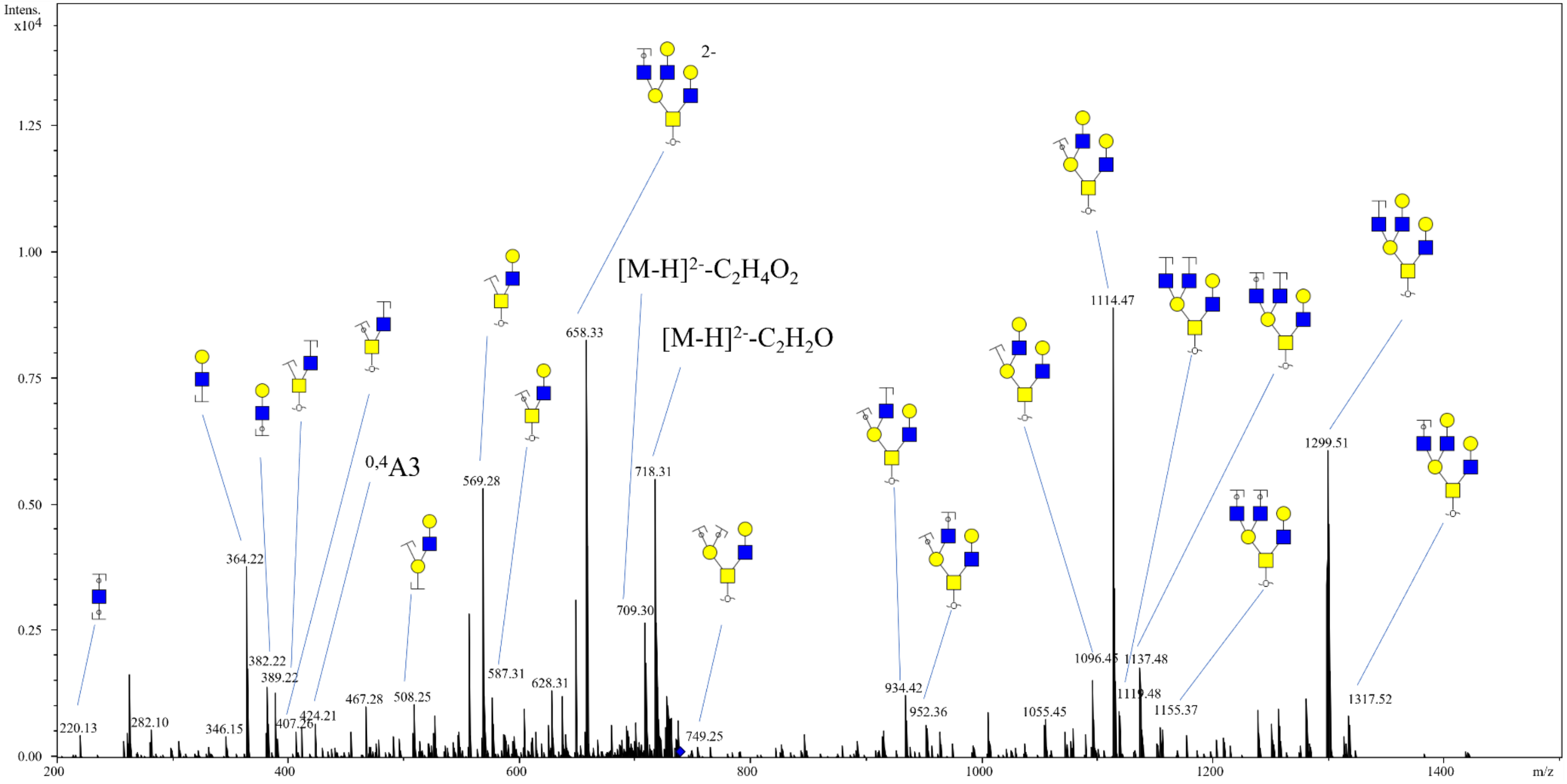

Supporting Figure S1-34: Annotated MS/MS for *O*-glycans released from PSM and PaTu-S cell line (Glycan 34).

Glycan 34

N3H2F2S1

**Charge observed:**	**2-**
**Theoretical ion:**	***m/z* 767.29**
**Observed ion:**	***m/z* 767.28**
**Mass deviation:**	***m/z* 0.01**

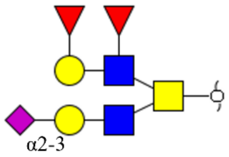

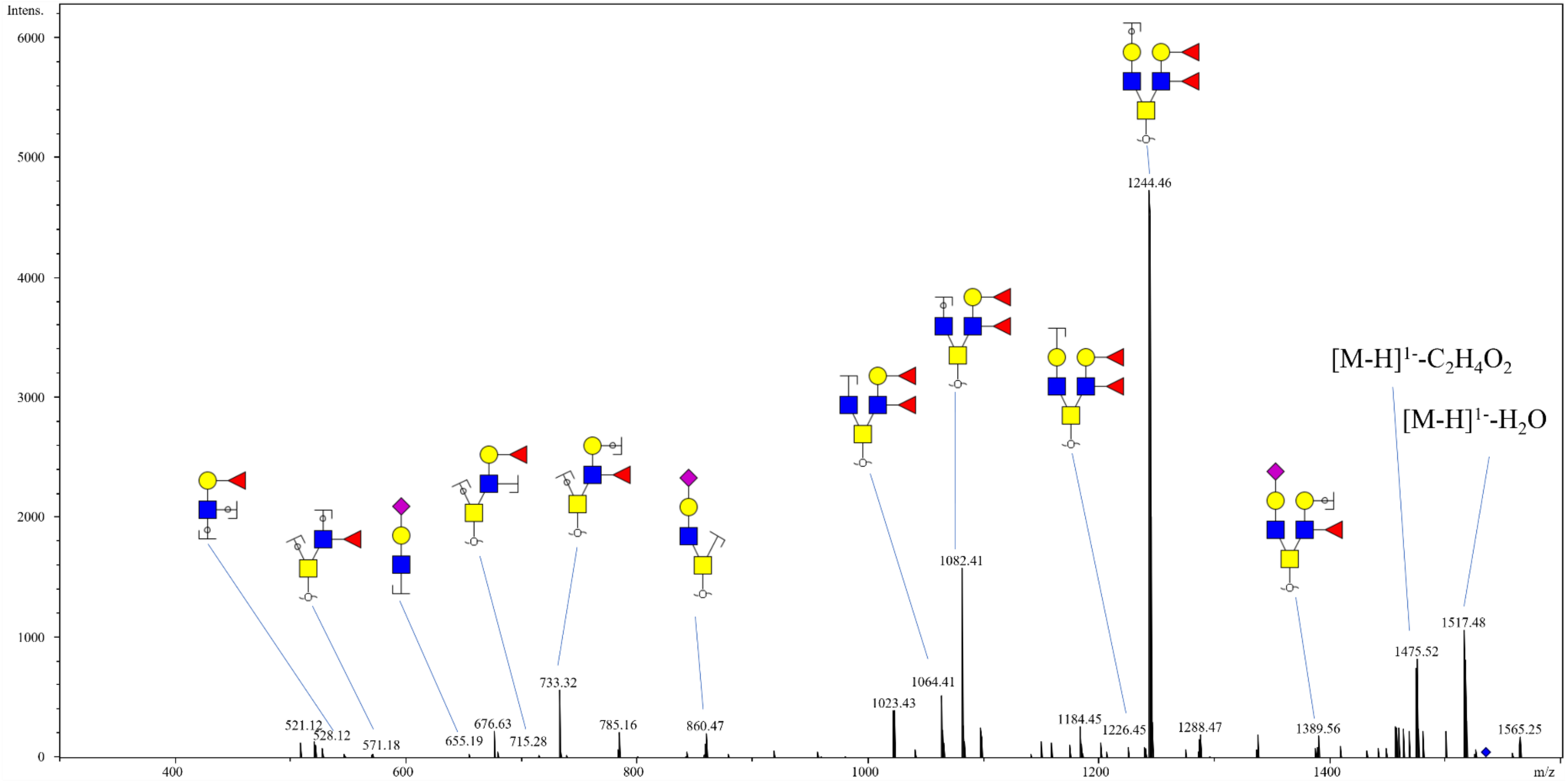

Supporting Figure S1-35: Annotated MS/MS for *O*-glycans released from PSM and PaTu-S cell line (Glycan 35).

Glycan 35

Charge observed: 1-
Theoretical ion: *m/z* 530.21
Observed ion: *m/z* 530.19
Mass deviation: *m/z* 0.02

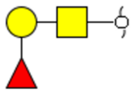

N1H1F1

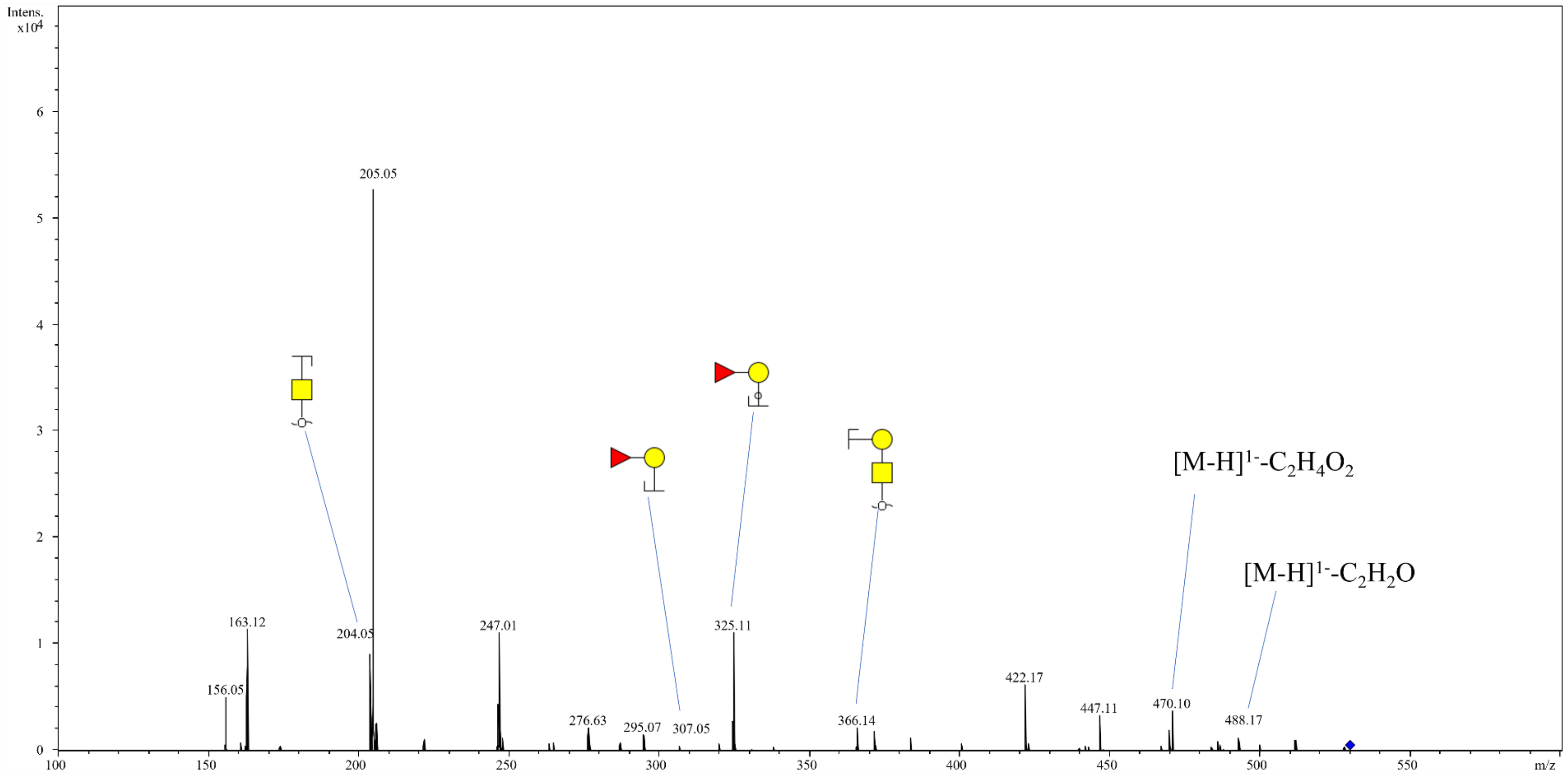

Supporting Figure S1-36: Annotated MS/MS for *O*-glycans released from PSM and PaTu-S cell line (Glycan 36).

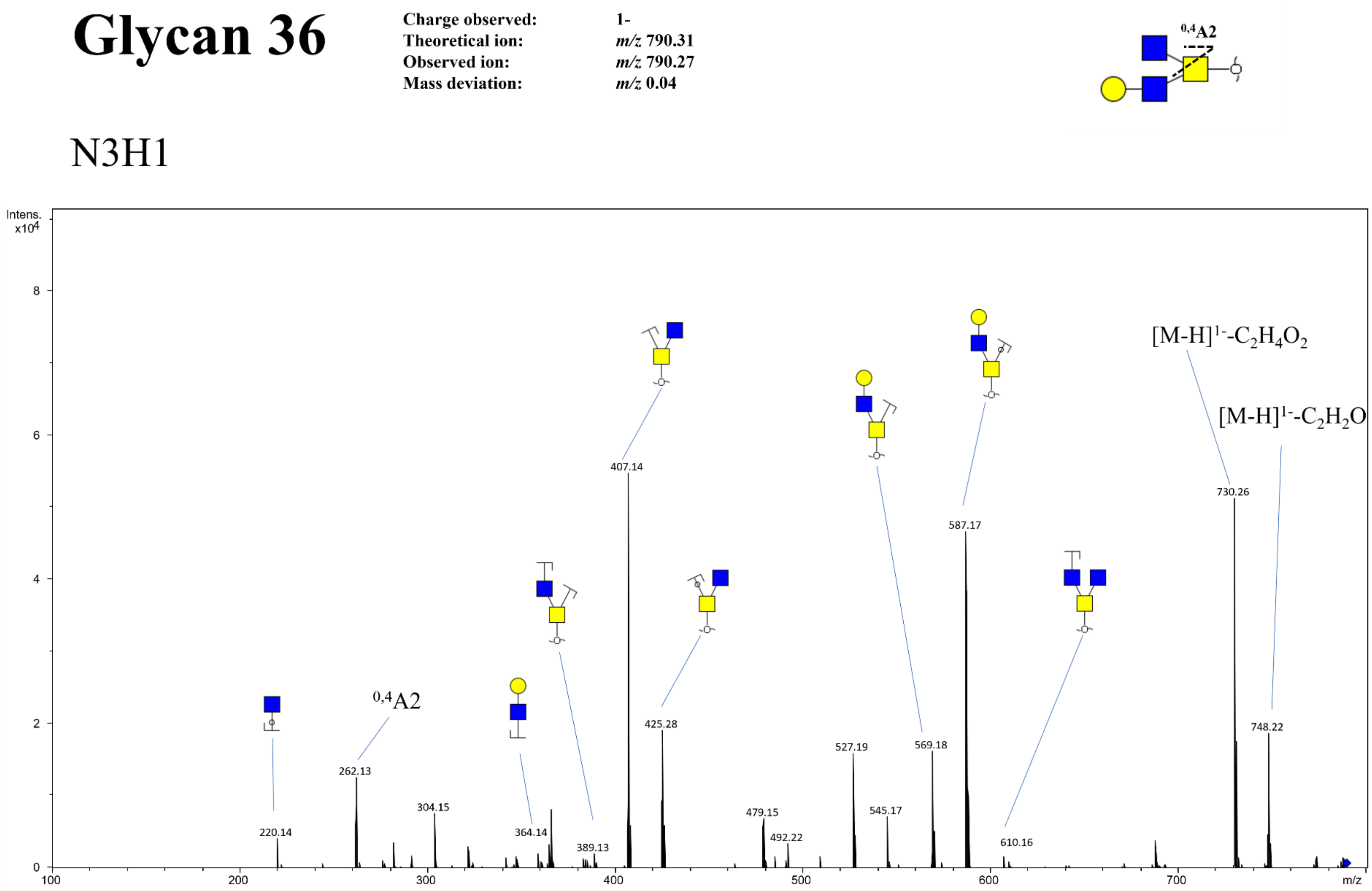

Supporting Figure S1-37: Annotated MS/MS for *O*-glycans released from PSM and PaTu-S cell line (Glycan 37).

Glycan 37

Charge observed: 1-
Theoretical ion: *m/z* 952.36
Observed ion: *m/z* 952.37
Mass deviation: *m/z* -0.01

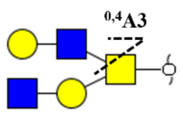

N3H2a

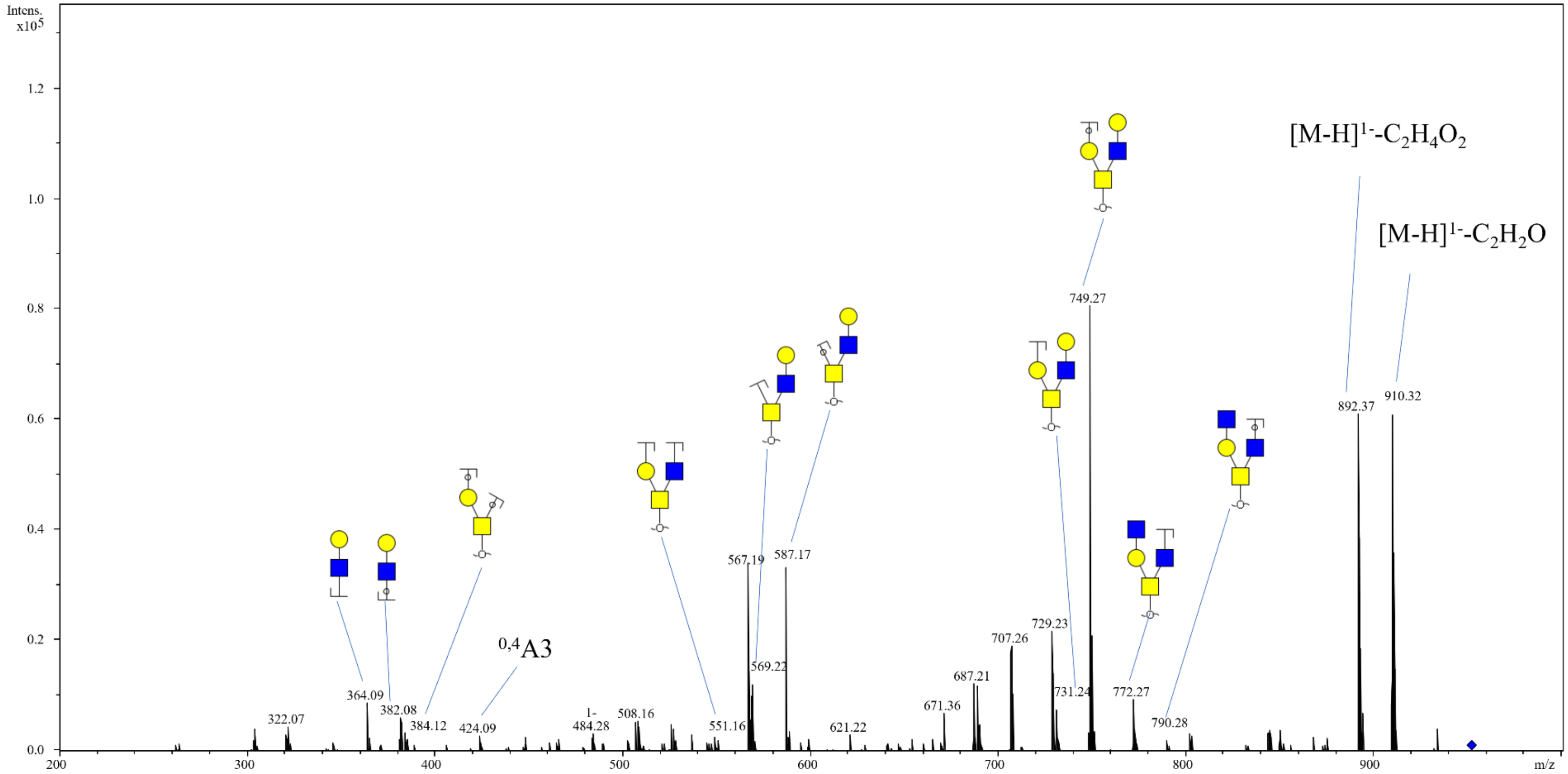

Supporting Figure S1-38: Annotated MS/MS for *O*-glycans released from PSM and PaTu-S cell line (Glycan 38).

Glycan 38

Charge observed: 1-
Theoretical ion: *m/z* 952.36
Observed ion: *m/z* 952.36
Mass deviation: *m/z* 0.00

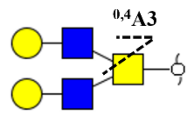

N3H2b

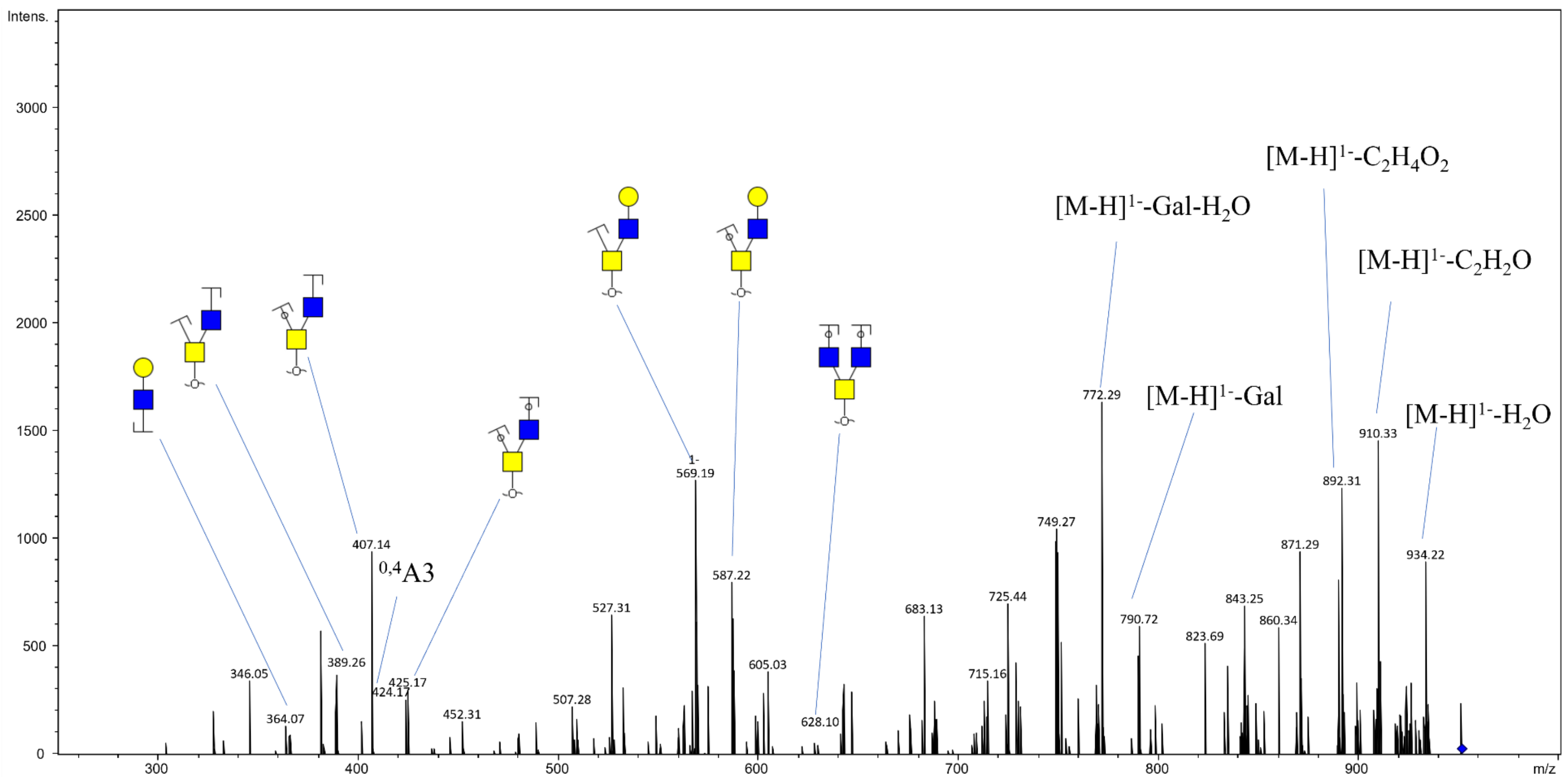

Supporting Figure S1-39: Annotated MS/MS for *O*-glycans released from PSM and PaTu-S cell line (Glycan 39).

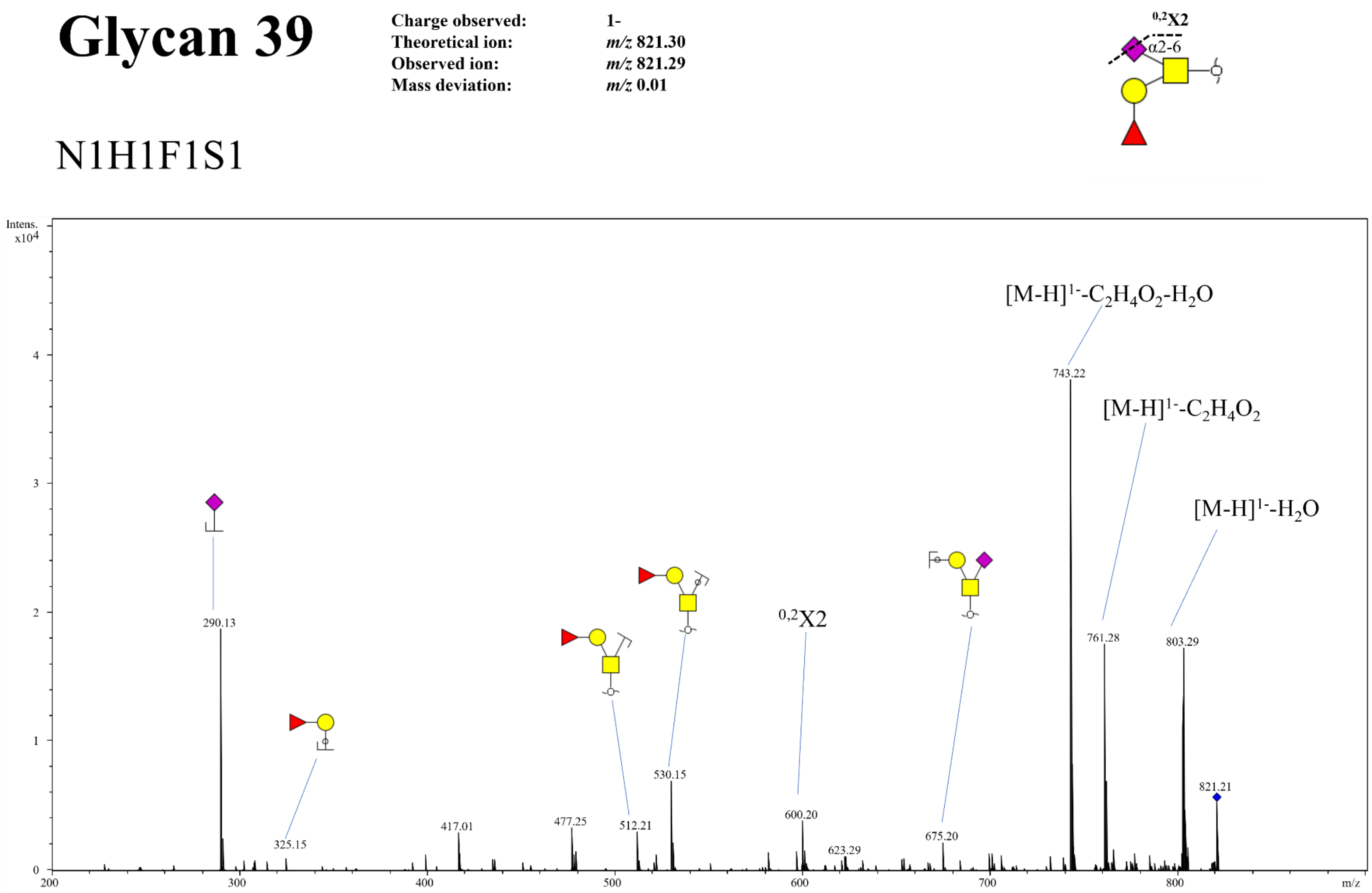

Supporting Figure S1-40: Annotated MS/MS for *O*-glycans released from PSM and PaTu-S cell line (Glycan 40).

Glycan 40

Charge observed: 1-
Theoretical ion: *m/z* 733.29
Observed ion: *m/z* 733.27
Mass deviation: *m/z* 0.02

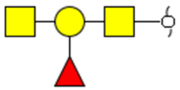

N2H1F1a

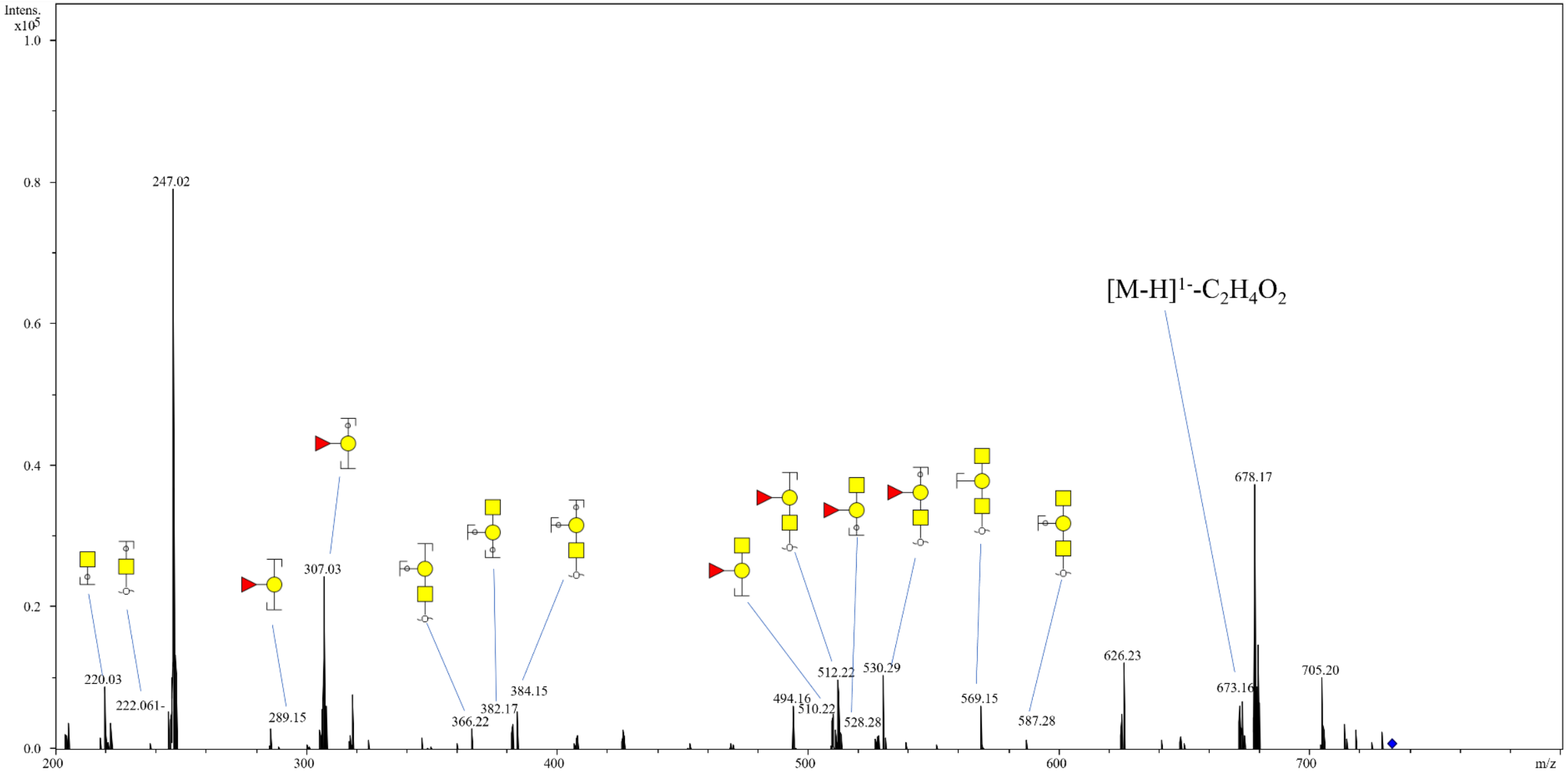

Supporting Figure S1-41: Annotated MS/MS for *O*-glycans released from PSM and PaTu-S cell line (Glycan 41).

Glycan 41

Charge observed: 1-
Theoretical ion: *m/z* 733.29
Observed ion: *m/z* 733.26
Mass deviation: *m/z* 0.03

N2H1F1b

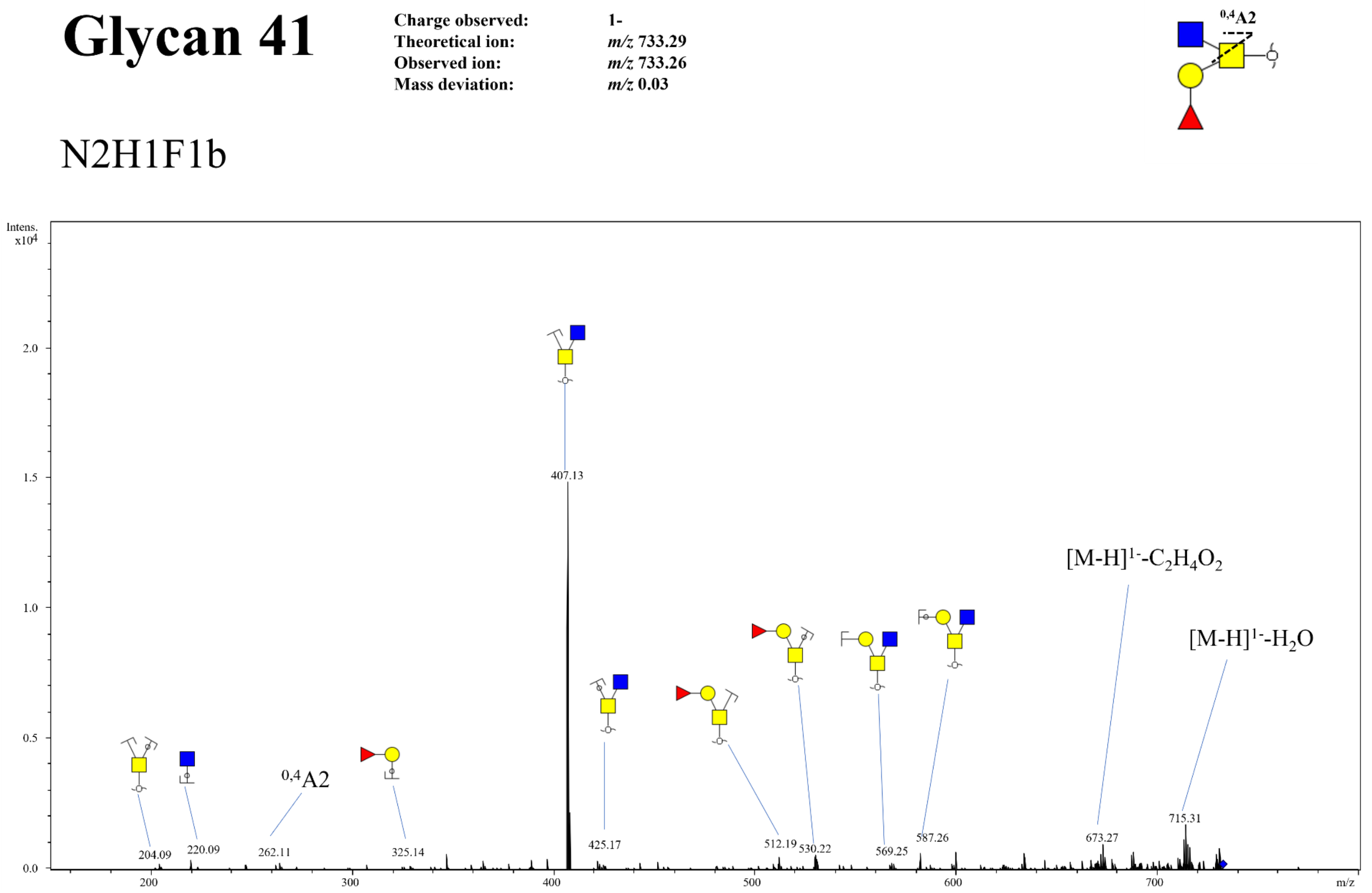

Supporting Figure S1-42: Annotated MS/MS for *O*-glycans released from PSM and PaTu-S cell line (Glycan 42).

Glycan 42

Charge observed: 1-
Theoretical ion: *m/z* 1041.40
Observed ion: *m/z* 1041.39
Mass deviation: *m/z* 0.01

N2H2F2

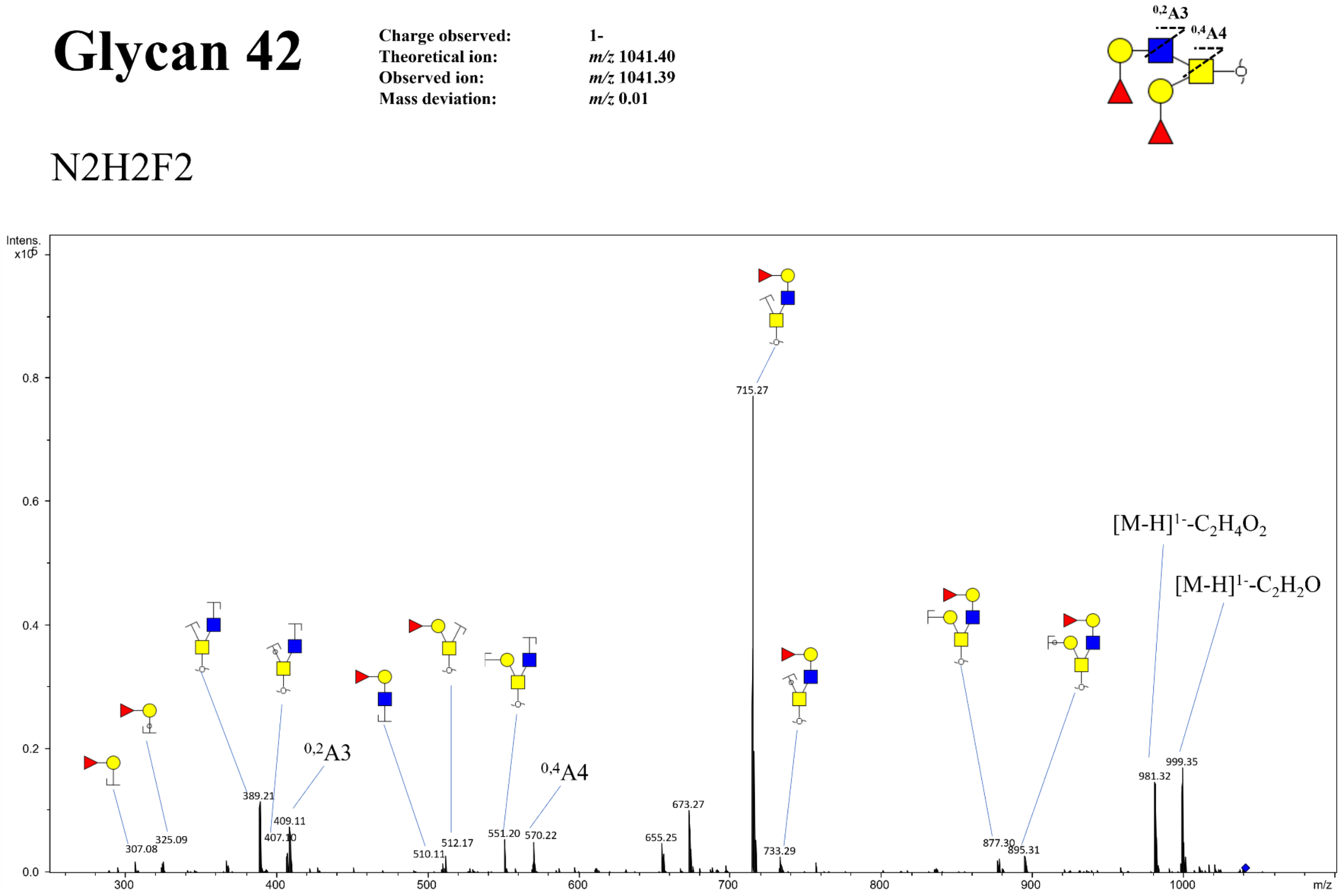

Supporting Figure S1-43: Annotated MS/MS for *O*-glycans released from PSM and PaTu-S cell line (Glycan 43).

Glycan 43

N2H1F1Su1

**Charge observed:**	**1-**
**Theoretical ion:**	***m/z* 813.25**
**Observed ion:**	***m/z* 813.21**
**Mass deviation:**	***m/z* 0.04**

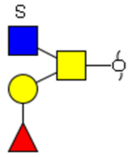

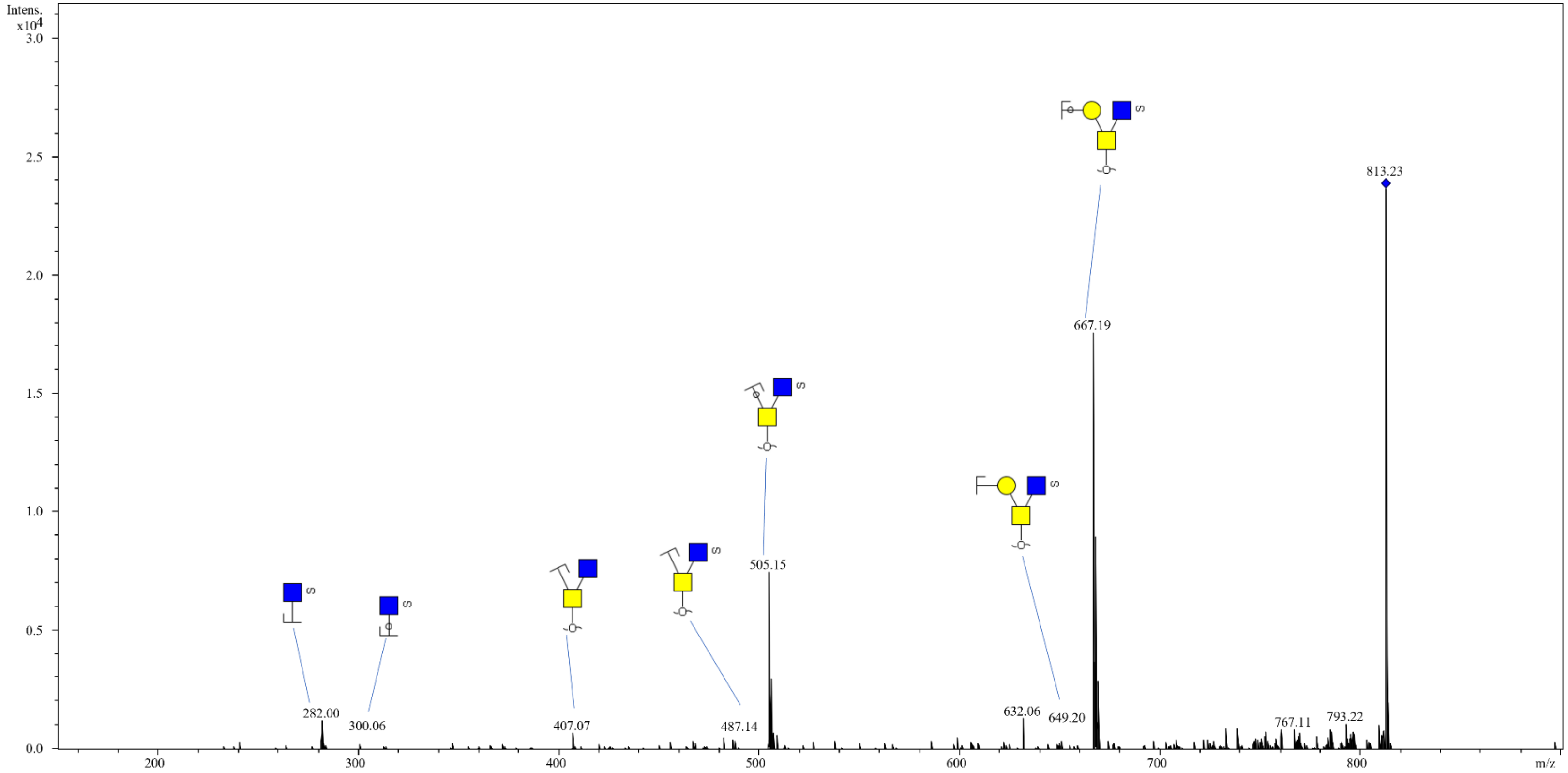

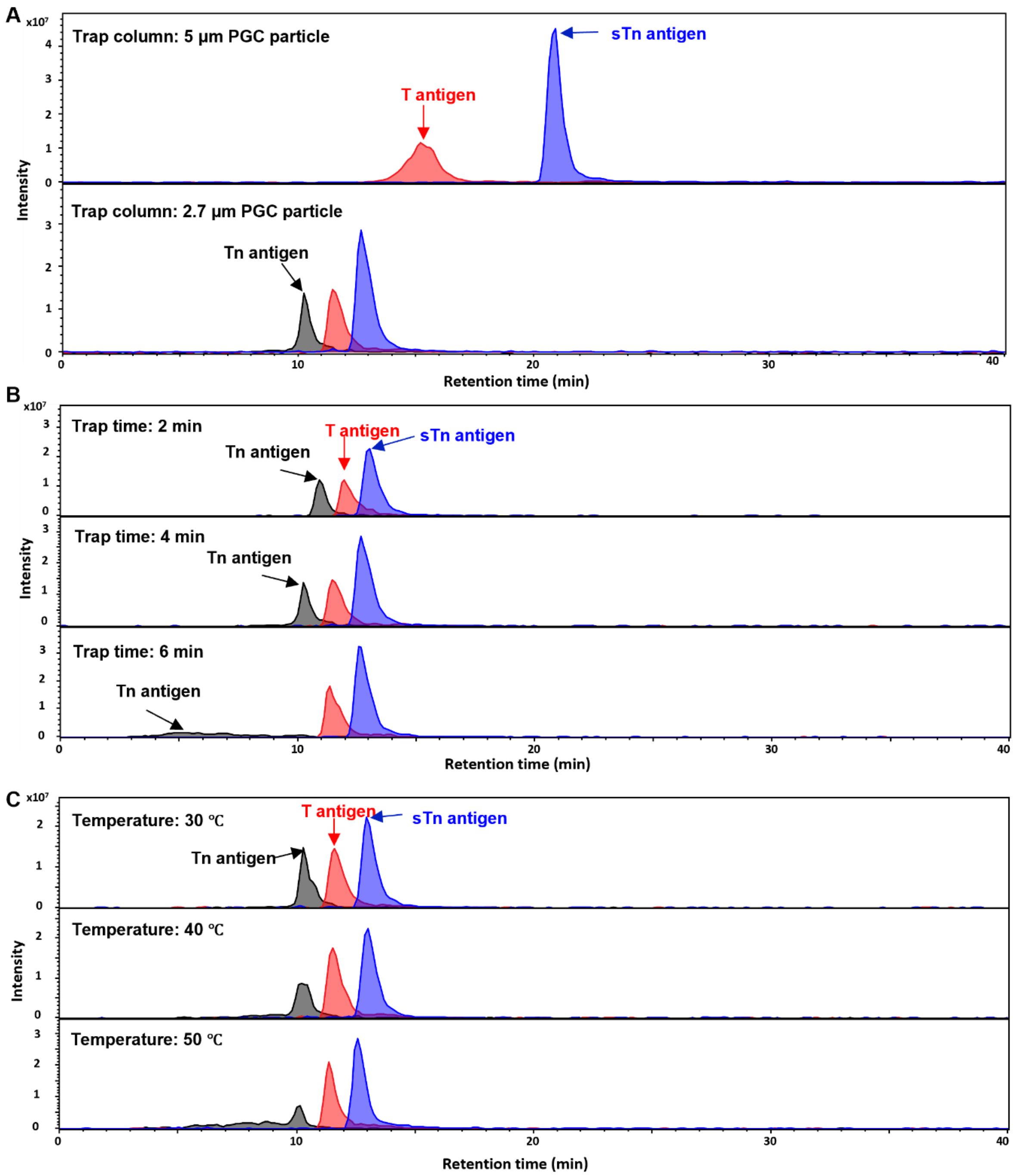

Supporting Figure S2: Chromatographic separation of *O*-glycan alditol standards Tn antigen, T antigen and sTn antigen. **(A)** Improved binding capacity for Tn antigen and chromatographic peak shape for T antigen using 2.7 µm PGC particles compared to 5.0 µm PGC particles (trapping time: 3 min, flow rate: 0.6 µL/min, column temperature: 30 °C). **(B)** Trapping time influences the retention of Tn antigen on the trap column (PGC particles: 2.7 µm, flow rate: 0.6 µL/min, column temperature: 30 °C). **(C)** Elevated temperature decreases the binding of Tn antigen to the trap column (PGC particles: 2.7 µm, flow rate: 0.6 µL/min, trapping time: 3 min). In all experiments, a 4 cm trap column was used with a starting concentration of solvent B of 10%.

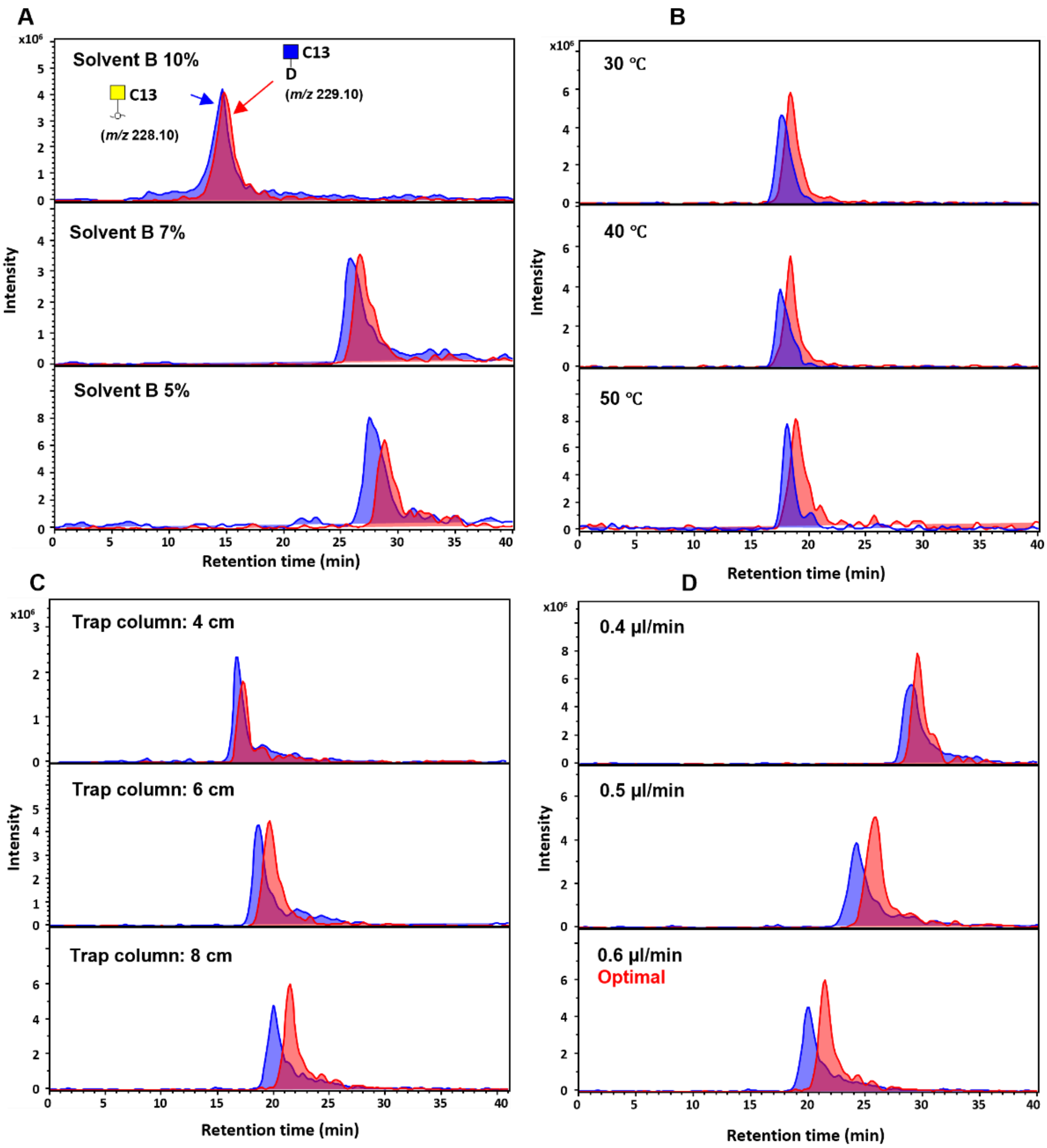

Supporting Figure S3: Chromatographic separation of reduced $^{13}C_6$ isotope labeled GalNAc (blue) and deutero-reduced $^{13}C_6$ isotope labeled GlcNAc (red). **(A)** A lower starting concentration of solvent B increases HexNAcitol separation but results in broader peaks (trap column: 4 cm, flow rate: 0.4 µL/min, column temperature: 30°C). **(B)** Analytical column temperature has only a slight effect on the separation of the HexNAc alditols (trap column: 4 cm, flow rate: 0.6 µL/min, starting concentration solvent B: 10%). **(C)** A longer trap column increases the HexNAc alditol separation (flow rate: 0.6 µL/min, starting concentration solvent B: 5%, column temperature: 30°C). **(D)** A higher flow rate leads to an improved HexNAcitol separation (trap column: 8 cm, starting concentration solvent B: 5%, column temperature: 30°C). In all experiments, 2.7 µm PGC particles and a trapping time of 3 min was used. The final conditions for *O*-glycan analysis include a 8 cm trap column, 3 min trapping time, 5% starting concentration of solvent B, 0.6 µL/min flow rate and an analytical column temperature of 30°C.

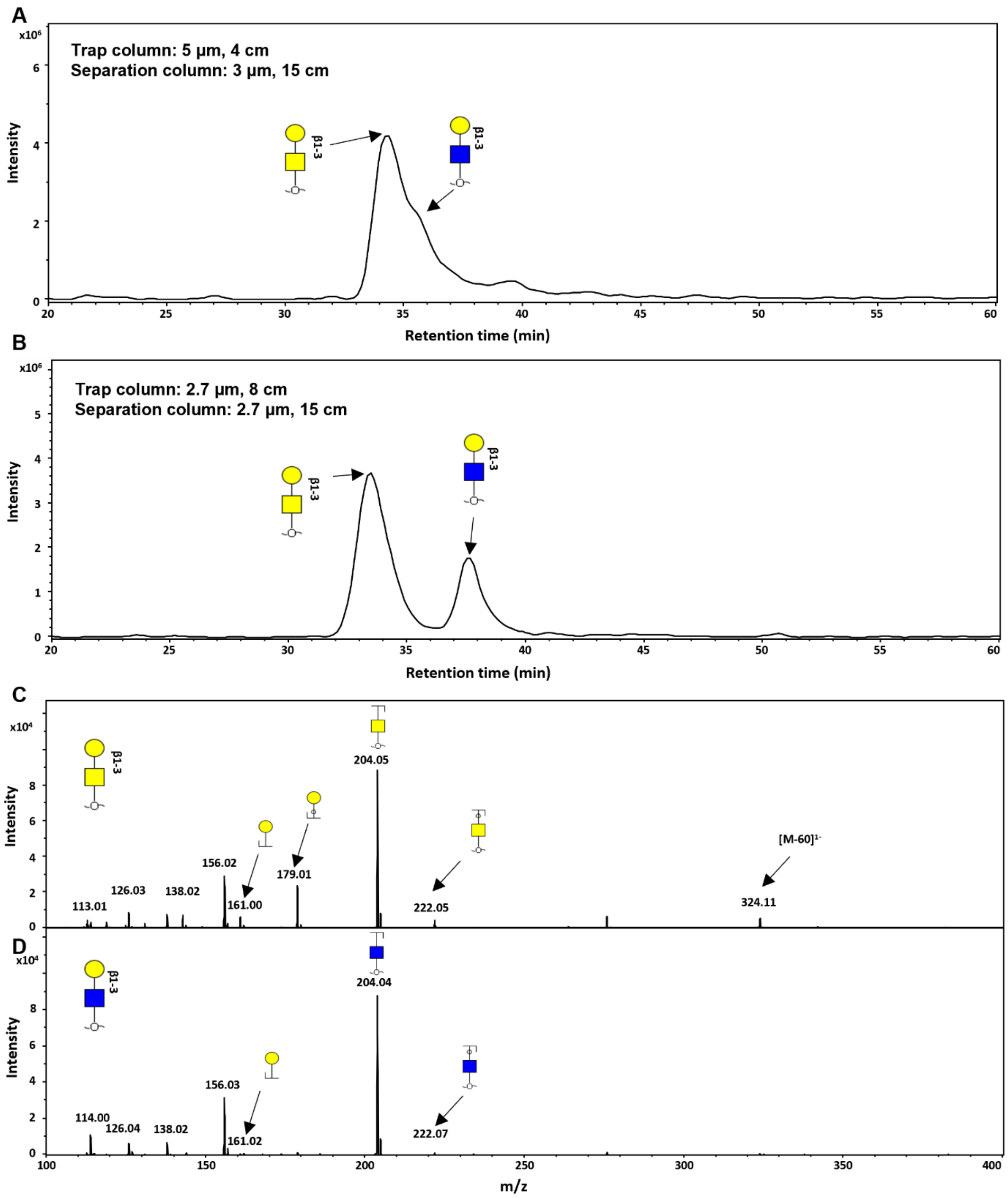

Supporting Figure S4: PGC nano-LC separation of two H1N1 isomers: Galβ1-3GalNAc-ol and Galβ1-3GlcNAc-ol. **(A)** Overlap of Galβ1-3GalNAc and Galβ1-3GlcNAc was observed using the PGC nano-LC-MS/MS platform with a trap column (320 µm x 4 cm) packed with 5 µm PGC particles and analytical column (75 µm x 15 cm) packed with 3 µm PGC particles. **(B)** Improved separation of Galβ1-3GalNAc and Galβ1-3GlcNAc was achieved using a trap column (320 µm x 8 cm) and analytical column (75 µm x 15 cm) packed with 2.7 µm PGC particles. Fragmentation spectra of Galβ1-3GalNAc-ol **(C)** and Galβ1-3GlcNAc-ol **(D)** show a higher abundance of B and C fragments generated from Galβ1-3GalNAc as compared to Galβ1-3GlcNAc.

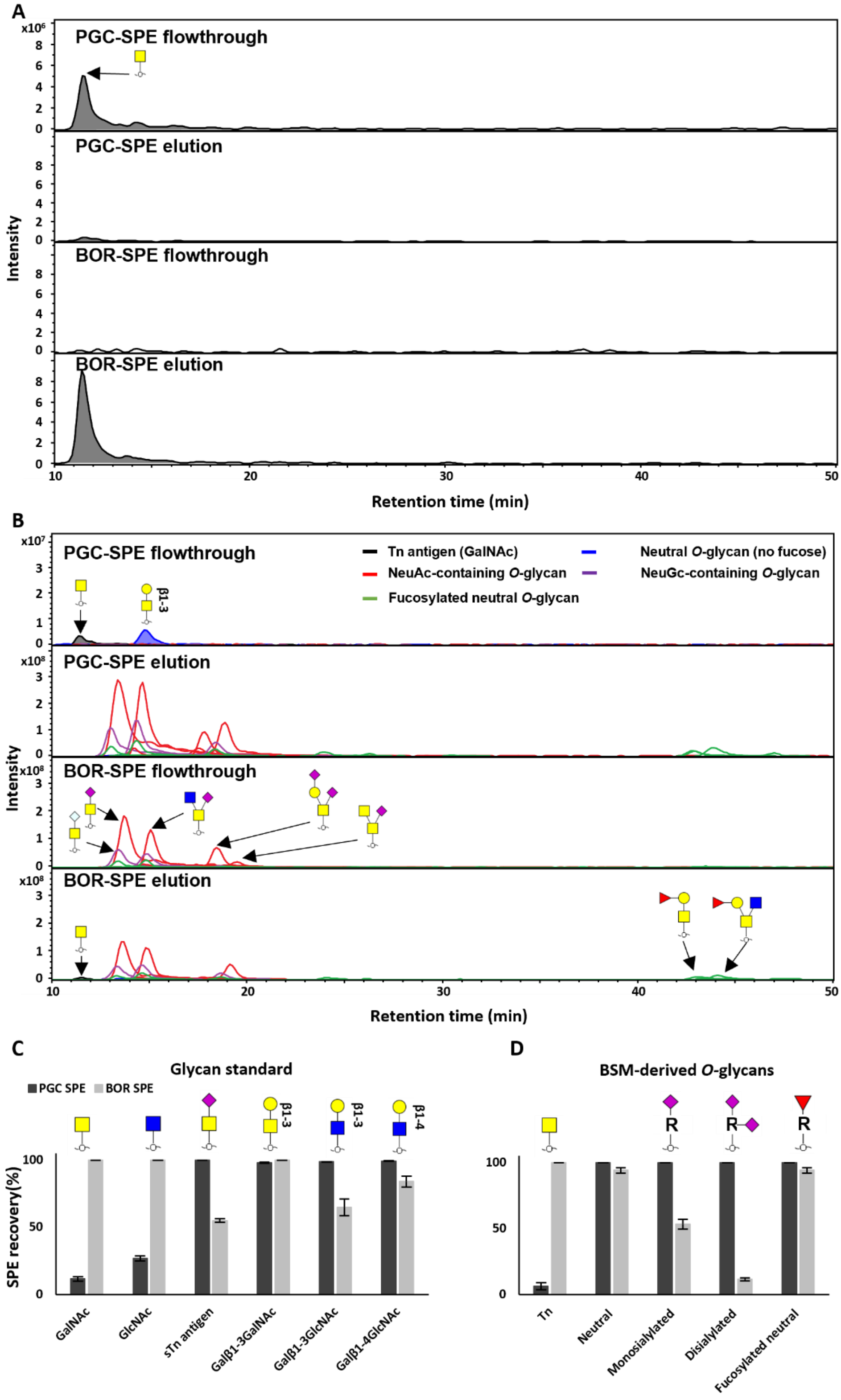

Supporting Figure S5: PGC and BOR SPE of glycan standards, and an *O*-glycan mixture derived from BSM. **(A)** Comparison of PGC SPE and BOR SPE for the purification of the Tn antigen alditol. **(B)** Comparison of PGC SPE and BOR SPE for the purification of the *O*-glycan mixture released from BSM. **(C)** Recovery of PGC SPE and BOR

SPE for the glycan standards Tn antigen, GlcNAc, sTn antigen, T antigen, Galβ1-3GlcNAc and Galβ1-4GlcNAc. **(D)** Recovery of PGC SPE and BOR SPE for the *O*-glycan mixture released from BSM.

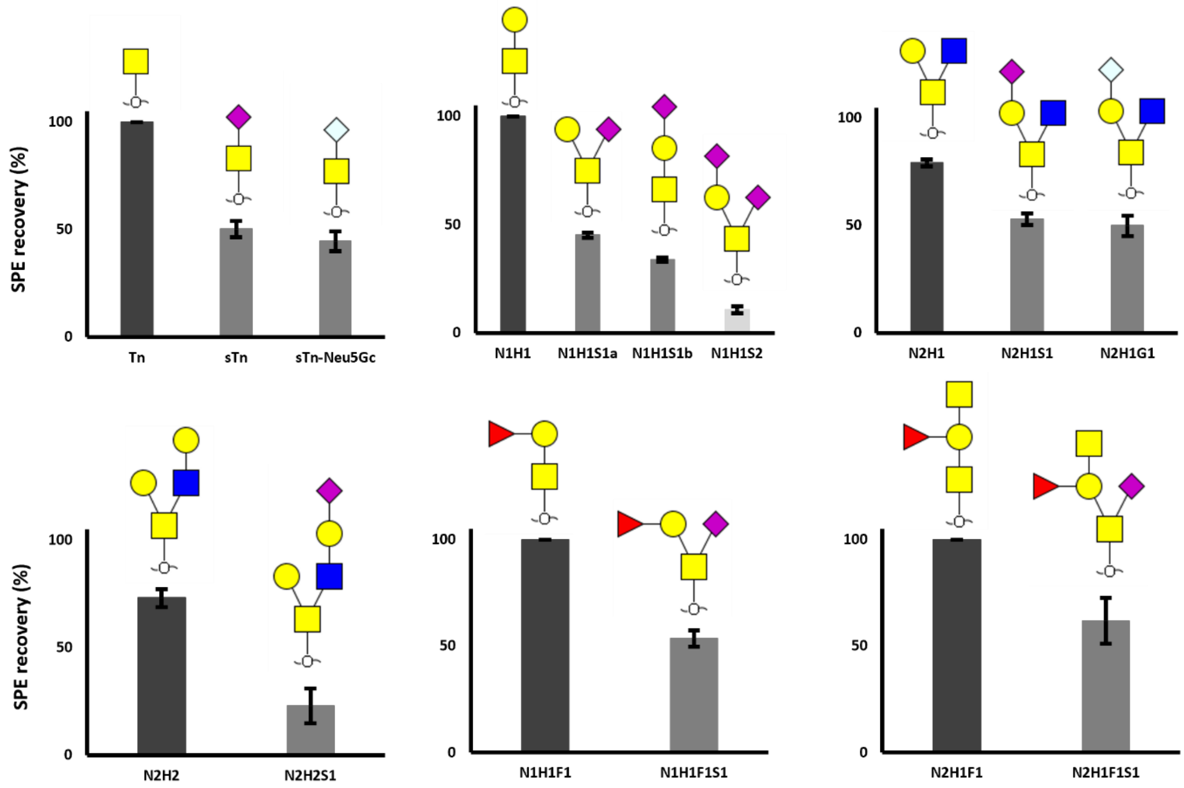

Supporting Figure S6: Presence of sialic acids decreases the BOR SPE recovery of *O*-glycans.

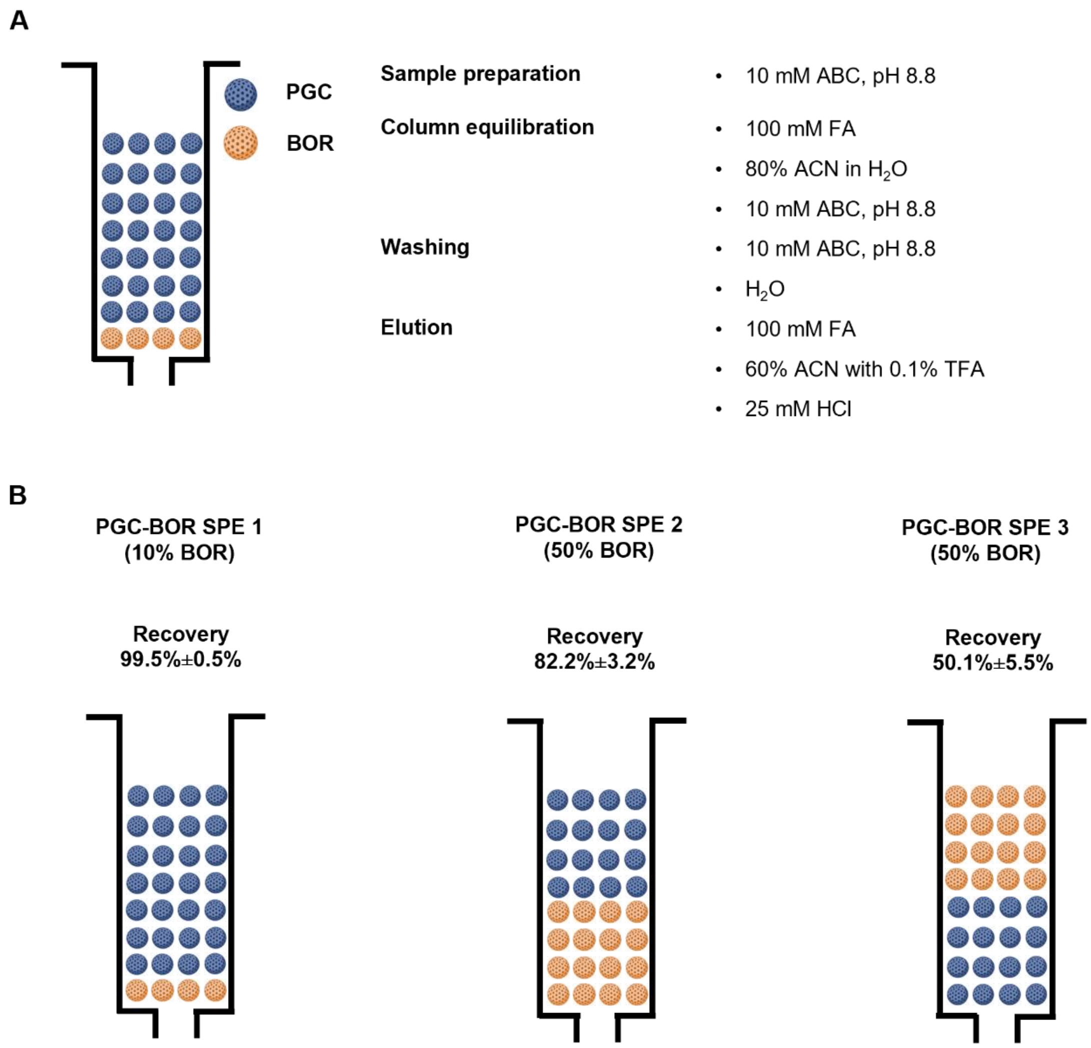

Supporting Figure S7: Design of the mixed-mode PGC-BOR SPE conditions and their recovery, using the *O*-glycan mixture derived from BSM as test sample. **(A)** The final PGC-BOR SPE format used 10% BOR at the bottom and 90% PGC material at the top in one SPE column. The columns were preconditioned by 1 x 100 µL of 100 mM FA, 2 x 100 µL of 80% ACN, and 1 x 100 µL of 200 mM ABC (pH8.8), each time followed by centrifuging at 500 x g for 1 min. The samples were loaded onto the columns and washed 2 x with 100 µL of 200 mM ABC (pH8.8) and 1 x with 100 µL of water by centrifugation at 500 x g for 1min. Next, the *O*-glycan alditols were sequentially eluted by 1 x 100 µL of 100 mM FA, 1 x 100 µL of 60% ACN with 0.1% TFA, and 1 x 100 µL of 25 mM HCl, centrifuging 2 min at 800 x g. **(B)** Other tested packing formats of PGC-BOR SPE lead to lower *O*-glycan recoveries.

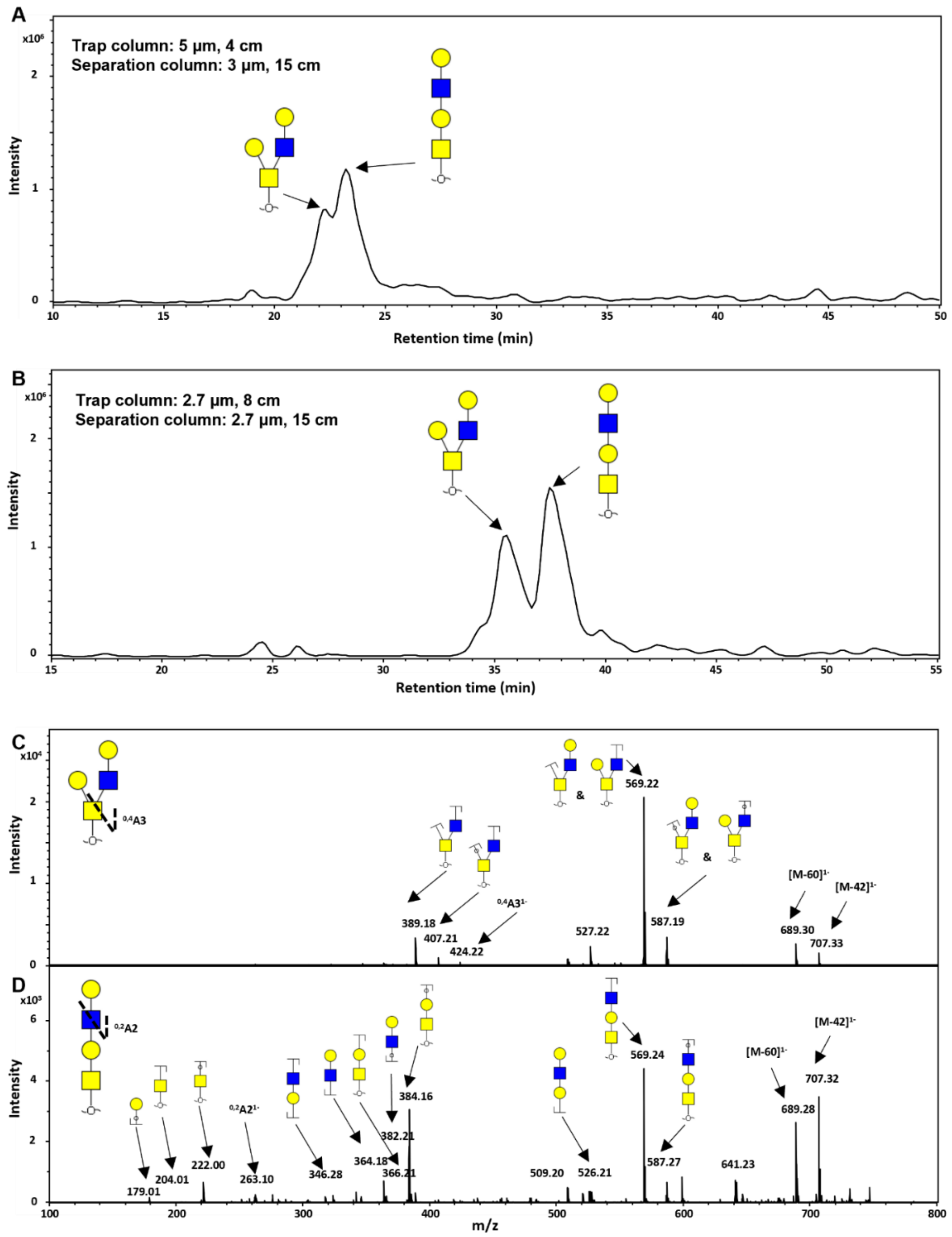

Supporting Figure S8: PGC nano-LC separation of two H2N2 isomers released from PSM. **(A)** Overlap of H2N2 isomers was observed using the PGC nano-LC-MS/MS platform with a trap column (320 µm x 4 cm) packed with 5 µm PGC particles and an analytical column (75 µm x 15 cm) packed with 3 µm PGC particles. **(B)** Improved isomer separation of H2N2 isomers was achieved on the PGC nano-LC-MS/MS platform using a trap column (320 µm x 8 cm) and an analytical column (75 µm x 15 cm) packed with 2.7 µm PGC particles. Fragmentation spectra of the early-eluting H2N2 isomer at 35.2 min **(C)** and the late-eluting H2N2 isomer at 37.8 min **(D)** indicate a core 2 structure for the early-eluting H2N2 and core 1 structure for the late-eluting H2N2.

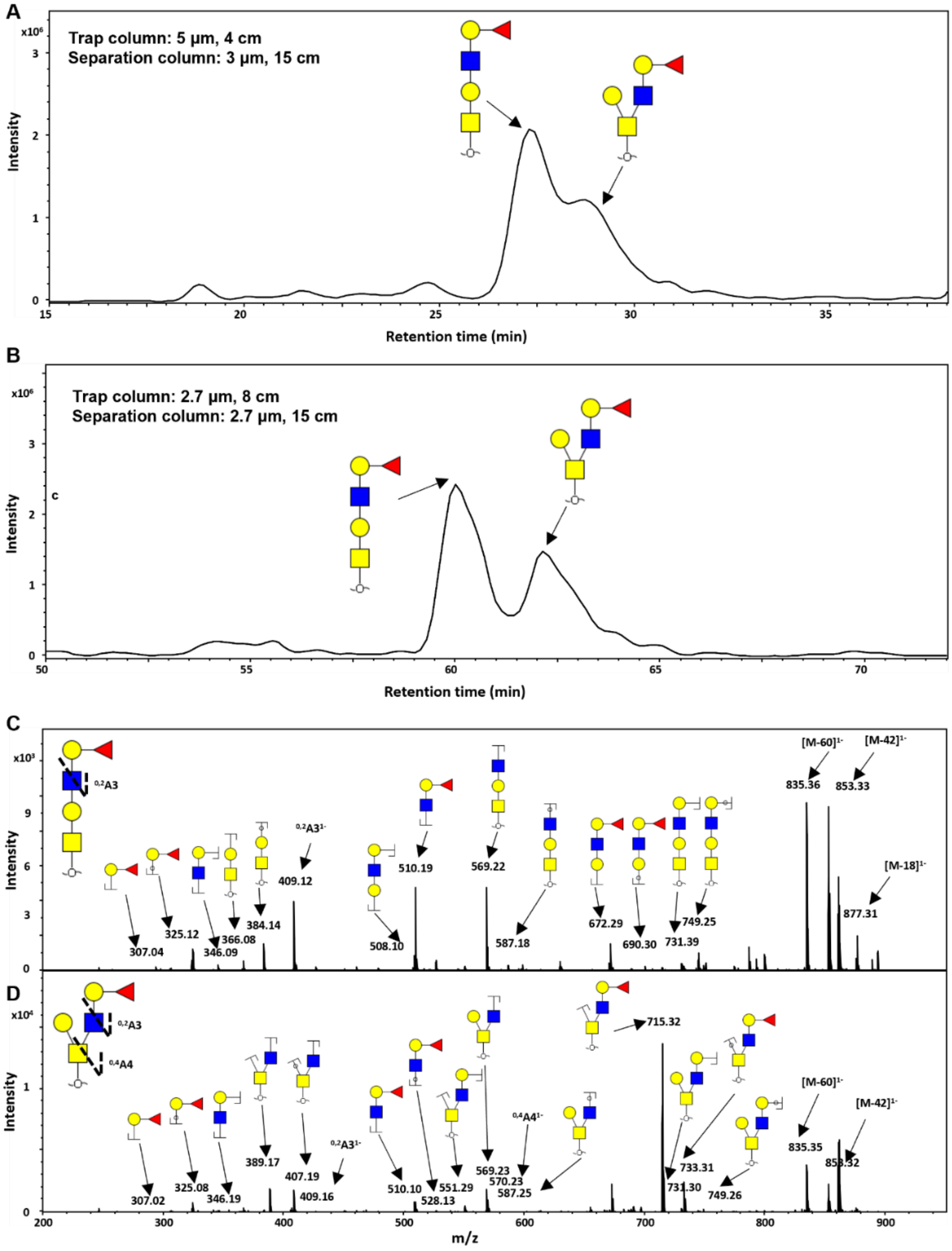

Supporting Figure S9: PGC nano-LC separation of two H2N2F1 isomers released from PSM. **(A)** Overlap of H2N2F1 isomers was observed using the PGC nano-LC-MS/MS platform with a trap column (320 µm x 4 cm) packed with 5 µm PGC particles and analytical column (75 µm x 15 cm) packed with 3 µm PGC particles. **(B)** Improved isomer separation of H2N2F1 isomers was achieved on the PGC nano-LC-MS/MS platform using a trap column (320 µm x 8 cm) and an analytical column (75 µm x 15 cm) packed with 2.7 µm PGC particles. Fragmentation spectra of the early-eluting H2N2F1 isomer at 60.2 min **(C)** and the late-eluting H2N2F1 isomer at 62.3 min **(D)** indicate a core 1 structure for the early-eluting H2N2F1 and core 2 structure for the late-eluting H2N2F1.

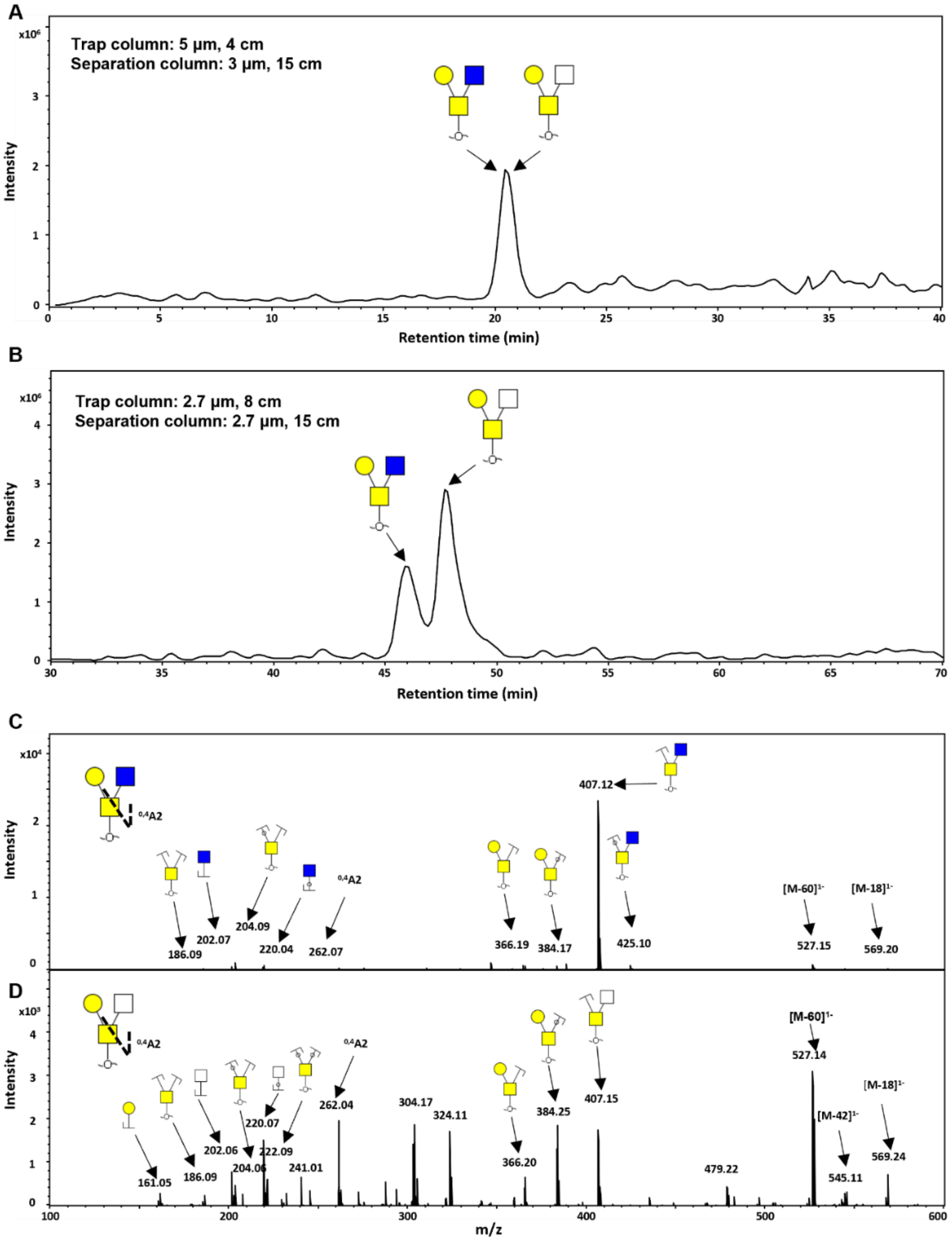

Supporting Figure S10: PGC nano-LC separation of two H1N2 isomers released from the PaTu-S cell line. **(A)** Overlap of H1N2 isomers was observed using the PGC nano-LC-MS/MS platform with a trap column (320 µm x 4 cm) packed with 5 µm PGC particles and an analytical column (75 µm x 15 cm) packed with 3 µm PGC particles. **(B)** Improved isomer separation of the H1N2 isomers was achieved using the PGC nano-LC-MS/MS platform with a trap column (320 µm x 8 cm) and analytical column (75 µm x 15 cm) packed with 2.7 µm PGC particles. The fragmentation spectrum of the early-eluting H1N2 isomer at 46.2 min **(C)** is in line with a core 2 structure. While the fragmentation data of the late-eluting H1N2 isomer at 47.9 min **(D)** is clearly different, this one could not be assigned to a known glycan structure, showing connectivity of both the hexose and the HexNAc to the initiating reduced HexNAc.
